# The histone variant macroH2A1.1 regulates RNA polymerase II-paused genes within defined chromatin interaction landscapes

**DOI:** 10.1242/jcs.259456

**Published:** 2022-04-11

**Authors:** Ludmila Recoules, Alexandre Heurteau, Flavien Raynal, Nezih Karasu, Fatima Moutahir, Fabienne Bejjani, Isabelle Jariel-Encontre, Olivier Cuvier, Thomas Sexton, Anne-Claire Lavigne, Kerstin Bystricky

**Affiliations:** 1 Molecular, Cellular and Developmental Biology (MCD), UMR5077, Centre de Biologie Intégrative (CBI), Université de Toulouse, CNRS, UPS, F-31062 Toulouse, France; 2 Chromatin Dynamics FRM team, CBI, CNRS, UPS, F-31062 Toulouse, France; 3 Institut de Génétique et de Biologie Moléculaire et Cellulaire (IGBMC), CNRS, UMR7104; INSERM U1258; University of Strasbourg, F-67400 Illkirch, France; 4 Institut de Génétique Moléculaire de Montpellier (IGMM), CNRS, UMR5535, F-34293 Montpellier, France; 5 Equipe Labellisée Ligue Nationale contre le Cancer, F-34293 Montpellier, France; 6 Institut Universitaire de France (IUF), 1 rue Descartes, F- 75231 Paris, France

**Keywords:** Histone variants, RNA polymerase II-paused genes, Gene expression, Chromatin structure, Cellular migration, Breast cancer

## Abstract

The histone variant macroH2A1.1 plays a role in cancer development and metastasis. To determine the underlying molecular mechanisms, we mapped the genome-wide localization of endogenous macroH2A1.1 in the human breast cancer cell line MDA-MB-231. We demonstrate that macroH2A1.1 specifically binds to active promoters and enhancers in addition to facultative heterochromatin. Selective knock down of macroH2A1.1 deregulates the expression of hundreds of highly active genes. Depending on the chromatin landscape, macroH2A1.1 acts through two distinct molecular mechanisms. The first mitigates excessive transcription by binding over domains including the promoter and the gene body. The second stimulates expression of RNA polymerase II (Pol II)-paused genes, including genes regulating mammary tumor cell migration. In contrast to the first mechanism, macroH2A1.1 specifically associates with the transcription start site of Pol II-paused genes. These processes occur in a predefined local 3D genome landscape, but do not require rewiring of enhancer-promoter contacts. We thus propose that macroH2A1.1 serves as a transcriptional modulator with a potential role in assisting the conversion of promoter-locked Pol II into a productive, elongating Pol II.

## Abstract (precis)

**Summary:** Histone variant macroH2A1.1 binding to the TSS of genes dependent on Pol II pausing stimulates transcription by promoting Pol II release in a human triple-negative breast cancer cell model.

## INTRODUCTION

Histone post-translational modifications, DNA-binding factors and architectural proteins regulate genome organization and dynamics (Luger et al., 2012; Venkatesh and Workman, 2015). In addition, histone variants replace canonical histones in a locus-specific manner, which endows chromatin with properties required to fine-tune DNA accessibility and functions (Buschbeck and Hake, 2017).

Among the histone variants, macroH2A1 (mH2A1), a vertebrate-specific (Pehrson and Fuji, 1998; Rivera-Casas et al., 2016) histone H2A variant, is composed of an N-terminal ‘H2A-like’ domain (64% identical to H2A) and a C-terminal 25 kDa ‘macro’ domain. These two domains are joined by an unstructured 41 amino acid long ‘linker’ domain that positions the macro domain outside of the nucleosome (Gamble and Kraus, 2010). Expression of the highly conserved *H2AFY* gene (also known as *MACROH2A1*) produces two splicing isoforms, mH2A1.1 and mH2A1.2, the sequences of which differ in a 30 amino acid region within the macro domain (Gamble and Kraus, 2010).

mH2A1 was originally found to be enriched on the transcriptionally silent X chromosome (Costanzi and Pehrson, 1998). mH2A1 is also present at autosomes, forming large domains in association with histone marks associated with heterochromatin, such as H3K27me3 and H3K9me3 (Douet et al., 2017; Gamble et al., 2010; Sun et al., 2018). *In vitro* studies have demonstrated that nucleosomal mH2A1 interferes with binding of the transcription factor NF-κB and inhibits nucleosome sliding by the remodeling complex SWI/SNF and initiation of RNA polymerase II (Pol II) transcription (Angelov et al., 2003; Doyen et al., 2006). Therefore, mH2A1 is generally believed to play a role in transcriptional repression. However, in many cases, the presence of mH2A1 correlates also with active transcription of a subset of genes (Changolkar et al., 2010; Chen et al., 2014; Dell'Orso et al., 2016; Gamble et al., 2010; Podrini et al., 2015; Wan et al., 2017). Thus, the roles of mH2A1 in regulating gene expression are seemingly contradictory and the underlying mechanisms are still not well characterized.

The two mH2A1 splice variants exhibit tissue- and cell-specific expression patterns (Marjanović et al., 2018). In normal cells, the mH2A1.2 isoform appears to be ubiquitously expressed (Cantariño et al., 2013; Sporn et al., 2009; Sporn and Jung, 2012), whereas mH2A1.1 is only expressed in differentiated cells with low proliferation rates (Cantariño et al., 2013; Sporn et al., 2009; Sporn and Jung, 2012). Notably, the mH2A1.1 macro domain can bind NAD^+^ metabolites (Kustatscher et al., 2005) and interact with the DNA-damage repair and chromatin remodeling factor PARP1 [poly-(ADP-ribose) polymerase 1] (Chen et al., 2014; Marjanović et al., 2018; Ouararhni et al., 2006; Ray Chaudhuri and Nussenzweig, 2017). Interaction between mH2A1.1 and PARP1 has been shown to be important during DNA damage, stress responses (Kim et al., 2018; Xu et al., 2012), mitochondrial respiratory capacity (Marjanović et al., 2018) and transcription (Chen et al., 2014; Gamble et al., 2010; Ouararhni et al., 2006) in fibroblasts and epithelial cancerous cells. In tumors, expression of the mH2A1.1 isoform is frequently reduced compared with normal tissues, suggesting that this isoform is a tumor suppressor (Cantariño et al., 2013; Lavigne et al., 2014; Sporn and Jung, 2012). Interestingly, in immortalized human mammary epithelial cells, mH2A1 interferes with the epithelial-mesenchymal transition (EMT), and its reciprocal, the mesenchymal-epithelial transition (MET) processes required for metastasis development (Hodge et al., 2018; Pliatska et al., 2018). However, in highly metastatic cancers, such as triple-negative breast cancers (TNBCs), increased expression levels of mH2A1.1 correlate with poor prognosis (Lavigne et al., 2014). The role of macroH2A1.1 in controlling the properties of tumor cells could be dependent on cellular context and remains to be clarified.

In this work, we identified and characterized the role of mH2A1.1 in the regulation of gene expression in TNBC cells. We found that mH2A1.1 modulates the expression of hundreds of highly expressed genes, but mH2A1.1 deficiency does not affect the expression of silent or low expressed genes. Many of these mH2A1.1-regulated genes are involved in cytoskeletal organization, conveying a role for mH2A1.1 in controlling the migratory properties of these tumor cells. This role of mH2A1.1 is, however, bifunctional because mH2A1.1 can have either an inhibitory or a stimulating effect on target gene transcription. Although we found no evidence for ad hoc rewiring of promoter-enhancer contacts, this functional dichotomy clearly depends on the chromatin landscape in which these genes are located and relies on differential recruitment of mH2A1.1. The activating effect of mH2A1.1 requires tight recruitment of mH2A1.1 to the transcription start site (TSS) of related genes. Conversely, genes inhibited by mH2A1.1 recruit this histone variant over larger domains, present further upstream and downstream of the TSS. Mechanistically, we determined that the expression level of mH2A1.1-activated genes is dependent on Pol II pausing. mH2A1.1 appears to regulate Pol II turnover at the TSS by inhibiting the release of paused Pol II. Linking differential recruitment of mH2A1.1 to mode of action, our work clarifies the ambivalence of the roles attributed to mH2A.1.1 in transcription regulation in cancer cells.

## RESULTS

### mH2A1.1 regulates expression of hundreds of genes

**Fig. 1.**
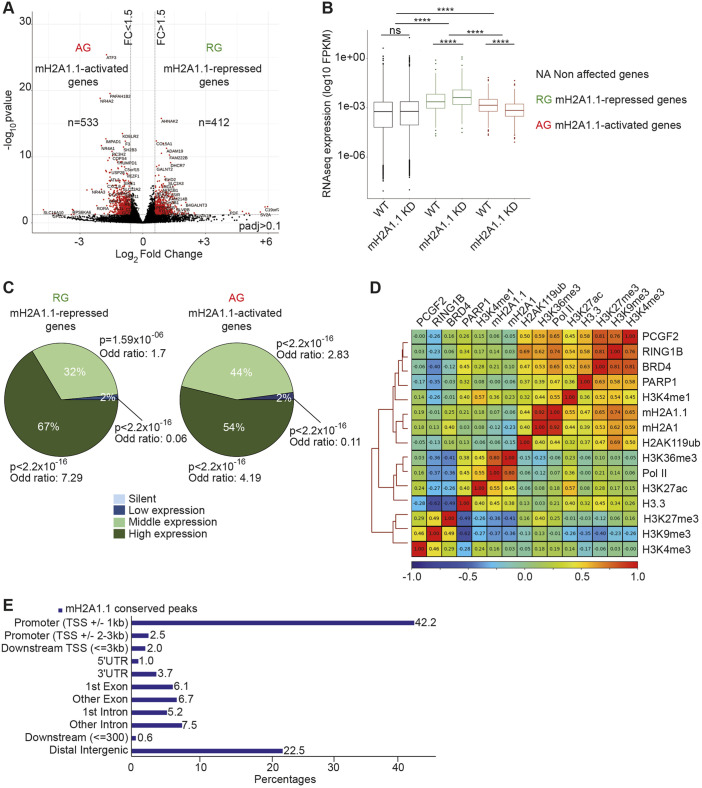
**The histone variant mH2A1.1 regulates expression of hundreds of genes in MDA-MB 231 cells.** (A) Volcano plot showing fold change of gene expression in mH2A1.1 KD compared with WT MDA-MB-231 cells. Red dots represent significantly deregulated genes with a fold change >1.5 and *P*-adj<0.1. (B) Boxplot comparing gene expression (FPKM) of the indicated genes between control (WT) and mH2A1.1 KD conditions. The box represents the 25–75th percentiles, and the median is indicated. Whiskers extend to 25th percentile minus 1.5× IQR and 75th percentile plus 1.5× IQR. Wilcoxon tests were used to compare conditions. ****P<2.2×10^−16^; ns, non-significant. (C) Pie charts showing proportion of mH2A1.1-regulated genes in four groups categorized by gene expression levels in control cells, as indicated. Enrichment of mH2A1.1 target genes with categories of genes was measured using Fisher exact tests. *P*-values and the odds ratios are shown. (D) Whole-genome Spearman correlation heatmap of mH2A1.1, mH2A1 and a series of histone modifications and chromatin-associated factor ChIP-seq data, as indicated. Pearson coefficient correlations (PCC, r) are given. Red and blue colors denote high correlation (r close to 1) and anti-correlation (r close to −1), respectively. (E) Proportions of different genomic features associated with mH2A1.1 conserved peaks. mH2A1.1 ‘conserved’ peaks correspond to the common peaks between mH2A1.1 specific ChIP-seq and mH2A1 ChIP-seq and were used for further analysis.

In order to characterize the function of mH2A1.1 in TNBC, we performed RNA sequencing (RNA-seq) in the MDA-MB-231 cell line, which expresses mH2A1.1 at higher levels than other types of breast cancer cell lines (Fig. S1A,B) (Lavigne et al., 2014). We compared gene expression levels between wild-type (WT) cells and cells in which the mH2A1.1 isoform, but not mH2A1.2 protein expression, was abolished by RNA interference (RNAi) (termed KD) (Fig. 1A, Fig. S1C-E). Among the 945 genes for which expression was significantly modified in the mH2A1.1 KD cells, 533 genes (56.3%) were downregulated (called mH2A1.1-activated genes or AGs) and 412 genes (43.7%) were upregulated (called mH2A1.1-repressed genes or RGs) (Fig. 1A, Tables S1 and S2). Altered expression of a subset of genes was confirmed by RT-qPCR using two different siRNAs directed against mH2A1.1 (Fig. S1F-H). All mH2A1.1-regulated genes, both RGs and AGs, were found among the moderately to highly expressed genes in WT MBA-MB231 cells (Fig. 1B,C). Silenced genes in MDA-MB-231 cells were not activated upon mH2A1.1 depletion (Fig. 1B,C). We concluded that mH2A1.1 participates in fine-tuning of actively transcribed gene expression. We next investigated whether the role of the mH2A1.1 variant in controlling expression of active genes depends on its association with specific genomic regions, including gene regulatory regions.

### mH2A1.1 associates with gene regulatory regions

We developed a chromatin immunoprecipitation (ChIP)-grade polyclonal antibody that exclusively recognizes mH2A1.1 (Ab αmH2A1.1) (Table S4, Fig. S2A-D) and generated ChIP-seq data for mH2A1.1 (Table S5). The obtained dataset was compared with the one obtained using a commercially available ChIP-grade antibody (Ab37264, Ab αmH2A1) directed against total mH2A1 (Tables S4 and S5, Fig. S2E-G). The two datasets were highly similar with a Pearson coefficient correlation (PCC) of 0.92 (Fig. 1D). Therefore, we decided to conserve common peaks between the two ChIP-seq data for further analysis (Materials and Methods). We identified 11.849 mH2A1.1 peaks, covering ≈7% of the genome. Analysis of the genomic distribution of mH2A1.1 showed that the vast majority of mH2A1.1 peaks corresponds to annotated promoters (TSS±1 kb), whereas 22% of mH2A1.1 peaks were associated with distal intergenic regions (Fig. 1E). We confirmed the enrichment of mH2A1.1 in a subset of regions corresponding to ChIP-seq peaks by ChIP-qPCR in WT cells, as well as its decrease in mH2A1.1 KD cells (two mH2A1.1-specific siRNAs), using either mH2A1.1- or mH2A1-specific antibodies (Fig. S2H,I).

### mH2A1.1 binds promoters of its target genes

We next examined whether mH2A1.1 binding occurred in specific chromatin environments. We analyzed the correlation between mH2A1.1 and a subset of heterochromatin marks (H3K9me3, H3K27me3, H2AK119ub), chromatin-bound components [Pol II, BRD4, RING1B (RNF2), PARP1, PCGF2] as well as euchromatin marks (H3K4me1, H3K4me3, H3K36me3, H3K27ac, H3.3) at a genome-wide level (Fig. 1D). Binding of mH2A1.1 and of its well-documented partner PARP1 correlated positively in the MDA-MB-231 cell line (Fig. 1D) (PCC of 0.47) (Chen et al., 2014). As expected, mH2A1.1 (but mainly total mH2A1) also associated with broad H3K27me3-marked chromatin domains (PCC of 0.25 for mH2A1.1 and 0.40 for mH2A1) (Fig. 1D, Fig. S3A,B). H3K9me3 marks, known to be over-represented in this TNBC cell line relative to other cancer cell lines (Segal et al., 2018; Yokoyama et al., 2013), overlapped with mH2A1.1 binding at H3K27me3-marked domains (Fig. S3A). Interestingly, we found that in heterochromatin domains levels of H3K9me3 and H3K27me3 tended to be inversely proportional (Fig. S3B,C). Moreover, we found that high H3K27me3-H3K9me3 difference was mainly associated with genomic regions whereas low H3K27me3-H3K9me3 difference characterized intergenomic regions (Fig. S3D). We propose that, in this cell line, the ratio between H3K27me3 and H3K9me3 could be used to distinguish ‘facultative-like’ heterochromatin and ‘constitutive-like’ heterochromatin. We found that mH2A1.1 and PARP1 binding were proportional to the relative abundance of H3K27me3 versus H3K9me3 (Fig. S3E), indicating that these two proteins predominantly associate with ‘facultative-like heterochromatin’ in the MDA-MB-231 TNBC cell line.

**Fig. 2.**
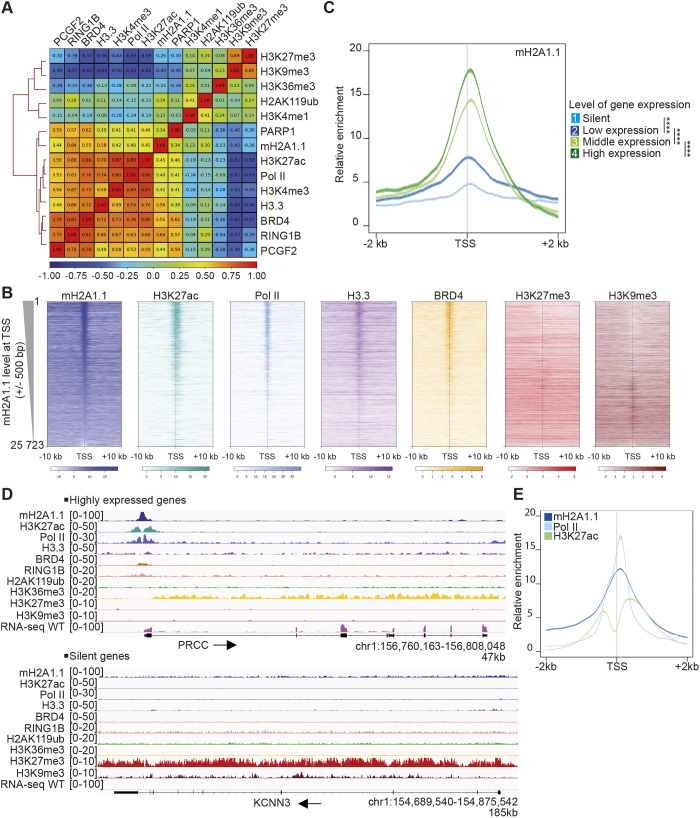
**mH2A1.1 is recruited to promoters of active genes.** (A) TSS (±1 kb)-centered Spearman correlation heatmap of ChIP-seq data. Correlations shown as in Fig. 1D. (B) Profiles of relative enrichment around the TSS (±10 kb) of the indicated ChIP-seq data at human annotated genes (*n*=25,723) ranked according to the mH2A1.1 level at the TSS (±500 bp). Color intensity reflects the level of ChIP-seq enrichment. Heatmaps are oriented with gene bodies placed on the right of the heatmap. (C) Metagene profiles of the average (±s.e.m.) of mH2A1.1 enrichment at TSSs (±2 kb) categorized in four groups according to gene expression levels measured using RNA-seq data. Results of statistical difference analysis between indicated groups are shown on the TSS (±500 bp). Wilcoxon tests were used to compare conditions. ****P<2.2×10^−16^. (D) Genome browser views of indicated ChIP-seq illustrating the binding of mH2A1.1 to the promoter region of a transcribed gene in an open chromatin state (top) and its absence on a silent gene in a closed chromatin state (bottom). Unstranded RNA-seq signal is also shown. Black arrows indicate direction of transcription. (E) Metagene profile of average (±s.e.m.) of mH2A1.1, Pol II and H3K27ac enrichment at the TSS (±2 kb) of transcribed genes (see C, groups 2-4).

When specifically examining the chromatin landscape at promoters (TSS±1 kb), we found that mH2A1.1 enrichment correlated with H3K4me3 and H3K27ac, as well as with BRD4, H3.3 and Pol II binding (Fig. 2A,B), suggesting a role for mH2A1.1 in transcription initiation-regulated processes. At promoters, mH2A1.1 distribution inversely coincided with heterochromatin marks (Fig. 2A,B). We further determined that enrichment of mH2A1.1 centered at the TSS was proportional to the level of transcription (Fig. 2C,D). The profile of mH2A1.1 binding was greatest at the TSS of expressed genes. This profile was similar to, albeit larger than, that of Pol II at the nucleosome-free region, bordered by H3K27ac-marked nucleosome regions (Fig. 2E).

**Fig. 3.**
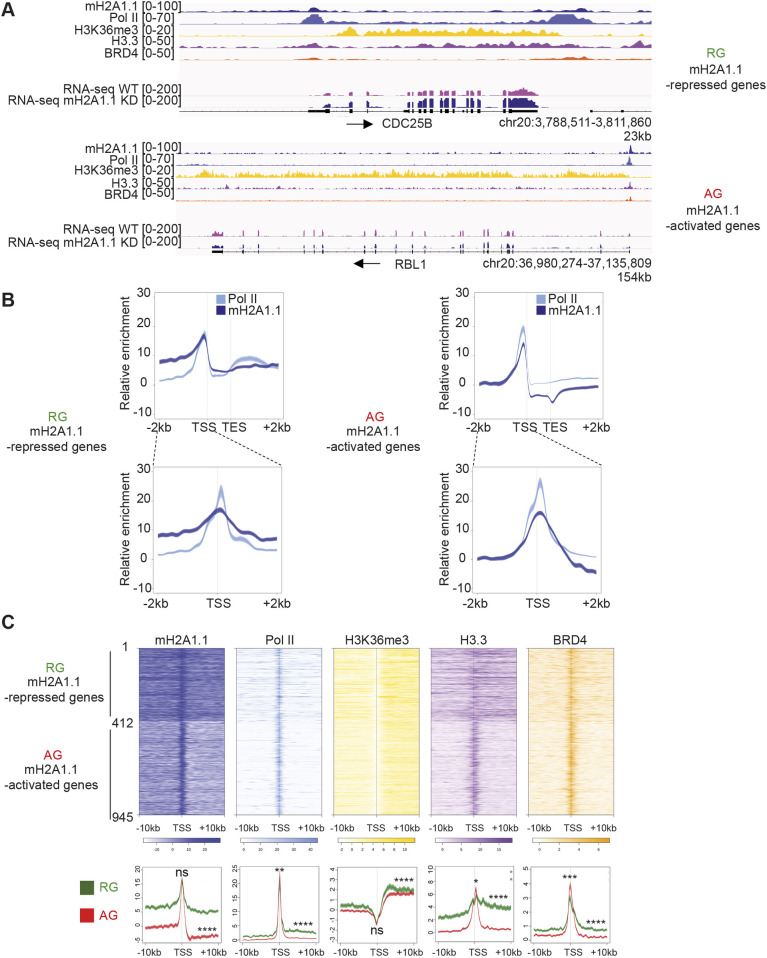
**The chromatin landscapes of mH2A1.1-regulated genes.** (A) Genome browser views of ChIP-seq of an mH2A1.1-repressed gene (top) and an mH2A1.1-activated gene (bottom). Unstranded RNA-seq signals in control and mH2A1.1 KD are also shown. Black arrows indicate direction of transcription. (B) Metagene profiles of average (±s.e.m.) of mH2A1.1 and Pol II enrichment at mH2A1.1-regulated genes (TSS-TES±2 kb) and at the TSS of mH2A1.1-regulated genes (TSS±2 kb). (C) Top: Heatmap profiles showing the relative enrichment of the indicated proteins and histone modifications around the TSS (±10 kb) of mH2A1.1-regulated genes (see Fig. 1A). The top half shows mH2A1.1-repressed genes (1-412, *n*=412), and the bottom half mH2A1.1-activated genes (412-945, *n*=533). Color intensity reflects the level of ChIP-seq enrichment. Heatmaps are oriented. Bottom: Metagene profiles of average (±s.e.m.) of the indicated ChIP-seq data around the TSS (±10 kb) of mH2A1.1-regulated genes. Average profiles around the TSS of mH2A1.1-repressed genes are shown in green whereas average profiles around the TSS of mH2A1.1-activated genes are shown in red. Results of statistical difference analysis between these two groups are shown, either on the TSS (±50 bp) or on the gene body (+50 bp – TES). Wilcoxon tests were used to compare conditions. ns, not significant*.* *P<0.05, **P<0.01, ***P<0.001, ****P<2.2×10^−16^.

We next questioned whether this profile was linked to the mechanism by which mH2A1.1 regulates gene expression. To this end, we separately determined mH2A1.1 binding at genes repressed or activated (RGs and AGs) by mH2A1.1. At both gene categories, mH2A1.1 was highly enriched at the TSS (Fig. 3A,B). However, mH2A1.1 binding was restricted to the TSS of mH2A1.1 AGs, whereas it associated both with promoter regions and with gene bodies of mH2A1.1 RGs (Fig. 3A,B). mH2A1.1 association correlated with the level of binding of Pol II, H3.3 and BRD4 (Fig. 3C). Interestingly, at RGs we detected Pol II at the promoter and over the elongation-characteristic H3K36me-marked gene body. At AGs, Pol II binding was essentially limited to the TSS (Fig. 3). Thus, Pol II distribution also discriminates between types of mH2A1.1-regulated genes. Of note, RING1B, PCGF2 and H2AK119ub (i.e. PRC1 subunits and associated modification), were bound to genomic regions of expressed RGs (Fig. S3F).

### mH2A1.1 promotes gene expression of Pol II-paused genes

**Fig. 4.**
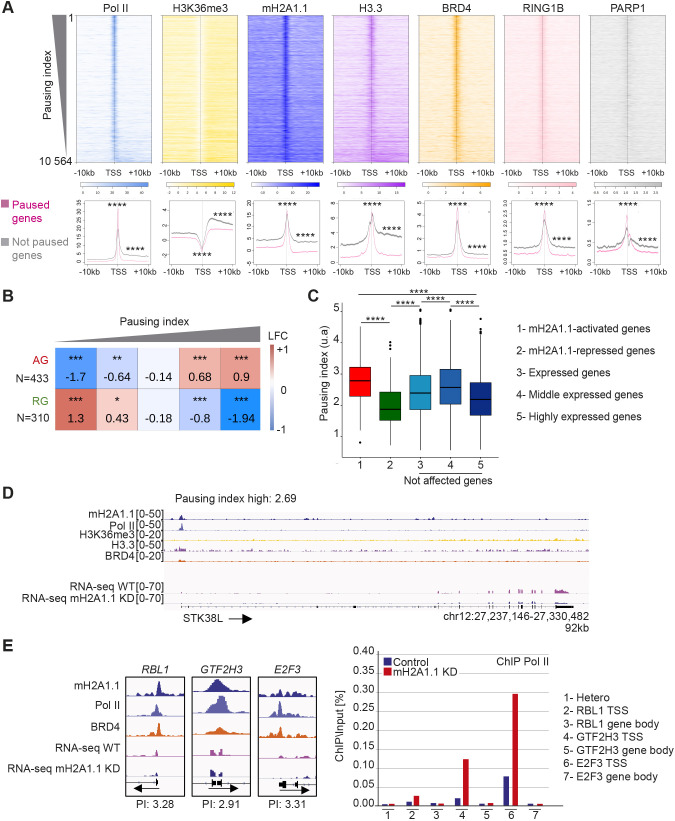
**mH2A1.1-activated genes are regulated by Pol II pausing.** (A) Top: Heatmap profiles showing enrichment of indicated factors and modifications around the TSS (±10 kb) of transcribed genes (*n*=10,198) ranked by their PI. Color intensity reflects the level of ChIP-seq enrichment. Heatmaps are oriented. Bottom: Metagene profiles of average (±s.e.m.) of the indicated ChIP-seq data around the TSS (±10 kb) of paused and not paused genes, as indicated in pink and gray, respectively. Genes are considered as paused if their PI is >2 (*n*=7208). Genes are considered as ‘not paused’ if PI<2 (*n*=3356). Results of statistical difference analysis between these two groups are shown, either on the TSS (±50 bp) or on the gene body (+50 bp – TES). Wilcoxon tests were used to compare conditions. ****P<2.2×10^−16^. (B) Fisher test heatmap showing enrichment of indicated mH2A1.1-target genes with genes divided into five equal-sized categories as a function of their PI. Asterisks indicate the significance of the Fisher exact tests; color map and values present in each square highlight the log2 odds ratio (LOR) of the Fisher exact test. *N* indicates the number of genes used for the analysis. (C) Boxplot comparing the PI of the indicated groups of genes. (Gene number in each group: 1, *n*=433; 2, *n*= 310; 3, *n*= 9 645; 4, *n*=5 176; 5, *n*=4.469). The box represents the 25–75th percentiles, and the median is indicated. Whiskers extend to 25th percentile minus 1.5× IQR and 75th percentile plus 1.5× IQR. Wilcoxon tests were used to compare conditions. ****P<2.2×10^−16^. Only genes characterized by a PI were used. (D) Genome browser view of the indicated ChIP-seq on a paused gene. Unstranded RNA-seq signals in control and mH2A1.1 KD conditions are shown. Black arrows indicate direction of transcription. Only genes characterized by a PI were used. (E) Left: Genome browser view of ChIP-seq of three mH2A1.1-activated genes (*RBL1*, *GTF2H3*, *E2F3*). These genes are considered as paused genes with PIs of 3.28, 2.9 and 3.2, respectively. Right: ChIP-qPCR of Pol II on WT and mH2A1.1-depleted cells. ‘Hetero’ corresponds to a negative position. For each gene, Pol II enrichment was evaluated on the TSS and a gene body region. Results from additional biological replicates are given in Fig. S4D.

The binding pattern of Pol II at mH2A1.1 AGs was reminiscent of Pol II-paused genes (Adelman and Lis, 2012). To confirm this, we calculated the Pol II pausing index (PI) for transcribed genes using Pol II ChIP-seq data as described by Adelman and Lis (2012). Briefly, the PI corresponds to the ratio between total Pol II density in the promoter-proximal region (from −30 bp to +300 bp around the TSS) and total Pol II density in the transcribed region [from +300 bp downstream of the TSS to the transcription end site (TES)]. We plotted the ChIP-seq signal of Pol II and H3K36me3 around the TSS±10 kb for each gene ranked according to their PI (Fig. 4A). In agreement with the literature (Elrod et al., 2019), the level of H3K36me3 was greater over the body of genes with low PI compared with genes with high PI. We further observed that confinement of mH2A1.1 to the TSS and its absence from the gene body was characteristic of genes with a high PI (Fig. 4A). In agreement, the width of mH2A1.1 peaks overlapping with TSSs, as well as that of Pol II peaks, correlated negatively with the PI (Fig. S4A,B). H3.3 follows the same binding profile as mH2A1.1. BRD4, RING1B and PARP1 were mainly enriched at the TSS, and slightly more at high-PI genes (Fig. 4A).

The majority of mH2A1.1-AGs (85%) had a PI >2 (Fig. S4C), a PI value that can be used as a threshold to distinguish paused from not paused genes (Day et al., 2016) (Table S3). In agreement, the average PI of these genes was significantly higher than any other gene category tested (Fig. 4B-D). In contrast, RGs were enriched in low-PI genes (Fig. 4B,C). Furthermore, ChIP-qPCR analysis of Pol II at three mH2A1.1-activated genes (*RBL1*, *GTF2H3* and *E2F3*) showed that the amount of Pol II at promoter regions was increased by approximately threefold upon siRNA reduction of mH2A1.1 (Fig. 4E, Fig. S4D). On average, we observed that depletion of mH2A1.1 induces an increase in the PI (ratio between promoter region and gene body) of *RBL1*, *GTF2H3* and *E2F3* genes by factors of 1.4, 1.6 and 1.7, respectively. These results suggest that mH2A1.1 enhances gene expression by promoting Pol II pause release.

### mH2A1.1 binds enhancers

**Fig. 5.**
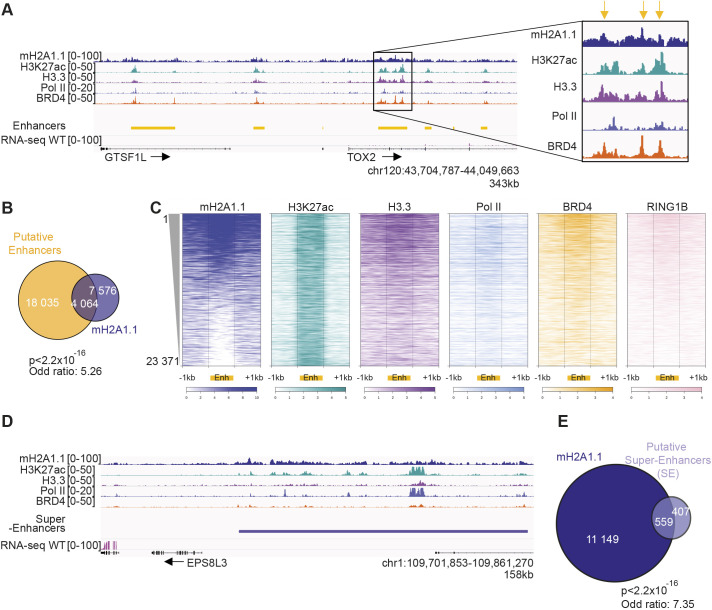
**mH2A1.1 associates with enhancers and SEs.** (A) Genome browser view of the indicated ChIP-seq illustrating occupancy of mH2A1.1 with ‘putative’ enhancers. The black box shows a magnification of one enhancer. Yellow arrows highlight the maximum signal of ChIP-seq data on this enhancer. (B) Overlap of ‘putative’ enhancers with mH2A1.1 peaks. Enrichment of mH2A1.1 peaks with enhancers were measured using Fisher exact tests. *P*-values and the odds ratios are shown. (C) Heatmap profiles showing the indicated ChIP-seq data relative enrichment around the enhancers (±1 kb). Color intensity reflects the level of ChIP-seq enrichment. Each line represents an enhancer (from 1 to 23,371 enhancers). Enhancers are ranked according to the level of mH2A1.1 on enhancers, as indicated. (D) Genome browser view of the indicated ChIP-seq illustrating occupancy of mH2A1.1 at ‘putative’ SEs. (E) Overlap of ‘putative’ SEs with mH2A1.1 peaks. Enrichment of mH2A1.1 peaks with SEs were measured using Fisher exact tests. *P*-values and the odds ratios are shown. ‘Putative’ enhancers and SEs are based on H3K27ac signal outside promoter regions using the ROSE package (Blinka et al., 2017).

In addition to promoters, mH2A1.1 also associates with intergenic regions (Fig. 1E). At the genome-wide level, mH2A1.1 binding strongly correlated with H3K4me1 (PCC of 0.55) and to a lesser extent with H3K27ac (PCC of 0.18), two chromatin marks that characterize enhancer regions (Creyghton et al., 2010) (Fig. 1D). In agreement, we found that mH2A1.1 binding was significantly enriched at enhancers (Fisher exact test: *P*<2.2×10^−16^ and odds ratio=5.26) (Fig. 5A,B). Enhancers bound by mH2A1.1 were further characterized by strong association of H3.3, Pol II, BRD4 and RING1B, which are marks of active enhancers (Chan et al., 2018; Chen et al., 2013; Lee et al., 2017) (Fig. 5C, Fig. S5A). Strikingly, mH2A1.1-bound regions frequently formed large domains comprising a group of enhancers marked with H3K27ac (Fig. 5D), which correspond to super-enhancers (SEs) (Lovén et al., 2013; Whyte et al., 2013) (Fisher exact test: *P*<2.2×10^−16^ and odds ratio=7.35) (Fig. 5E). Overall, these results show that mH2A1.1 binds enhancers and SEs in association with BRD4 and RING1B.

### mH2A1.1-target gene regulation does not require changes in enhancer-promoter looping

**Fig. 6.**
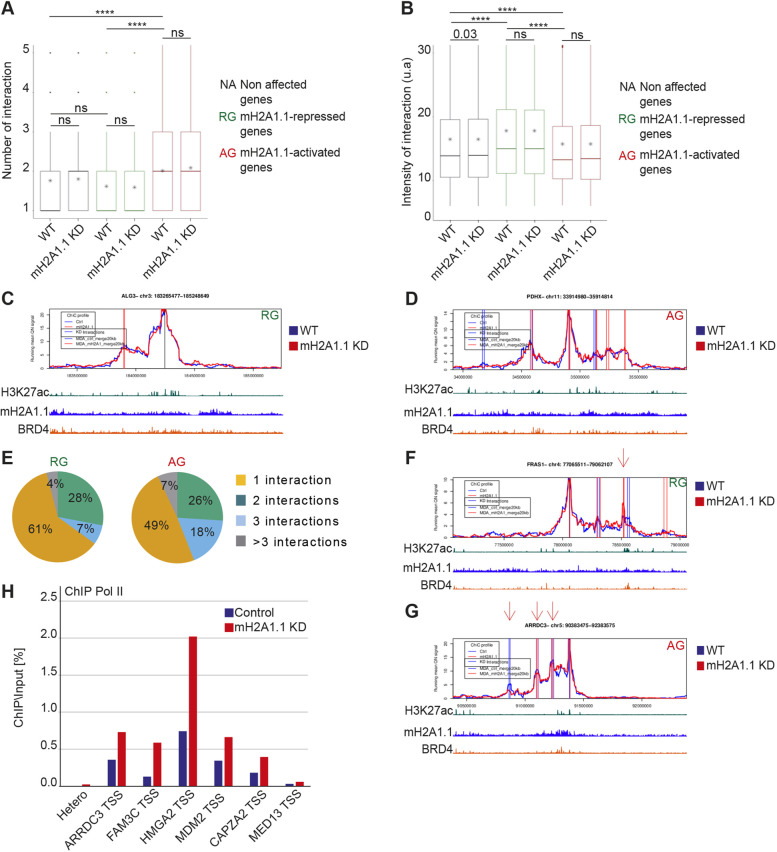
**mH2A1.1 regulates gene expression within predefined 3D chromatin domains.** (A) Boxplot showing the average number of PCHiC significant interactions per gene with adjacent genomic regions between genes not affected by mH2A1.1, mH2A1.1-repressed genes (*n*=181) and mH2A1.1-activated genes (*n*=282) in control and mH2A1.1 KD conditions. PCHiC significant interactions were determined using ChiCMaxima (Ben Zouari et al., 2019). The box represents the 25–75th percentiles, and the median is indicated (middle line). Whiskers extend to 25th percentile minus 1.5× IQR and 75th percentile plus 1.5× IQR. * represents the mean. Paired Wilcoxon tests were used to compare control and mH2A1.1 KD conditions whereas unpaired Wilcoxon tests were used to compare gene categories. ns, not significant. ****P<2.2×10^−16^. (B) Boxplot showing the mean of intensity of PCHiC interactions per gene between genes not affected by mH2A1.1, mH2A1.1-repressed genes (*n*=181) and mH2A1.1-activated genes (*n*=282) in control and mH2A1.1 KD conditions. Features of the boxplot and statistical tests as for A. (C,D) Snapshot of PCHiC dataset on the mH2A1.1-repressed *ALG3* gene (C) and the mH2A1.1-activated *PDHX* gene (D) in control and mH2A1.1 KD conditions. Interaction intensity between the target gene and the associated genomic region are plotted over a 2 Mb gene domain around the promoter bait. Control (blue line) and mH2A1.1 KD (red line) are shown. The vertical bars correspond to PCHiC significant interactions conserved between the two biological replicates in each condition. (E) Pie charts showing the percentage of mH2A1.1-target genes having one, two, three or more than three PCHiC significant interactions. (F) As in C, but this mH2A1.1-repressed gene, *FRAS1*, shows a reproducible gain of interaction with a specific genomic region (red arrow). (G) As in D, but this mH2A1.1-activated gene, *ARRDC3*, shows a reproducible reduction of interaction with specific genomic regions (red arrows). (H) ChIP-qPCR of Pol II on WT and mH2A1.1-depleted cells on six genes that show significative loss of interactions with adjacent genomic regions. Snapshots of PCHiC data set are shown in Fig. S6C. ‘Hetero’ corresponds to a negative position. For each gene, Pol II enrichment was evaluated only on the TSS. Results from additional biological replicates are given in Fig. S4E. For the snapshots of PCHiC data, only results from replicate number 1 are shown here; see Fig. S6A,B for replicate number 2.

Because mH2A1.1 binds enhancer and promoter regions (Figs 3 and 5), an attractive hypothesis was that mH2A1.1 mediates chromatin folding. To test this hypothesis, we applied promoter capture HiC (PCHiC) using a collection of 19,225 promoter sequence fragments as bait (Schoenfelder et al., 2018) in WT and mH2A1.1 KD cells. Genomic interactions between promoters and other genomic regions were called by ChiCMaxima (Ben Zouari et al., 2019). We aggregated the total number of detected interactions per gene for mH2A1.1-activated, -repressed or -independent genes. For each category, the average number of interactions detected per gene was identical in control and mH2A1.1 KD cells (Fig. 6A). The average intensity of interactions with adjacent genomic regions (±1.5 Mb around the gene) (Fig. 6B-D) or of interactions with enhancer regions (Fig. S5B) were generally unaffected by the absence of mH2A1.1. Hence, chromatin interaction landscapes at mH2A1.1-regulated genes do not appear to require mH2A1.1. Quantification of the PCHiC interactions showed that mH2A1.1-AGs have on average a greater number of interactions than mH2A1.1-RGs (Fig. 6A,E). However, interactions at mH2A1.1-AGs were weaker (Fig. 6B, Fig. S5B), suggesting that AGs and RGs reside within two types of interaction landscapes. Moreover, we noted that mH2A1.1 enrichment was greater at enhancers associated with RGs than at those associated with AGs (Fig. S5C,D). Active chromatin marks and co-activators were also more abundant at RG-related enhancers than AG-related ones (H3.3, Pol II, BRD4) (Fig. S5C,D), an observation in agreement with the fact that RGs are on average more transcribed than AGs (Fig. 1B,C).

Although loss of mH2A1.1 did not induce any global changes in promoter contact numbers or frequencies, closer inspection of the interaction landscape of a few mH2A1.1-regulated genes revealed reproducible changes in the intensity of interactions between certain enhancers in mH2A1.1 KD versus WT cells (Fig. 6F,G, Fig. S6B,C). For example, we observed an increase in the intensity of some interactions at the RG *FRAS1* upon mH2A1.1 depletion (Fig. 6F), and a decrease in the intensity of interactions at the AG *ARRDC3* (Fig. 6G), but this trend could not be generalized. Indeed, we also observed a decrease in the intensity of some interactions at RGs and an increase in the intensity of interactions at AGs (data not shown). Thus, gain or loss of interactions do not appear correlated to transcriptional changes upon loss of mH2A1.1. Furthermore, association of Pol II with the TSS increased upon mH2A1.1 depletion equally at some AGs, which are characterized by loss of interactions (Fig. 6H, Fig. S4E), and at other AGs for which 3D organization remained unaffected by mH2A1.1 depletion (Fig. 4E, Fig. S6D). We conclude that chromatin looping does not interfere with the potential role of mH2A1.1 in Pol II release.

### mH2A1.1 inhibits cell migration by activating the expression of paused genes regulating cytoskeleton organization

**Fig. 7.**
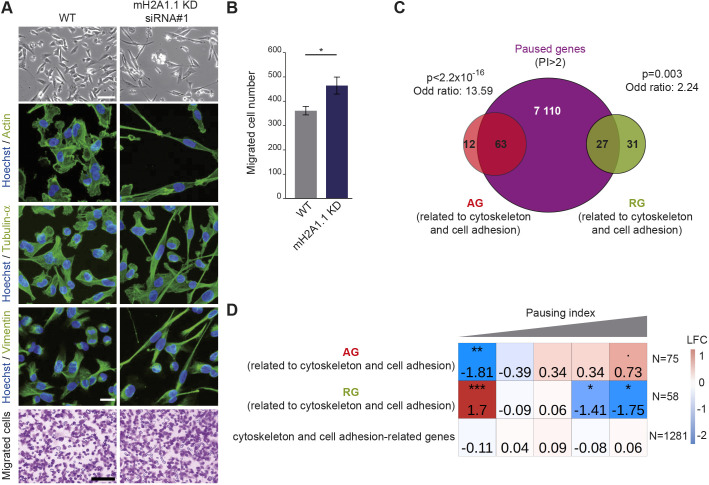
**mH2A1.1 inhibits cell migration by favoring expression of paused genes involved in cytoskeleton and cell adhesion in MDA-MB-231 cells.** (A) Top: Representative differential interference contrast microscopy images of WT and mH2A1.1 KD MDA-MB-231 cells. Scale bar: 100 µm. Center: Immunofluorescence labeling of actin, tubulin-α and vimentin. Nuclei are stained with Hoechst 33342. Scale bar: 20 µm. Bottom: Representative images of cells during the Boyden chamber migration assay. Only migrated cells are labeled in purple. Scale bar: 200 µm. (B) Quantification of the Boyden chamber assay presented in A. Error bars represent s.d from *n*=3 independent biological experiments as illustrated in A. Wilcoxon tests were used to compare conditions. *P<0.05. (C) Overlap of paused genes (*n*=7208) with mH2A1.1-regulated genes related to cytoskeleton and cell adhesion. Enrichment of this subgroup of mH2A1.1-regulated genes with paused genes was measured with Fisher exact tests; *P*-values and the odds ratios are shown. (D) Fisher test heatmap showing enrichment of indicated mH2A1.1-target genes with genes divided in five equal-sized categories as a function of their PI. Asterisks indicate the significatively of the Fisher exact tests; color map and values present in each square highlight the log2 odds ratio (LOR) of the Fisher exact test. *N* indicates the number of genes used for the analysis.

mH2A1.1-target genes are involved in four main processes: cell cycle (9% of mH2A1.1-target genes), DNA repair (4%), cytoskeleton organization and cell adhesion (19%) (Tables S6 and S7). The two first processes were expected based on earlier studies (Kim et al., 2018; Novikov et al., 2011; Sporn and Jung, 2012; Xu et al., 2012). A role for mH2A1.1 in the control of cell migratory capacities was previously identified in gastric cancer cells (Li et al., 2016), MDA-MB-231 cells (Dardenne et al., 2012) and mouse cell lines (Dardenne et al., 2012; Marjanović et al., 2018). However, the transcriptional impact of mH2A1.1 on cytoskeleton organization and cell adhesion genes is poorly documented except for recent work by Marcus Buschbeck and colleagues in murine C2C12 cells (Hurtado-Bagès et al., 2020). Two or three days following transfection of two different siRNA against mH2A1.1, MDA-MB-231 cells became more elongated (Fig. 7A, Fig. S7A). Using immunofluorescence against cytoskeleton proteins (actin, tubulin-α, vimentin), we observed that cytoskeleton organization was modified by the loss of mH2A1.1 (Fig. 7A). Moreover, numerous mH2A1.1-regulated genes are involved in cell migration, in particular *ARRDC3*, *SOCS4*, *HACE1* and *FBXL4*, which are mH2A1.1-AGs described as anti-migratory (Castillo-Lluva et al., 2013; Draheim et al., 2010; Mei et al., 2015; Stankiewicz et al., 2017), and *MMP14*, *EIF6*, *MT1E*, *JUND* and *DAPK3*, which are mH2A1.1-RGs with pro-migratory properties (Cathcart et al., 2015; Kake et al., 2017; Pinzaglia et al., 2015; Ryu et al., 2012; Selvaraj et al., 2015). In agreement, upon depletion of mH2A1.1, the migratory capacity of MDA-MB-231 cells was significantly increased compared with control cells (Fig. 7A,B). The effect of knocking down the other isoform, mH2A1.2, was opposite to that of mH2A1.1 (Fig. S7A-D). It would hence be interesting to determine whether the molecular mechanism by which the mH2A1.2 splicing isoform modulates transcription regulation, as well as the interplay between both isoforms.

Strikingly, mH2A1.1-AGs involved in cytoskeleton organization and cell adhesion were also amongst genes with a high Pol II PI compared with mH2A1.1-RGs (Fig. 7C,D), and compared with cell cycle and DNA repair mH2A1.1-AGs (Fig. S7E). Overall, we conclude that mH2A1.1 impedes the migration capacity of MDA-MB-231 breast cancer cells in part by promoting expression of genes modulating cell migration capacity.

## DISCUSSION

Regulating gene expression in a particular cell type requires fine-tuning of the transcriptional response. The concentration and the relative ratio between factors required for these regulatory mechanisms ensure rapid adjustments to maintain homeostasis or to respond to stimuli and stress. In this study, we identify the histone variant mH2A1.1 as a means to operate these adjustments in TNBC cells.

We present the first genome-wide map of endogenous histone variant mH2A1.1 in human breast cancer cells. We discovered that the mH2A1.1 variant specifically associates with transcription regulatory elements, promoters and enhancers, in addition to large domains of facultative heterochromatin. At promoters, we detected peaks of mH2A1.1 as opposed to the larger signals occurring within heterochromatin (Fig. 2B, Fig. S3A-E) (Chen et al., 2014; Douet et al., 2017; Gamble et al., 2010; Lavigne et al., 2015). Moreover, we found that selective depletion of the mH2A1.1 isoform was sufficient to modify expression of hundreds of actively transcribed genes in the MDA-MB-231 TNBC cell line (Fig. 1A-C). All of these genes are highly expressed in this cell line. We uncovered two distinct mechanisms through which mH2A1.1 regulates their transcription and link them to the chromatin landscape in which the affected genes reside.

**Fig. 8.**
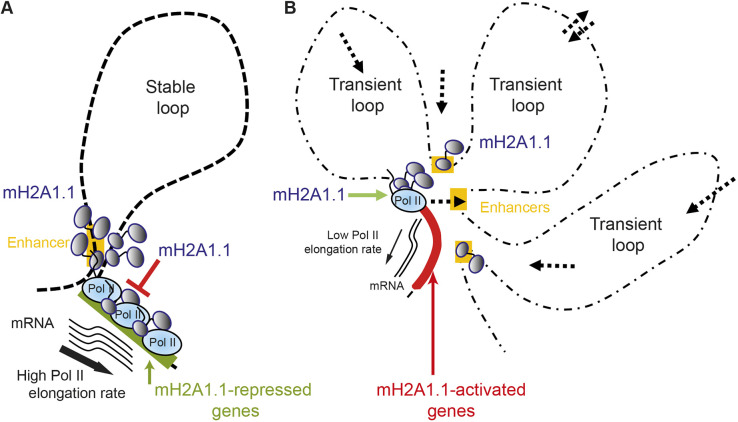
**Schematic of the two molecular mechanisms by which mH2A1.1 regulates transcription.** (A) mH2A1.1-repressed genes display a small number of stable interactions with enhancers or adjacent genomic regions characterized by bivalent chromatin marks. Pol II is enriched at the TSS and the gene body in this group of genes with a high Pol II elongation rate. The presence of mH2A1.1 all along the gene and associated enhancers slows Pol II elongation, perhaps by favoring recruitment of repressors. (B) mH2A1.1-activated genes display a large number of transient interactions. Some of them are established with enhancers bound by BRD4 and possess a specific chromatin landscape. Pol II is mainly paused in this group of genes, with a reduced Pol II elongation rate. Transient interactions between enhancers and promoters may promote Pol II pausing release, favored by mH2A1.1.

The first mechanism consists of dampening the transcription of highly expressed genes. Indeed, in the absence of mH2A1.1, these mH2A1.1-RGs were overexpressed. mH2A1.1 bound the gene bodies alongside Pol II, as well as their associated enhancers (Fig. 3, Fig. S5C,D). Importantly, these domains were also characterized by the presence of RING1B and the Polycomb-induced histone modification H2AK119ub (Chan et al., 2018) on their enhancers and promoters (Fig. S5C,D and data not shown). With the identification of specific PRC1 variants at a large number of active loci, it is now accepted that the presence of Polycomb subunits does not always correlate with transcriptional repression states (Giner-Laguarda and Vidal, 2020), especially in cancer cells (Chan et al., 2018; Zhang et al., 2020). Specifically, RING1B-target genes in MDA-MB-231 cells are transcriptionally active and highly expressed (Chan et al., 2018), just like some mH2A1.1-target genes studied here (Fig. 1B,C). We postulate that the presence of mH2A1.1 may favor binding of the PRC1 complex to regulate expression of mH2A1.1-RGs. Indeed, expression levels at mH2A1.1-RGs are only slightly increased in mH2A1.1-depleted MDA-MB-231 cells, similar to observations in a human lymphoma cell line (Lavigne et al., 2015), suggesting that mH2A1.1 may limit transcriptional noise and serve as a brake (Fig. 8A).

The second mechanism is specific to genes at which Pol II is paused. Here, in contrast to the RGs, mH2A1.1 recruitment is restricted to the TSS. Deletion of mH2A1.1 decreased the transcriptional level of these genes (mH2A1.1-AGs) and led to an accumulation of Pol II at their TSSs (Figs 1A-C, 4E, 6H). It is hence tempting to propose that mH2A1.1 assists the conversion of promoter-locked Pol II into a productive and elongating Pol II. The presence of BRD4 at the TSS of mH2A1.1-AGs likely favors transcription elongation by playing a role in allosteric activation of the P-TEFb complex (Winter et al., 2017). Thus, we can imagine that mH2A1.1 helps to recruit P-TEFb to promoter proximal regions and thereby contributes to the Pol II pause-release. Accumulation of Pol II at the TSS could also be due to accumulation of torsional stress, through topoisomerase inhibition (Teves and Henikoff, 2014). BRD4 is known to overcome transcription-induced torsional constraint by stimulating TOP1 activation concomitant with pause-release events (Baranello et al., 2016). Deletion of mH2A1.1 could impair this process, resulting in persistent torsional stress, accumulation of Pol II and inhibition of transcription. Finally, the chromatin organization at mH2A1.1-AGs appears to be more dynamic than that at mH2A1.1-RGs (Fig. 6). In these domains, more frequent but weaker contacts were detected by PCHiC. Pol II release could be facilitated by transient TSS-enhancer contacts in search for co-activators and Pol II (Fig. 8B). All paused genes in the MDA-MB-231 cell line exhibited this feature (data not shown). Dynamic 3D chromatin organization emerges as a new characteristic of paused genes. Conversely, the 3D organization of the mH2A1.1-RG loci appeared to be relatively stable, reminiscent of a productive and steady state environment for transcription (Figs 6, 8A,B).

The fact that mH2A1.1 binds specifically to the TSS of genes it activates in MDA-MB-231 cells is particularly intriguing here. The only other study that included an analysis of the genomic localization of this histone variant was performed in the murine muscle cell line C2C12 (Hurtado-Bagès et al., 2020). In this cell line, mH2A1.1 recruitment occurred upstream of the TSS of these genes, but also, in contrast to AG in MDA-MB-231 cells, over the gene body. Analysis of the genomic distribution of other macro histone variants shows cell type-specific patterns: Lavigne et al. reported binding of the mH2A1.2 isoform specifically to the TSS of mH2A1-regulated genes in two cancer cell types, HeLa and Namalwa cells (Lavigne et al., 2015). Comparison of genomic sites bound by mH2A1.2 nucleosomes revealed only a small overlap between HeLa and Namalwa cells. Similarly, mH2A1 and mH2A2 association with the TSS has also been observed in human and mouse embryonic stem cells (Pliatska et al., 2018; Yildirim et al., 2014). Therefore, we propose that mH2A variants are differentially recruited to regulatory sites depending on the carcinogenic and differentiation state of the cells. In breast cancers, recruitment, and thus the roles of, mH2A1 variants must be subtype specific. The newly identified binding pattern of mH2A1.1 we report here would thus be TNBC specific, because it was not identified in luminal breast cancer cell lines (Gamble et al., 2010). It could then explain why we found a correlation between mH2A1.1 expression levels and survival rates only in TNBC patients (Lavigne et al., 2014).

We further demonstrate that mH2A1.1-bound chromatin colocalizes with the H3K9me3 histone mark (Fig. S3A). A fraction of these sites is devoid of H3K27me3 and could correspond to the identified mH2A localization at constitutive heterochromatin (Douet et al., 2017). However, the vast majority of mH2A1.1-bound H3K9me3-decorated chromatin also contained tri-methylated H3K27 (Fig. S3A-E). This difference may be a feature of the MDA-MB-231 cell line, a high migratory capacity cancer cell line in which H3K9me3 histone marks are distributed unusually (Segal et al., 2018; Yokoyama et al., 2013). Overall, heterochromatin-related processes in this cancerous cell appear modified compared with non-cancerous cells and could potentially result from or favor malignant cellular transformation (Segal et al., 2018; Yokoyama et al., 2013). Thus, it could be interesting to investigate additional molecular mechanisms, enzymes and epigenetic machineries that are altered in this cancer type.

Despite the association of mH2A1.1 with heterochromatin, its phenotypic knockdown was not sufficient to reactivate silenced genes present in these domains (Fig. 1B,C, Fig. S3A-E). Different hypotheses could explain this result. The first hypothesis could be that mH2A1 isoforms (mH2A1.1 and mH2A1.2) have redundant actions at heterochromatin. Here, we specifically depleted mH2A1.1 without affecting the expression of mH2A1.2 (Fig. S1D). The presence of mH2A1.2 could be sufficient to maintain gene silencing, although mH2A1.2-occupied silent genes were also not reactivated upon mH2A1.2 knockdown (Dell'Orso et al., 2016). However, even if mH2A1 binding was shown to overlap with H3K27me3-decorated chromatin in primary human cells, no enrichment of H3K27me3 at mH2A1-regulated genes (both isoforms) was observed (Chen et al., 2014). The second hypothesis could be that mH2A1.1 and mH2A1.2 may both serve as a lock to conserve heterochromatin stability/organization but are not required for gene silencing. In agreement with this hypothesis, two studies demonstrated that mH2A are implicated in the condensation of heterochromatin regions, such as lamin-associated-domains and repeated DNA elements, without drastically affecting their expression level (Douet et al., 2017; Fu et al., 2015). Further analyses are needed to better define the role of mH2A at heterochromatin regions.

In this study, we demonstrate that a key function of mH2A1.1 is to orchestrate proper transcriptional output of genes depending on their environment, yet mH2A1.1 did not seem necessary for chromatin topologies. We cannot exclude the possibility that mH2A1.1 participates in short-range or transient structural changes that our approach is not sensitive enough to identify. However, others have reported that key transcription factors or co-factors do not alter 3D folding and, in particular, enhancer-promoter looping (Schoenfelder and Fraser, 2019). For example, in MDA-MB-231 cells, the FRA1 activator binds to promoters and enhancers, but does not mediate looping (Bejjani et al., 2021). In SEM leukemia cell lines, BRD4 inhibition has only minor effects on enhancer-promoter interactions (Crump et al., 2021), despite a strong effect on key oncogenic target gene expression. Thus, stabilization of enhancer-promoter loops is not always a prerequisite for transcriptional fine-tuning by transcriptional regulators. We could speculate that their roles are more functional by facilitating interactions between enhancer-associated factors, as was observed for BRD4 through the formation of phase-separated condensates (Sabari et al., 2018). It would be interesting to test whether mH2A1.1 participates in this process, especially as we have identified a preferential association of mH2A1.1 with SEs (Fig. 5D,E). SEs are known to play an important part in many diseases, including several cancers in which they drive expression of oncogenes (Donati et al., 2018; Lovén et al., 2013), and the expression of mH2A1.1 is altered in cancer cells compared with normal tissues (Cantariño et al., 2013; Lavigne et al., 2014; Sporn and Jung, 2012). SE function may be compromised by variations in mH2A1.1 levels leading to the inability to fine-tune transcriptional output, in particular via the first mechanism described above (Fig. 8), which is necessary to avoid excessive transcription of oncogenes.

## MATERIALS AND METHODS

### Cell culture

MDA-MB-231, HEK-293T and MCF7 cell lines were purchased from ATCC, and were maintained and amplified in Dulbecco's Modified Eagle's (DMEM) for HEK-293T and MDA-MB-231 cells, and in DMEM-F12 for MCF7 cells, supplemented with gentamycin (50 µg/ml) (Gibco), fetal bovine serum (FBS; 10%, Gibco) and sodium pyruvate (100 mM, Sigma-Aldrich). Cells were maintained in a humidified incubator at 37°C with 5% CO_2_. Cells lines were regularly tested for *Mycoplasma* infection (MycoAlert, Lonza). In Montpellier, MDA-MB-231 cells were cultured in DMEM supplemented with 10% fetal calf serum and penicillin/streptomycin (100 µg/ml each) and regularly tested for *Mycoplasma* infection.

### Transfection of siRNAs and plasmids

At 30-50% confluence, transfection of siRNA (11 nM) was performed using INTERFERin (Polyplus-Ozyme) according to the manufacturer's protocol. Cells for the control condition were transfected with INTERFERin without any siRNA. Transfection of plasmid (1 μg) was carried out with FuGene HD (Promega) according to the manufacturer's protocol. siRNA and plasmids are listed in Table S8. Cells were recovered for experiments 2 and 3 days after plasmid and siRNA transfection, respectively.

### Western blotting

Cells were lysed and subjected to western blot analysis as previously described (Yang et al., 2014). Briefly, proteins extracts were separated in 10% polyacrylamide (1:125 bisacrylamide:acrylamide) SDS gels, transferred onto nitrocellulose membrane (Bio-Rad) and blocked with 0.4% Tween 20, 5% milk in PBS for 1 h at room temperature (RT) with rotation. Membranes were then incubated with primary antibodies overnight (O/N) at 4°C in 0.4% Tween 20, 5% milk in PBS with rotation (or 90 min at RT). Primary antibodies are described in Table S4. Rabbit anti-mH2A1.1 antibody was generated according to an immunization protocol from Agro-Bio (La Ferte Saint Aubin, France). Membranes were next incubated with secondary antibody 0.4% Tween 20, 5% milk in PBS for 1 h at RT with rotation and the signal was detected using chemiluminescence (Lumi-light^plus^ western blotting substrate; Roche). Secondary antibodies are described in Table S4. Signal quantifications were carried out with Image Lab software (Bio-Rad).

### RNA extraction, reverse transcription and quantitative real-PCR

Total RNA was isolated using the RNeasy Midi Kit (QIAGEN). Purified RNA was reversed transcribed to cDNA using Maxima H Minus First Strand cDNA Synthesis Kit (Promega). The sequences of the primers used are given in Table S9. qRT-PCR was performed using iTAq Universal SYBR Green (Bio-Rad) according to the manufacturer's instructions. At least two independent experiments were performed for each condition. The relative expression levels of mRNA were normalized to *RPLP0* mRNA expression and evaluated according to the 2^−ΔΔCt^ method (Rao et al., 2013).

### Fluorescence microscopy

Two- or three-days post-transfection, cells were fixed with 4% paraformaldehyde for 15 min for MDA-MB-231 cells and 10 min for HEK-293T at RT. Cell permeabilization was carried out using 0.1% Triton X-100 in PBS for 10 min at RT. Cells were then blocked with 5% BSA, 0.15% Tween 20 in PBS for 1 h at RT. Next, cells were incubated with primary antibody O/N at 4°C. Cells were then incubated with Alexa Fluor-conjugated secondary antibody for 1 h at RT. Actin was labeled using cytoPainter Phalloidin iFluor diluted 1:1000 with secondary antibody according to the manufacturer's protocol (Abcam, Ab176759). Antibody references and dilutions are provided in Table S4. The coverslips were finally incubated with Hoechst (Invitrogen, 33342) for 30 min and then mounted with VECTASHIELD mounting media (Vector Laboratories). Images were acquired with a Zeiss LSM 710 BiG confocal microscope using an 63× PL APO oil DIC On 1.4 objective for all experiments. Images were taken in *z*-stacks with a voxel size of 300 nm. Images shown are *z*-stacks or maximum intensity projections of *z*-stacks.

### ChIP and library preparation

Cells were cross-linked in DMEM containing 1.2% paraformaldehyde at RT for 10 min with rotation. Cross-linking was stopped by the addition of glycine to a final concentration of 0.125 M for 5 min. Cell were harvested and lysed in cell lysis buffer (10 mM Tris-HCl, pH 7.4, 15 mM NaCl, 60 mM KCl, 1 mM EDTA, 0.1 mM EGTA, 0.2% NP-40, 5% sucrose). After 10 min in ice, cell lysis was amplified with a 2 ml dounce (Kimble Chase) to enhance the separation of nuclei from cytoplasm. Cell lysis buffer containing lysed cells was deposited into a pillow buffer (10 mM Tris-HCl, pH 7.4, 15 mM NaCl, 60 mM KCl, 1 mM EDTA, 0.1 mM EGTA, 0.2% NP-40, 10% sucrose). Nuclei were then pelleted by centrifugation (845 ***g*** for 20 min) and washed with washing buffer (10 mM Tris-HCl, pH 7.4, 15 mM NaCl, 60 mM KCl). Nuclei were then resuspended in sonication buffer [50 mM Tris-HCl, pH 7.5, 150 mM KCl, 5 mM EDTA, 1% NP-40, 0.1% SDS, 0.5% sodium deoxycholate, protease inhibitor (Roche)]. Chromatin was sheared using a Bioruptor (Diagenode) (30 cycles, 30 s ON/30 s OFF) in order to obtain chromatin fragments with an average size of 300-500 bp. The quality and size of chromatin fragments was monitored by ethidium bromide-stained agarose gel electrophoresis after DNA purification. Then, 100 μg of DNA was incubated with antibody O/N at 4°C on a rotation wheel. Antibodies are described in Table S4. Protein A magnetic dynabeads (3 mg; Sigma-Aldrich) were added for 3 h at 4°C on a rotation wheel. Immunoprecipitates were then exposed to serial washes for 5 min each on a rotation wheel at 4°C in the following buffers (twice in each buffer): WB_I_: 2 mM EDTA, 20 mM Tris, pH 8.1, 1% Triton X-100, 150 mM NaCl; WB_II_: 2 mM EDTA, 20 mM Tris, pH 8.1, 1% Triton X-100, 500 mM NaCl; WB_III_: 1 mM EDTA, 10 mM Tris, pH 8.1, 250 mM LiCl, 1% sodium deoxycholate, 1% NP-40; and WB_IV_: 1 M EDTA, 10 mM Tris, pH 8.1. Chromatin was eluted from the magnetic beads with DNA isolation buffer (2% SDS, 0.1 M NaHCO_3_) for 1 h at 65°C under agitation. Extracts were reverse-crosslinked with SDS O/N at 65°C. RNAs were degraded with RNase A and proteins were finally degraded with proteinase K. The same procedure was performed for input (10 μg of DNA). DNA was finally extracted with a phenol-chloroform extraction. Quantity and quality of DNA was tested with a nanodrop spectrophotometer (NanoDrop 2000, Thermo Fisher Scientific). Samples were sequenced by the GeT core facility, Toulouse, France (http://get.genotoul.fr). Sequencing was performed using a HiSeq 3000-HWI-J00115 (Illumina) according to the manufacturer's protocol. The same procedure was used for ChIP-qPCR. Sequences of the primers used for qPCR are given in Table S9. For western blot analysis, extracts [input (10% IP), no immunoprecipitated (NoIP) fraction and IP fraction] were processed as for ChIP extracts, but were not incubated with RNase A and proteinase K. Extracts were then subjected to western blot analysis as previously described in the ‘Western blotting’ section. To compare different extracts, we loaded 2% input, 0.5% input, 0.5% NoIP fraction and 20% IP fraction. Percentages given are relative to the DNA quantity used for ChIP (Fig. 2C,D,G).

ChIP-seq of H3K27ac, H3K4me1, H3K4me3, H3K36me3 and Pol II was performed essentially as previously described (Tolza et al., 2019; Bejjani et al., 2021). Briefly, after cell fixation with 1% paraformaldehyde at RT for 5 min, cells were incubated in cell lysis buffer (5 mM PIPES, 85 mM KCl, 0.5% NP40, 10 mM sodium butyrate, protease inhibitors) for 10 min on ice. After mild centrifugation (845 ***g*** for 20 min), nuclei were lysed in Nuclei Lysis Buffer (50 mM Tris-HCl, pH 7.5, 0.125% SDS, 10 mM EDTA, 10 mM sodium butyrate, protease inhibitors) at 4°C for 2 h and then sonicated for ten cycles at 4°C using a Bioruptor Pico device from Diagenode. For immunoprecipitation of H3K4me1, H3K4me3 and H3K27ac, 150 µl of chromatin (equivalent to 4×10^6^ cells) and 4.5 µg of the corresponding antibodies were used. For Pol II, 850 µl of chromatin (equivalent to 22×10^6^ cells) and 20 µg of the corresponding antibody were used. Each ChIP run was sequenced by the MGX GenomiX platform (Montpellier) using a Hi-seq 2500 Illumina sequencer. ChIP-qPCR of Pol II was carried out following the same protocol as for Pol II ChIP-seq. qPCR was performed on ChIP samples and input (1% of DNA used for ChIP). qPCR results are normalized using the signal obtained with the input and expressed as percentage of input. Primers used are given in Table S9.

### Strand-specific total RNA library preparation

Total RNA was isolated using the RNeasy Midi Kit (QIAGEN). RNA-seq quality and quantity controls were performed using a Nanodrop spectrophotometer (NanoDrop 2000, Thermo Fisher Scientific) and a 5300 Fragment Analyzer system (Agilent). Library preparation and sequencing was carried out by the GeT core facility (Toulouse, France; http://get.genotoul.fr) with the TruSeq Stranded total RNA Kit (Illumina) according to the manufacturer's instructions. Sequencing was performed using a HiSeq 3000-HWI-J00115 according to the manufacturer's protocol.

### ChIP-seq data processing

The quality of the reads was estimated with FastQC (Illumina, 1.0.0). Published ChIP-seq data for H3K9me3 (GSM2258862), H3K27me3 (GSM2258850) and corresponding input (GSM2258864) in MDA-MB-231 cells were downloaded from Gene Expression Omnibus (GEO accession number: GSE85158) (Franco et al., 2018), and re-analyzed as described. Published ChIP-seq data for BRD4 (GSM2862187), RING1B (GSM2862179), PCGF2 (GSM2862185) and H2AK119ub (GSM2862181) in MDA-MB-231 cells were downloaded from Gene Expression Omnibus (GEO accession number: GSE107176) (Chan et al., 2018), and re-analyzed as described. Published ChIP-seq data for H3.3 (GSM3398219), and corresponding input (GSM3398220) in MDA-MB-231 cells were downloaded from Gene Expression Omnibus (GEO accession number: GSE120313) (Ben Zouari et al., 2019) and re-analyzed as described. Published ChIP-seq data for PARP1 (GSM1517306) in MDA-MB-231 cells were downloaded from Gene Expression Omnibus (GEO accession number: GSE61916) (Nalabothula et al., 2015) and re-analyzed as described. Sequenced reads were aligned to the human genome assembly GRCh38 using STAR (2.5.1) algorithm with default parameters (Dobin et al., 2013). Details are supplied in Table S5. Low-quality reads were then filtered out using Samtools (Samtools, options –q 10 -view) (Li et al., 2009). Conversion of BAM files to bigWig files was performed with the Bamcompare tool (DeepTools utilities v3.1.3) (Ram et al., 2016). Corresponding ChIP-seq data generated from genomic DNA (input) were used as control for every bigWig files normalization (options: --normalizeUsing RPKM --operation subtract --binSize 50 bp --smoothLength 150 bp). Peaks were determined with the enrichR function of NormR package (https://github.com/Your-Highness/NormR, R Package Version 1.8.0). NormR parameters were adjusted depending on the bigwig profiles for each ChIP-seq data. mH2A1.1-specific peaks were used for all analysis and correspond to the commun peaks between mH2A1.1 and mH2A1 ChIP-seq. The number of peaks for each ChIP-seq dataset are listed in Table S5. All downstream analyses were mainly performed with R studio. ChIP-seq signal and peaks positions visualization were obtained with IGV (Thorvaldsdóttir et al., 2013). Boxplots were created with ggplot2 (Wickham, 2016). Distributions of mH2A1 isoforms and H3K27me3/H3K9me3 common peaks identified at specific genomic features were calculated using the ChIPseeker package with default parameters (Fig. 1E, Fig. S3D) (Yu et al., 2015). Statistical analyses are described in the ‘Statistics and Reproducibility’ section.

### Identification of ‘putative’ enhancers and SEs

All putative enhancers were determined with ROSE utility tools based on H3K27ac signal outside the TSS (±2 kb) to avoid TSS bias (Fig. 5A-C) (Blinka et al., 2017). TSS annotation is based on TxDb.Hsapiens.UCSC.hg38.knownGene release (*n*=25,668 annotated genes). SEs were determined with ROSE utility tools based on H3K27ac signal (options: stitching_distance=12.5 kb and TSS_exclusion_zone_size: 2500 bp) (Fig. 5D,E) (Lovén et al., 2013; Whyte et al., 2013).

### Pol II PI calculation

PI was defined previously (Zhang et al., 2017) as the ratio of Pol II density in the promoter-proximal region [(−30;300) bp centered on the TSS] to the Pol II density in the transcribed regions (TSS+300 bp to the TES). Gene annotation is based on TxDb.Hsapiens.UCSC.hg38.knownGene release. Density of Pol II was calculated using the Pol II bigWig file, normalized using --log2 option (DeepTools utilities v3.1.3) (Ram et al., 2016), and the negative values were replaced by zeros. Genes with a width <1 kb were excluded from the analysis. Moreover, PI was not calculated for genes with a Pol II density <1.2 in the promoter-proximal region and a Pol II density in the transcribed regions <0. Using this threshold, we only calculated PI for transcribed genes with Pol II binding (*n*=10,564 genes). ‘Paused’ genes were defined as genes with a PI >2 (*n*=7208) (Day et al., 2016). ‘Not paused’ genes were defined as genes with a PI <2 (*n*=3356).

### Venn diagrams

Intersection of peaks was determined with the function findOverlaps() from the GenomicRanges package (Lawrence et al., 2013). Note that for two ChIP-seq peak intersections only the number of overlaps was counted and not the number of each peaks contained per overlap. This particularity explains why the number of peaks changes between Venn diagrams for the same ChIP-seq. The area-proportional Venn diagrams were drawn based on images generated by Vennerable package. Enrichment tests associated with Venn diagrams are explained in the ‘Statistics and reproducibility’ section.

### Correlation heatmaps

Correlation heatmaps using bigWig of indicated ChIP-seq were created with multiBigwigSummary (with or without the options: -bins) and plotCorrelation (option: -spearman correlation heatmap) from DeepTools utilities (3.1.3) (Ram et al., 2016).

### Fisher test heatmaps

Fisher test heatmaps were created using ggplot2 (Wickham, 2016). Each square on the heatmap shows the results of a Fisher exact test between the two groups tested. The positive or negative association between the two groups tested is established by the odds ratio, represented by the ‘score’ [log 2 (odds ratio)=LOR] and the color scale, which is proportional to the score. Significance of the overlap is indicated by the *P*-value, represented by asterisks (*P≤0.05; **P≤0.01; ***P≤0.001; ****P≤0.0001). Groups used for the analysis were divided in equal size according to the ChIP-seq signal.

### Metagene profiles

Metagene analysis profiles were performed with the R Seqplot package (Stempor and Ahringer, 2016) using bigWig files (functions getPlotSetArray and plotAverage) centered on the TSS (±2 kb) or from the TSS to the TES (±2 kb). Profiles correspond to the mean value (±s.e.m.). Values >2 s.d. were considered as outliers, were removed and were not used to generate the profiles. Heatmaps profiles were also created with the R Seqplot package using bigWig files (functions getPlotSetArray and plotHeatmap) (Stempor and Ahringer, 2016). Some heatmaps profiles were also ranked according to ChIP-seq signal, PI index or gene expression log2 fold change. On all heatmaps, color intensity reflects the level of ChIP-seq enrichment. Color intensity autoscales were always used excepted for the heatmaps shown in Fig. S5C to compare the relative enrichment between mH2A1.1-target genes and their associated enhancers. On profiles and heatmaps, gene directionality was ignored, meaning that all gene bodies are artificially placed on the right-hand side of the plots.

### bigWig signal quantification

bigWig signals of the indicated ChIP-seq data were calculated with R studio based on bigWig files. For all the figures, the sum bigWig file (bin of 50 bp) was calculated on specific genomic regions and normalized to the width of the specific genomic regions.

### RNA-seq analysis

The quality of the reads was estimated with FastQC (Illumina, 1.0.0). The reads were mapped to the human reference genome GRCh38 using the default parameters of STAR (2.5.1) (Dobin et al., 2013). Details are supplied in Table S5. Low-quality reads and duplicates were then filtered out using SAMtools (Samtools, options -q 10 –view ; -rmdup) (Li et al., 2009). Unstranded normalized bigwig files in reads per kilobase per million mapped reads (RPKM) were obtained with the bamCompare tool (DeepTools utilities v3.1.3) (options: --normalizeUsing RPKM --operation subtract --binSize 50 bp). (Ram et al., 2016). Gene counts were performed with htseq-count utilities with default parameters (0.8.0) (Stempor and Ahringer, 2016). FPKM for all genes was calculated with the formula: FPKM=(RC_g_×10^6^)/(RC_p_×L), where RC_g_ corresponds to the number of reads mapped to the gene, RC_p_ to the number of reads mapped to all protein-coding genes and L to the length of the gene in base pairs. FPKM gene counts in control conditions were used to classify genes according to their gene expression level in four equal-sized categories [silent (*n*=5625), low expression (*n*=5625), medium expression (*n*=5625) and high expression (*n*=5625)]. FPKM gene counts in the mH2A1.1 KD condition were also calculated and used to generate the graph in Fig. 1B. Differential expression analysis was performed with DESeq2 package (Love et al., 2014) with cutoff |FC|>1.5 and *P*adj<0.1. The corresponding volcano plot was created with the EnhancedVolcano package (https://github.com/kevinblighe/EnhancedVolcano, R package, 2018) (Fig. 1A). mH2A1.1 KD de-regulated genes are listed in Tables S1 and S2.

### PCHiC and library preparation

PCHiC data were generated for MDA-MB 231 cells in control and mH2A1.1 KD cells using siRNA#1 (see the ‘Transfection of siRNAs and plasmids’ section and Table S8). PCHiC was essentially performed as described by Schoenfelder et al. (2018), using nearly the same promoter library as Mifsud et al. (2015) (omitting probes from chromosomes 8, 9 and X).

### PCHiC analysis

ChiCMaxima_calling was performed using the same default parameters and merging of replicate results as Ben Zouari et al. (2019) after processing the fastq files with custom scripts, essentially performing the same analysis as HiCUP (Wingett et al., 2015). Comparison of the number of called interactions between subgroups was carried out for the data shown in Fig. 6A. Intensity of interactions were estimated based on PCHiC read counts for each biological replicate. Only reads >5 for each biological replicate were kept and interactions between a bait and the other-end closer than 1.5 Mbp. Finally, for each interaction, read counts were quantile normalized using the function ‘normalizeBetweenArrays’ from the limma package (Ritchie et al., 2015). Means between biological replicates were used. ChICMaxima Browser was used to generate PCHiC profiles (https://github.com/yousra291987/ChiCMaxima) (Ben Zouari et al., 2019). ChiCMaxima-called and merged interactions were overlapped with enhancers using the findOverlaps function from the R GenomicRanges package (Lawrence et al., 2013). More than one enhancer can significantly be in interaction with mH2A1.1-regulated genes. To simplify, only one enhancer per gene was conserved to generate the heatmaps in Fig. S5C. Some mH2A1.1-target genes are not present in those heatmaps because they do not have PCHiC-called interactions with an enhancer or did not have PCHiC capture oligonucleotides.

### Gene ontology analysis

GO analysis was performed with limma package (--function goana) (3.8) (Ritchie et al., 2015) and corresponding GO terms are supplied in Tables S6 and S7. A selection of genes related to their functions was identified with biomaRt package [function getBM()] (Durinck et al., 2005, 2009). For genes related to cytoskeleton (GO:0005856), cell adhesion (GO:0007155), cilium (GO:0005929) and cell junction (GO:0030054) (*n*=2509) using attributes=’ensemble gene_id’ annotation from the biomaRt package, overlaps of genes with mH2A1.1-regulated genes were identified [mH2A1.1-activated (*n*=87/533), mH2A1.1-repressed genes (*n*=71/412)]. For genes related to cell cycle (GO:0007049), cell cycle process (GO:0022402), cell division (GO:0051301) and cell growth (GO:0016049) (*n*=656), overlaps of genes with mH2A1.1-regulated genes were identified [mH2A1.1-activated (*n*=64/533), mH2A1.1-repressed genes (*n*=18/412)]. Finally, for genes related to DNA repair (GO:0006281) and cellular response to DNA damage stimulus (GO:0006974) (*n*=533), overlaps of genes with mH2A1.1-regulated genes were identified [mH2A1.1-activated (*n*=37/533), mH2A1.1-repressed genes (*n*=4/412)]. For Fisher test heatmaps with PI, only genes with a PI were used. *n* indicates the number of genes used for the analysis in Fig. 4B, Fig. 7D and Fig. S7E.

### Transwell migration assay

Transwell migration assays were performed using Transwell plates with 0.8 µm pore polycarbonate membranes (Corning Transwell, Sigma-Aldrich). Three days post-siRNA transfection, MDA-MB-231 cells were seeded in the upper chamber without FBS and allowed to invade to the reverse side of the chamber under chemoattractant conditions with 10% FBS-containing medium in the lower chamber. Following incubation for 16 h at 37°C, the cells were fixed with 3.7% formaldehyde for 2 min at RT. Cell permeabilization was carried out by incubation in 100% methanol for 20 min at RT. Cells were then stained with Giemsa for 15 min at RT. The final total cell number between conditions was always checked by wide-field microscopy to avoid proliferation bias for migratory cell comparison. Non-migrated cells were finally removed from the upper chamber using a cotton swab. Migrated cells adhering to the underside of the chamber were photographed using a light microscope at 200× magnification (Invitrogen EVOS Digital Color Fluorescence Microscope). Cell counting was performed with ImageJ in ten different fields per condition (Schneider et al., 2012). Three independent experiments were performed for each condition.

### Statistics and reproducibility

All western blot, qRT-PCR and Boyden chamber assay experiments were repeated at least twice as independent biological replicates and results are presented as mean±s.d. All statistical analyses were performed with R. For western blotting, qRT-PCR and Boyden chamber assays, Wilcoxon tests were used to compare mean values between conditions. *P*-values ≤0.05 were considered as significant or highly significant when ≤0.01 (given as *P≤0.05; **P≤0.01; ***P≤0.001; ****P≤0.0001. Fisher exact test was used to perform enrichment tests of ChIP-seq peaks. Base sets were defined from all the ChIP-seq data or based on TSS annotations.

## Supplementary Material

Supplementary information

Reviewer comments
