# Supplementary material for: The histone variant macroH2A1.1 regulates RNA polymerase II-paused genes within defined chromatin interaction landscapes
Source: J Cell Sci. 2022 Apr 11;135(7):jcs259456. doi: 10.1242/jcs.259456 (PMC9016624; doi:10.1242/jcs.259456)
Supplement: Supplementary information [file joces-135-259456-s1.pdf]

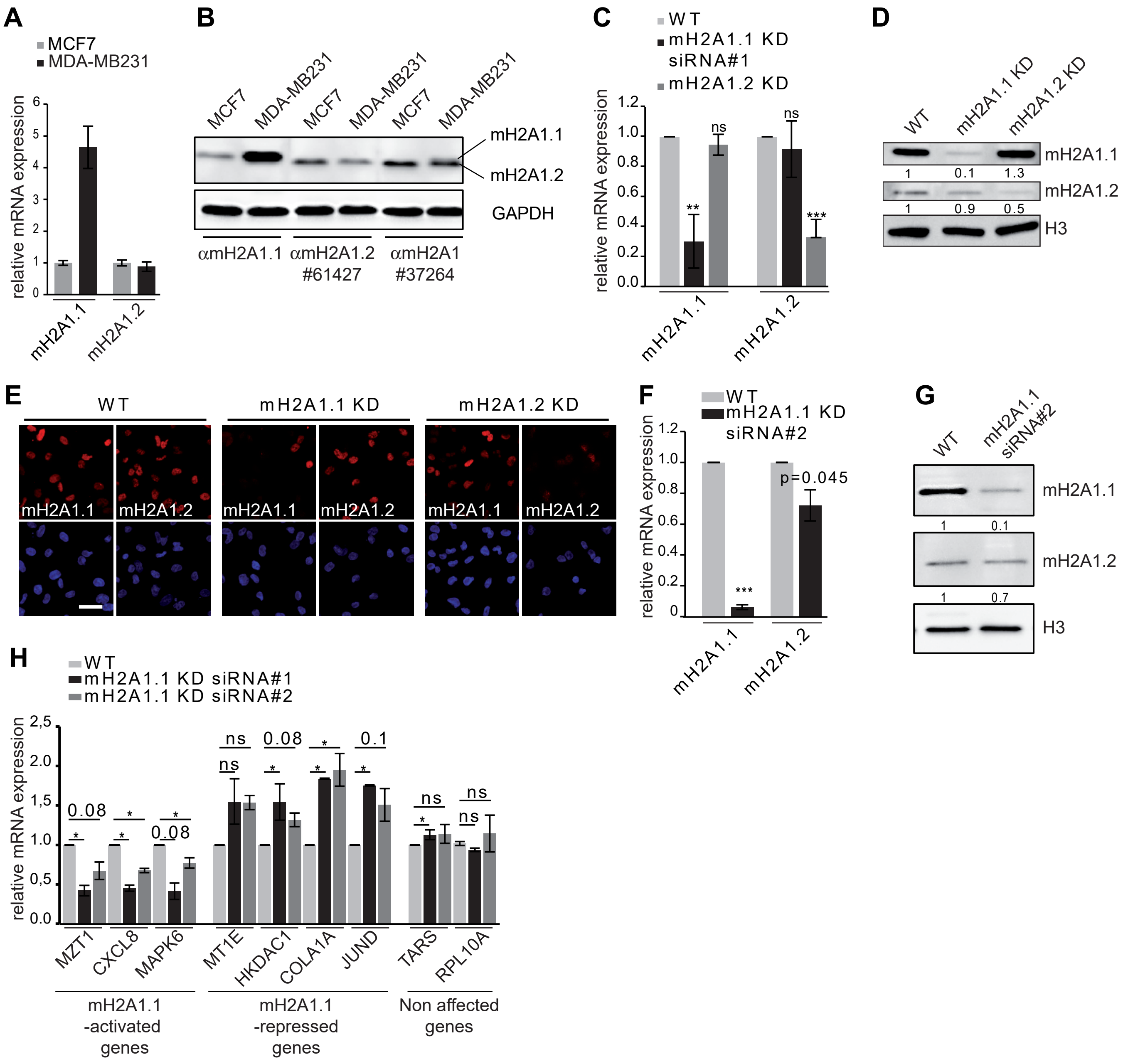

Fig. S1. RNAi knock down of specific mH2A1 isoforms in MDA-MB231 cells. (A) RTqPCR on MDA-MB231 and MCF7 cells showing expression levels of mH2A1 isoforms. Error bars represent s.d from independent biological experiments. (B) Western blot on whole cell extracts of MDA-MB231 and MCF7 cells showing protein levels of mH2A1 isoforms. GAPDH is used as a loading control. (C) RTqPCR quantifying KD of mH2A1 isoforms. (D) Western blot showing specific depletion of mH2A1 isoforms protein. H3 is used as a loading control. (E) Immunofluorescences showing specific partial depletion of mH2A1 isoforms. DNA is labelled with Hoechst. Scale bar = 20 µm. (F) As in (C) but with a second siRNA against mH2A1.1 (siRNA #2). (G) As in (D) but with a second siRNA against mH2A1.1 (siRNA #2). H3 is used as a loading control. (H) RTqPCR analysis of a subset of RNAseq-defined mH2A1.1 regulated-genes. Genes are divided in three groups, as indicated. Analysis were done three days post-transfection of specific siRNAs. RTqPCR, mRNA expressions are normalized by RPLP0 mRNA. Error bars represent s.d from independent biological experiments (n>=2). Student-tests were used to compare conditions. *: p-value < 0.05, ***: p-value < 0.001, ns, not significant. (D, G) Band quantifications are shown, normalized to protein loading control.

Journal of Cell Science • Supplementary information

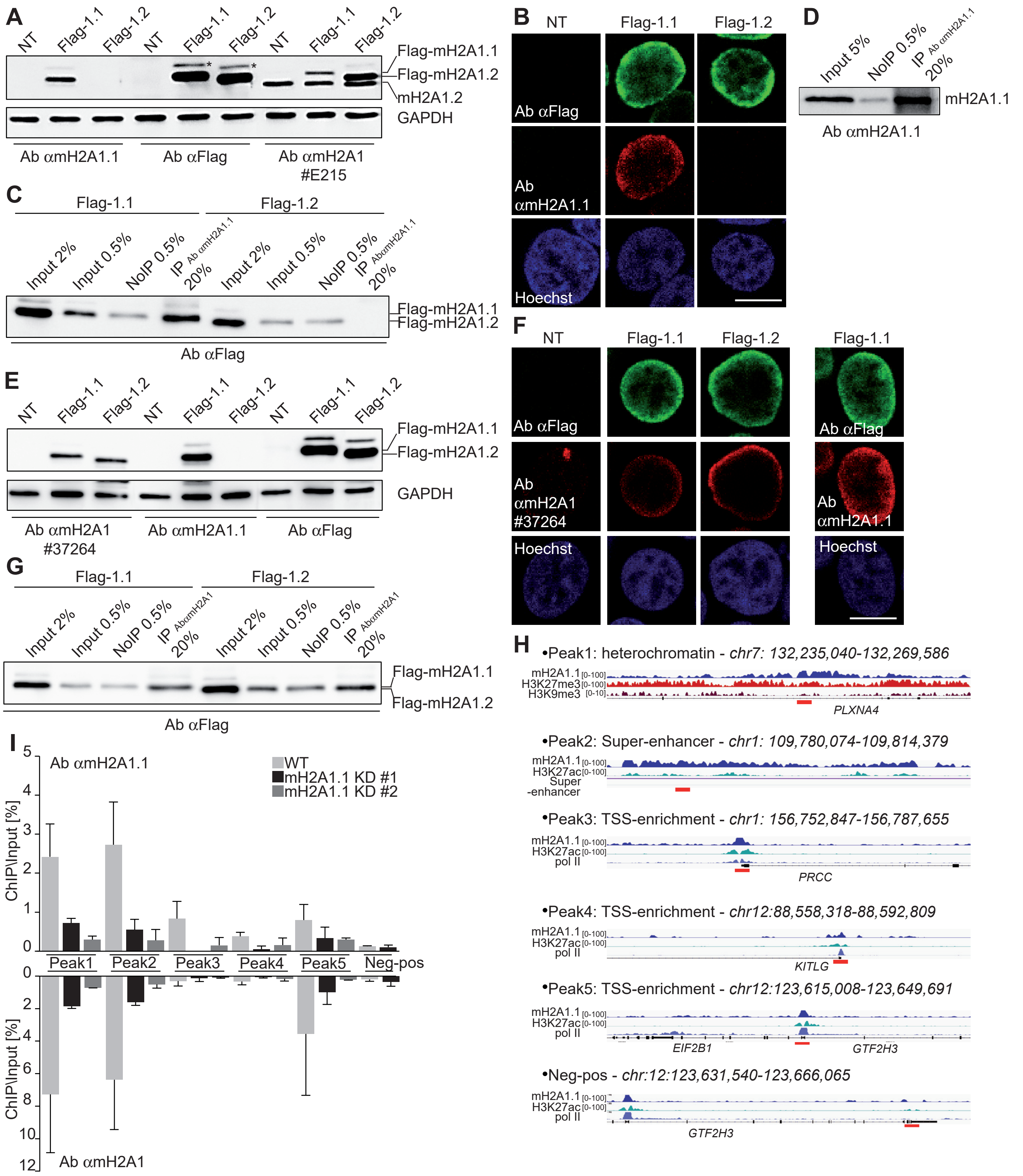

Journal of Cell Science • Supplementary information

Fig. S2. The antibody Ab αmH2A1.1 recognizes specifically the mH2A1.1 isoform. (A) Western blot showing specific recognition of mH2A1.1 isoforms by Ab αmH2A1.1 antibody. HEK-293T cells were transfected with plasmids coding for Flag-mH2A1.1 (Flag-1.1) or Flag-mH2A1.2 (Flag-1.2) fusion overexpressed-proteins. Western blot was then done with Ab αmH2A1.1, Ab αFlag and Ab αE215 (that preferentially recognizes mH2A1.2) antibodies on whole cell extracts. GAPDH is used as a loading control. (B) Immunofluorescence in HEK-293T cells showing specific recognition of mH2A1.1 isoform by Ab αmH2A1.1. DNA is labelled with Hoechst. Scale bar = 10 µm. (C) Western blot on ChIP extracts from HEK-293T cells overexpressing Flag-1.1 or Flag-1.2 showing that Ab αmH2A1.1 immunoprecipitates only mH2A1.1 isoform. Different extracts were loaded: Input fraction (Input), Non immunoprecipitated fraction (NoIP) and immunoprecipitated fraction (IP). Percentages represent fraction loaded on western blot compared to quantity used for ChIP. (D) Western blot showing that Ab αmH2A1.1 is also working in ChIP in MDA-MB231 cells on the endogenous protein. (E) As in (A), but for Ab αmH2A1 (#37264) antibody showing that this antibody recognizes both isoforms but it less affine for Flag-1.1 than Ab αmH2A1.1. (F) As in (B), but for Ab αmH2A1 (#37264) antibody showing that this antibody recognizes both isoforms but it less affine for Flag-1.1 than Ab αmH2A1.1. (G) As in (C) but for Ab αmH2A1 (#37264) antibody showing that this antibody recognizes both isoforms but it less affine for Flag-1.1 than Ab αmH2A1.1. (H) mH2A1.1 binding at indicated genomic regions. Localisation of primers used for ChIPqPCR are shown in red. Neg.pos refers to a sequence to which mH2A1.1 is not bound. (I) Occupancy of mH2A1 isoforms at the regions presented in (H) (Top: Ab ?mH2A1.1; Bottom: Ab ?mH2A1) analysed by ChIP-qPCR in control cells (WT) and cells partially deficient for mH2A1.1 using two different siRNA (mH2A1.1 KD #1 and mH2A1.1 KD #2). Error bars represent s.d from independent biological experiments (n>=2).

Journal of Cell Science • Supplementary information

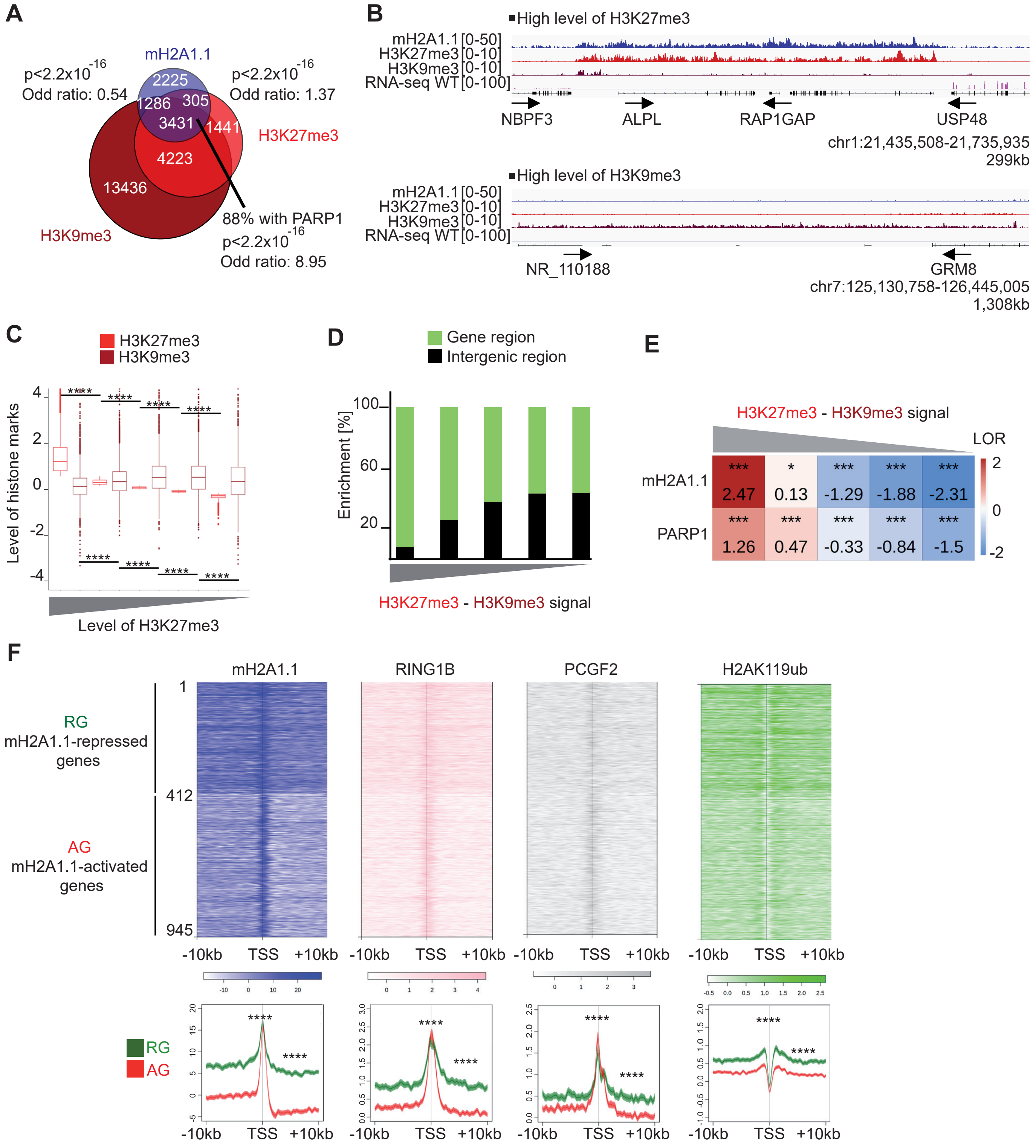

Journal of Cell Science • Supplementary information

Fig. S3. mH2A1.1 binds facultative heterochromatin domains and actively transcribed target genes.

(A) Overlap of heterochromatin histone marks (H3K27me3 and H3K9me3) with mH2A1.1 peaks. Enrichment of mH2A1.1 with PARP1 peaks was done on heterochromatin domains. Genome-wide enrichments of mH2A1.1 peaks with heterochromatin histone marks are measured with fisher exact tests p-values (p) and the Odd ratios are shown. (B) Genome browser view illustrating occupancy of mH2A1.1 with heterochromatin histone marks (H3K27me3 and H3K9me3). Top: region with high level of H3K27me3. Bottom: region with high level of H3K9me3. Unstranded RNA-seq signal is also shown. The black arrows show the direction of transcription. (C) Boxplots showing H3K27me3 and H3K9me3 enrichment levels on H3K27me3-H3K9me3 common peaks. Common peaks were divided into 5 equal size categories according to the level of H3K27me3, as indicated. Wilcoxon tests were used to compare conditions. ****: p-value < 2.2x10-16. (D) Histogram showing proportions of heterochromatin (H3K27me3-H3K9me3 common peaks) on genomic regions (green) or intergenomic regions (black). Heterochromatin peaks were divided into 5 equal size categories according to difference between H3K27me3 and H3K9me3 signal, as mentioned. (F) Fisher test heatmap showing enrichment of indicated ChIP-seq peaks (overlapping with common heterochromatin peaks) with heterochromatin peaks divided in 5 equal size categories as a function of differences between H3K27me3 and H3K9me3 signals. Stars indicate the significatively of the fisher exact tests; color map and values present in each scare highlight the log2 odd ratio (LOR) of the fisher exact test. (F) Top panel: Heatmap profiles showing relative enrichment of indicated proteins and histone modifications around the TSS (+/- 10 kb) of mH2A1.1-regulated genes (see Fig. 1A). On the top, mH2A1.1-repressed genes (1 to 412, n=412), on the bottom, mH2A1.1-activated genes (412 to 945, n=533). Color intensity reflects level of ChIP -seq enrichment. Heatmaps are oriented. Bottom panel: Metagene profiles of average (+/- standard error) of indicated ChIP-seq data around the TSS (+/- 10 kb) of mH2A1.1-regulated genes. Average profiles around the TSS of mH2A1.1-repressed genes are shown in green whereas average profiles around the TSS of mH2A1.1-activated genes are shown in red. Results of statistical difference analysis between these two groups are shown, either on the TSS (+/- 50 bp) or on the gene body (+50 bp – TES). Wilcoxon tests were used to compare conditions. ****: p-value < 2.2x10-16.

Journal of Cell Science • Supplementary information

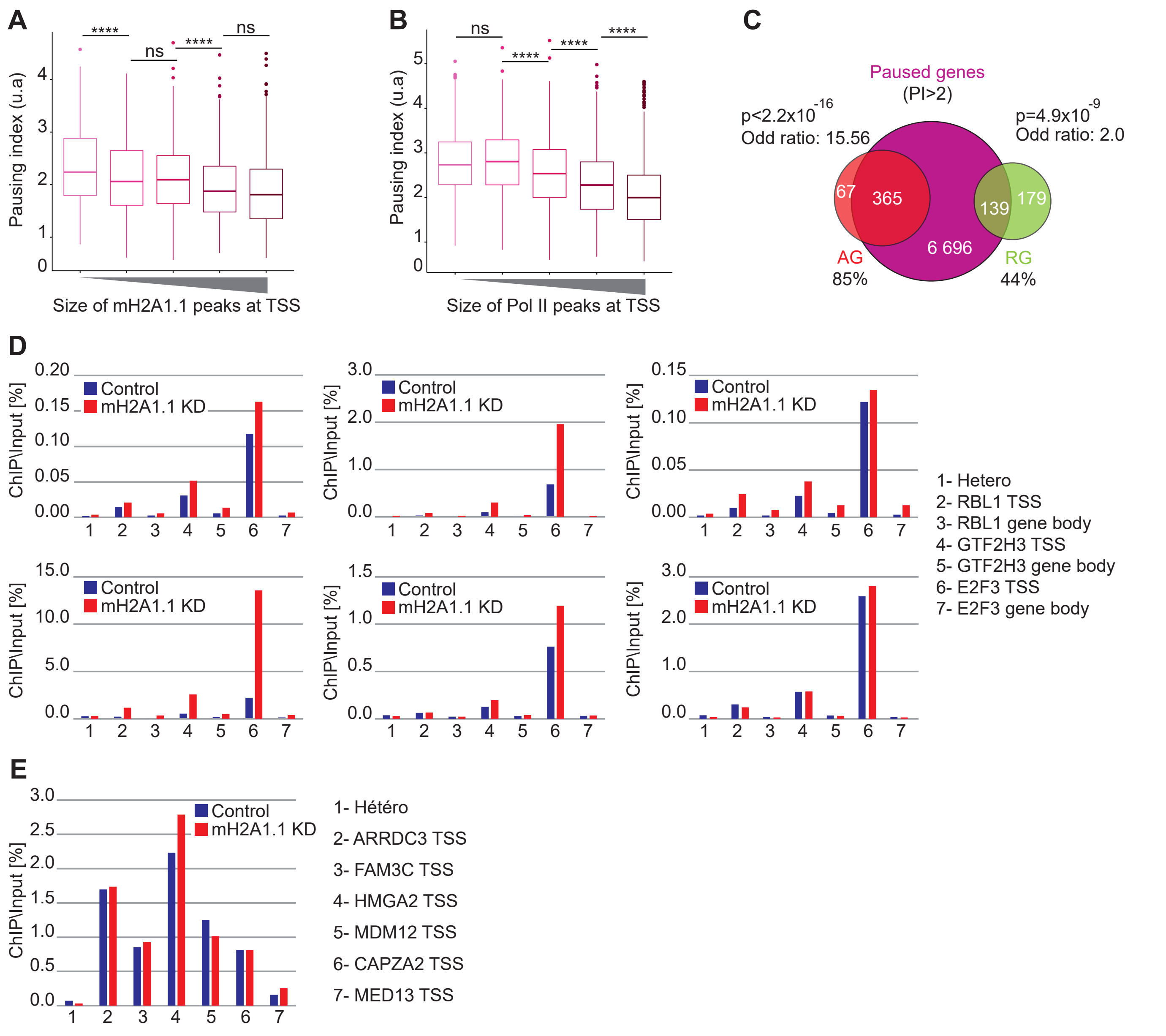

Fig. S4. mH2A1.1 favours Pol II pausing release. (A) Boxplot comparing the pausing index of 5 categories of mH2A1.1-bound genes divided according to the width of mH2A1.1 peaks. Wilcoxon tests were used to compare conditions. ****: p-value < 2.2x10-16, ns: not significant. (B) Same as in (A) but for Pol II-bound genes. (C) Overlap of mH2A1.1-regulated genes with paused genes. Enrichment of mH2A1.1-target genes with paused genes are measured using fisher exact tests. p-values (p) and the Odd ratios are shown. Of note, only mH2A1.1-target genes characterized by a PI were used to generate this Venn diagram. (D) Biological replicates of ChIPqPCR of Pol II in control and mH2A1.1 KD conditions. The first biological replicate is shown Fig 4E. (E) ChIPqPCR of Pol II in control and mH2A1.1 KD conditions on mH2A1.1-activated genes that lose interactions with adjacent genomic regions (see Fig. S6C). The first biological replicate in shown Fig. 6H.

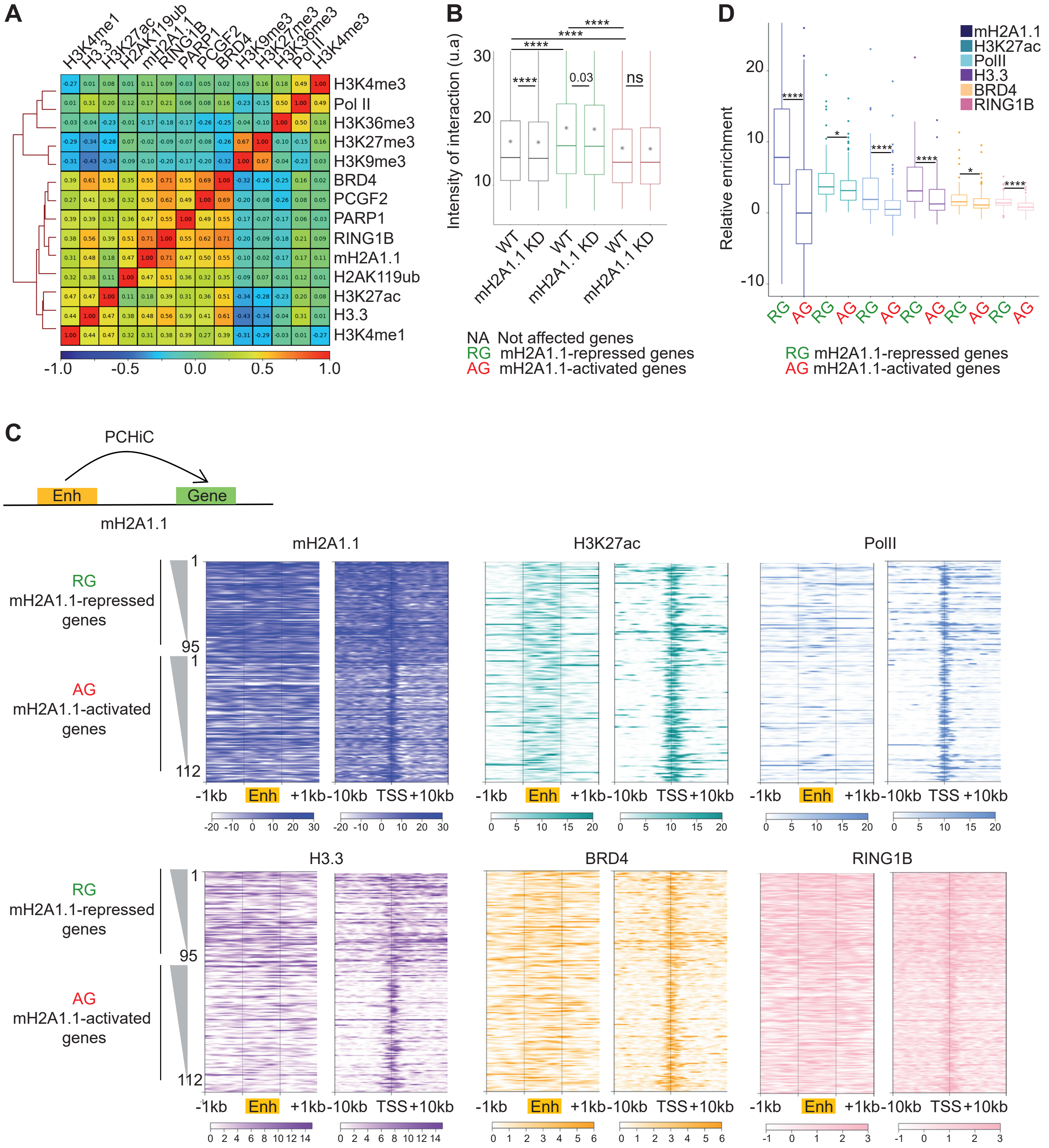

Fig. S5. The mH2A1.1 isoform binds enhancers of mH2A1.1-repressed genes. (A) "Putative" enhancers centered spearman correlation heatmap of ChIP-seq data. Correlations shown as in Fig 1D. Enhancers are based on H3K27ac signal outside promoter regions using the ROSE package (Blinka et al., 2017). (B) Boxplot showing the intensity of PCHiC interactions between genes, mH2A1.1-repressed genes (n=181) and mH2A1.1-activated genes (n=282) in control and mH2A1.1 KD conditions with their respective enhancers. Enhancers of mH2A1.1 -regulated genes were determined using PCHIC data and enhancer annotations (Materials and Methods). Paired wilcoxon tests were used to compare control and mH2A1.1 KD conditions whereas unpaired wilcoxon tests were used to compare gene categories. ns: not significant, ****: p-value < 2.2x10-16. (C) Heatmap profiles showing ChIP-seq data relative enrichment around the TSS (+/- 10 kb) of mH2A1.1-regulated genes (right) and their associated enhancers (+/- 1kb) (left). Enhancers of mH2A1.1-regulated genes were determined using PCHIC data and enhancer annotations (Materials and Methods). More than one enhancer can interact with mH2A1.1-regulated genes, but for sake of simplicity, only one enhancer per gene was randomly conserved to generate the presented heatmaps. Top: mH2A1.1-repressed genes (1 to 95). Bottom: mH2A1.1-activated genes (1 to 112). Genes are ranked according to their expression level differences between control and mH2A1.1 KD conditions. Some mH2A1.1-target genes are not present in the shown heatmaps because they did not have any PCHiC significant interactions with an enhancer or are not present in the PCHiC database. Colour intensity reflects level of ChIP-seq enrichment. TSS-centered heatmap profiles are oriented. (D) Boxplots comparing the relative enrichment of ChIP-seq data between the enhancers of mH2A1.1-repressed genes and the enhancers of mH2A1.1-activated genes. Wilcoxon tests were used to compare conditions. ns: not significant, ****: p-value < 2.2x10-16.

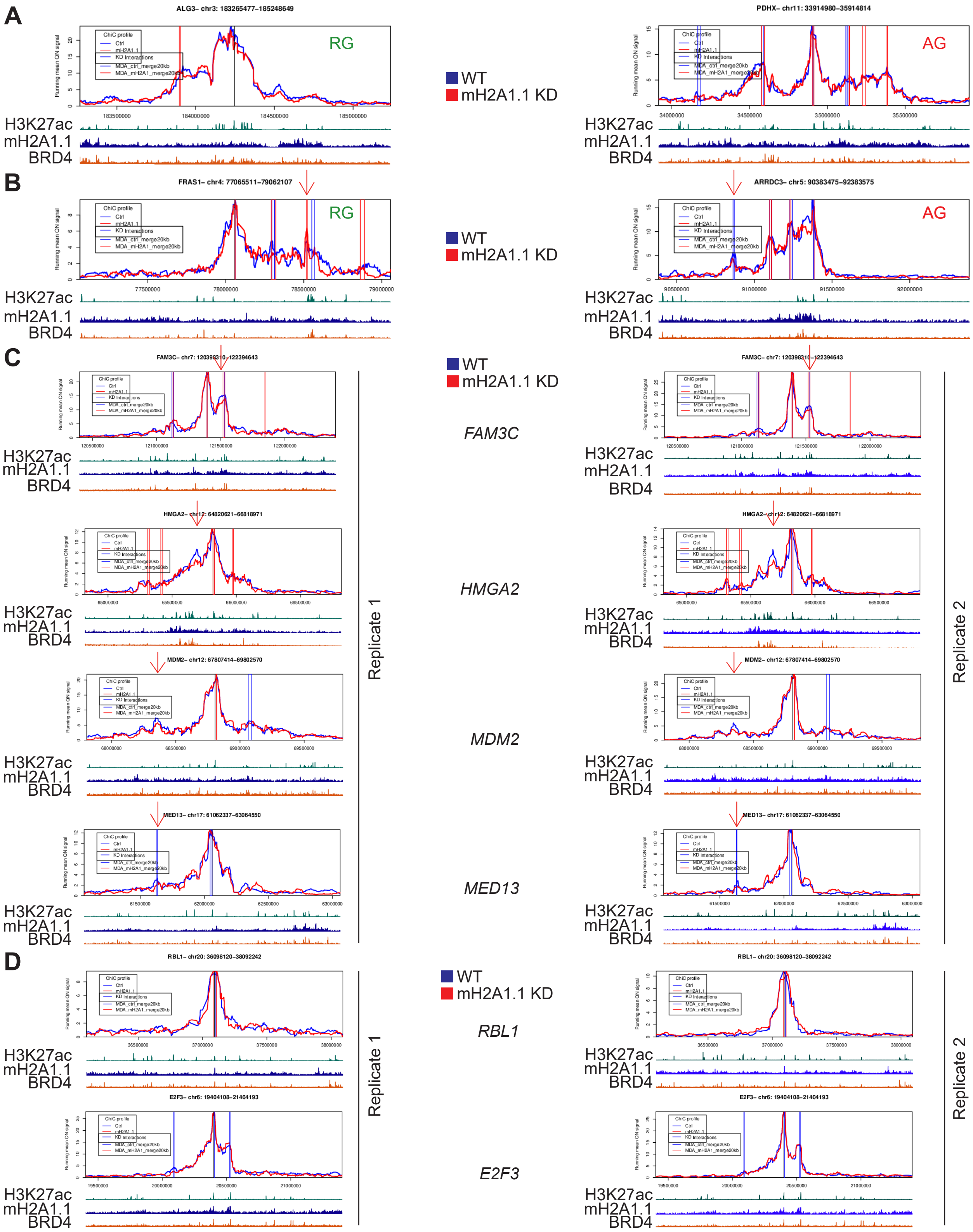

Fig. S6. Examples of local genomic interactions of mH2A1.1-target genes. (A) Snapshots of PCHiC data set (replicates n°2) on one mH2A1.1-repressed gene (left) and one mH2A1.1-activated gene (right) in control and mH2A1.1 KD conditions, as indicated. Same legend as in Fig 6C. (B) Same as in (A) but for one mH2A1.1 -repressed gene on the left and a mH2A1.1-activated gene on the right. Replicates n° 2 are shown. (C) Snapshots of PCHiC data set of 4 mH2A1.1-activated genes as indicated, in control and mH2A1.1 KD conditions. Replicates n°1 and 2 are shown, on the left and on the right, respectively. (D) as in (C) but for two mH2A1.1-activated genes used in Fig 4E. The gene GTF2H3 was not sequenced in our PCHiC data.

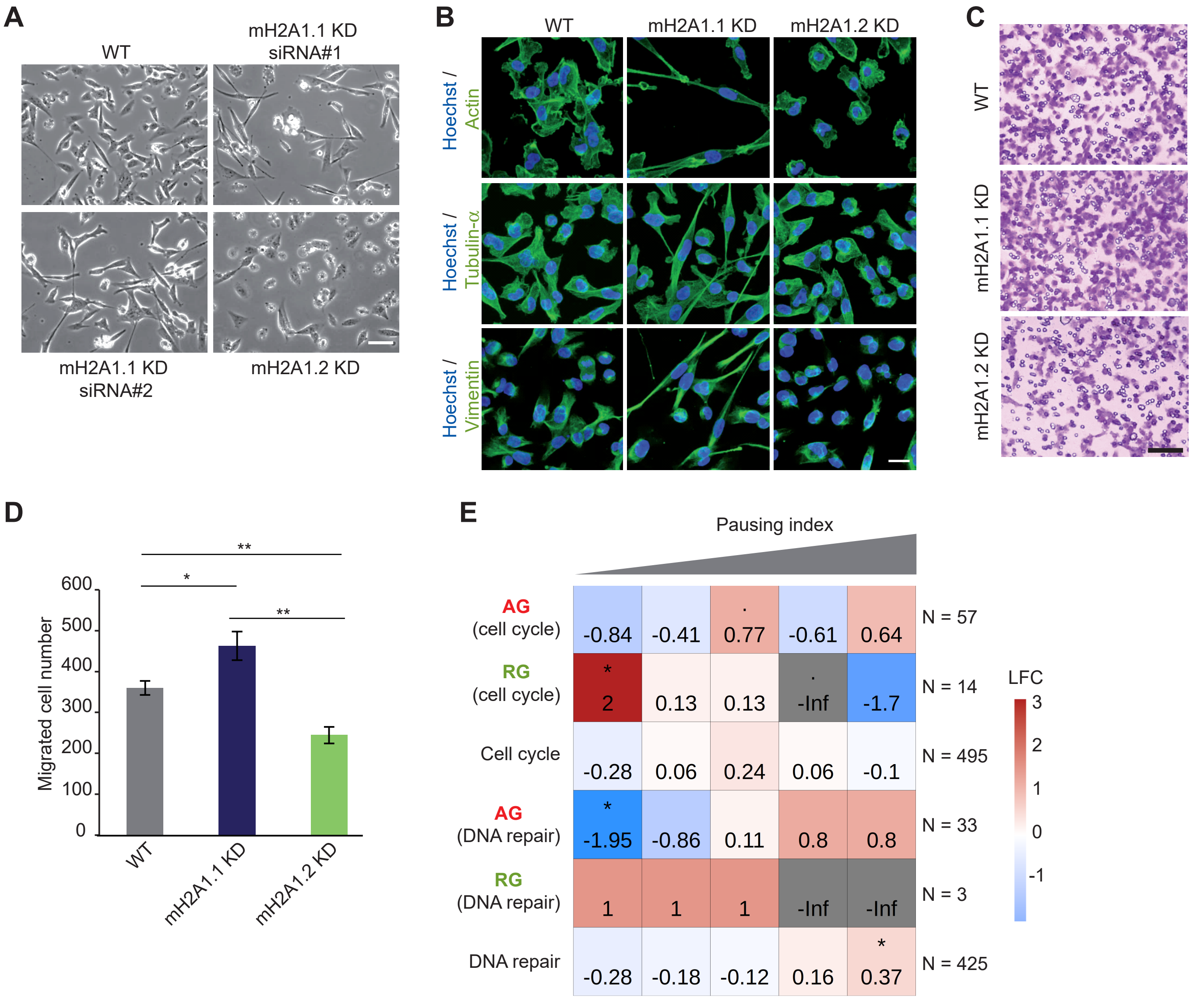

Fig. S7. mH2A1.1 and mH2A1.2 have opposite roles on cell migration in MDA-MB231 cells.
(A) Representative DIC microscopy images of WT, mH2A1.1 KD (two different siRNA) and mH2A1.2 KD MDA-MB231 cells. Scale bar = 100 µm. (B) Immunofluorescence of Actin (up), Tubulin-α (middle) and Vimentin (down) in WT, mH2A1.1 KD and mH2A1.2 KD MDA-MB231 cells. Nuclei are stained with Hoechst. Scale bar = 20 µm. (C) Boyden chamber assay representative images of WT, mH2A1.1 KD and mH2A1.2 KD MDA-MB231 cells. Only migrated cells are labelled in purple. Scale bar = 200 µm. (D) Quantification of Boyden chamber assay presented in (C). Error bar represents s.d from n=3 independent experiments. Wilcoxon tests were used to compare conditions. *: p-value (p) < 0.05, **: $p < 0.01$. (E) Fisher test heatmap showing enrichment of indicated genes (implicated either in cell cycle or in DNA repair). Genes are divided into 5 equal size categories as a function of their pausing index. Stars indicate the significance of the fisher exact tests; color map and values present in each square highlight the log2 odd ratio (LOR) of the fisher exact test. N indicates the number of genes used for the analysis.

Journal of Cell Science • Supplementary information

Table S1. mH2A1.1 activated genes

Click here to download Table S1

Table S2 . mH2A1.1 repressed genes

Click here to download Table S2

Table S3. mH2A1.1 activated paused genes

Click here to download Table S3

Journal of Cell Science • Supplementary information

Table S4. List of antibodies

Antibody	Company	catelog #	Dilution for western blot	Dilution for Immunofluorescence	Amount/ChIP	note	Reference
H3	Abcam	Ab1791	1:2000	-	-	-	
GAPDH	Millipore	MAB374	1:500	-	-	-	
mH2A1.2	Cell signaling	4827	1:1000	1:200	-	-	
mH2A1	Abcam	Ab37264 (Ab αmH2A1)	1:1000	1:300	10 µg	-	
mH2A1	Milipore	ABE215	1:1000			ChIPseq & ChIPqPCR	
mH2A1.1	Home-made	(Ab αmH2A1.1)	1:1000	1:200	10 µg	ChIPseq & ChIPqPCR	
Pol II	Santa Cruz Biotechnology	Pol II (F12) sc-55492			1 µg	ChIPqPCR	
Flag	Sigma	F7425	1:500 (5-10 µg)	1:200	-	-	
Vimentin	Sigma	V6389	-	1:200	-	-	
Tubulin-α	Sigma	T6199	-	1:200	-	-	
H3K4me1	Abcam	Ab8895	-	-		ChIPseq	(Bejjani et al., 2021)
H3K4me3	Abcam	Ab8580	-	-		ChIPseq	(Bejjani et al., 2021)
H3K27ac	Abcam	Ab4729	-	-		ChIPseq	(Bejjani et al., 2021)
RNA Pol II	Santa Cruz Biotechnology	sc-55492X	-	-		ChIPseq	(Bejjani et al., 2021)
H3K36me3	Active motif	61101	-	-		ChIPseq	(Bejjani et al., 2021)
H3K27me3	Millipore	07-449	-	-		ChIPseq	(Franco et al., 2018)
H3K9me3	Abcam	ab8898	-	-		ChIPseq	(Franco et al., 2018)
BRD4	Bethyl	A301-985A100				ChIPseq	(Chan et al., 2018)
RING1B	Active Motif	#39663				ChIPseq	(Chan et al., 2018)
PCGF2	Santa Cruz Biotechnology	sc-10744X				ChIPseq	(Chan et al., 2018)
H2AK119ub	Cell signaling	#8240				ChIPseq	(Chan et al., 2018)
Anti-mouse-Peroxidase	Sigma	A2304	1:10.000	-	-	-	
Anti-Rabbit-Peroxidase	Sigma	A0545	1:10.000	-	-	-	
Anti-Rabbit-Peroxidase	Jackson ImmunoResearch	211-032-171	1:10.000	-	-	-	
Alexa Fluor 488 Anti-mouse	Invitrogen	A11029	1:1000	-	-	-	
Alexa Fluor 647 Anti-Rabbit	Invitrogen	A21245	1:1000	-	-	-	
Anti-Actin (CytoPainter Phalloidin-iFluor 488)	Abcam	ab176753		1:1000			

Table S5. List of NGS data

Summary of ChIPseq, RNAseq data and PCHiC data	Antibody used		GEO accession number	Sequencing method	# total reads (Million)	# STAR uniquely mapped reads	% STAR uniquely mapped reads	Note
	Company	catelog #						
H3K4me1	Abcam	Ab#8895	GSM4407526, GSM4407527,					(Bejjani et al.,2021)
H3K27ac	Abcam	Ab#4729	GSM4407530, GSM4407531,					(Bejjani et al.,2021)
H3K4me3	Abcam	Ab#8580	GSM4407528, GSM4407529,					(Bejjani et al.,2021)
H3K36me3	Active motif	#61101						
RNA Polymerase II	Santa Cruz Biotechnology	#sc-55492X	GSM4407533, GSM4407534,					(Bejjani et al.,2021)
mH2A1.1	Home-made	Ab αmH2A1.1	GSM4151570	HiSeq3000	69.8	61.8	88	
mH2A1	Abcam	Ab#37264 (Ab αmH2A1)	GSM4151571	HiSeq3000	53.4	47.2	88	
PARP1	Active motif	39559	GSM1517306					(Nalabothula et al., 2015)
H3.3	Millipore	17-10245	GSM3398219					(Ben Zouari et al., 2019)
H3K9me3	Abcam	ab#8898	GSM2258862, GSM2258863					(Franco et al., 2018)
H3K27me3	Millipore	#07-449	GSM2258850, GSM2258850					(Franco et al., 2018)
BRD4	Bethyl	A301-985A100	GSM2862187					(Chan et al., 2018)
RING1B	Active Motif	#39663	GSM2862179					(Chan et al., 2018)
PCGF2	Santa Cruz Biotechnology	sc-10744X	GSM2862185					(Chan et al., 2018)
H2AK119ub	Cell signaling	#8240	GSM2862181					(Chan et al., 2018)
RNAseq_WT_Rep1	-	-	GSM4151573	HiSeq3000	67.3	19.9	30	Total RNAseq
RNAseq_WT_Rep2	-	-	GSM4151574	HiSeq3000	54	15.3	28	Total RNAseq
RNAseq_mH2A1.1KD_Rep1	-	-	GSM4151575	HiSeq3000	42.1	10.7	26	Total RNAseq
RNAseq_mH2A1.1KD_Rep2	-	-	GSM4151576	HiSeq3000	26.5	4.6	17	Total RNAseq
PCHIC_WT_Rep1			GSM5714290	HiSeq 4000				
PCHIC_WT_Rep2			GSM5714292	HiSeq 4000				
PCHIC_mH2A1.1KD_Rep1			GSM5714291	HiSeq 4000				
PCHIC_mH2A1.1KD_Rep2			GSM5714293	HiSeq 4000				

Table S6. Gene ontology of AG

Click here to download Table S6

Table S7. Gene ontology of RG

Click here to download Table S7

Journal of Cell Science • Supplementary information

Table S8. siRNA sequences and plasmids

Peptide, Plasmid and siRNA	targeting sequence (5'-3')	Notes
Peptide used to design Ab-αmH2A1.1	$_{197}$CQWQADIASIDSDAVVHPTGTDFYIGGEV$_{225}$	
siRNA mH2A1.1#1_F	GUUGUACAGGCUGACAUUG	
siRNA mH2A1.1#1_R	CAAUGUCAGCCUGUACAAC	
siRNA mH2A1.1#2_F	CGACAAACACUGACUUCUA	(Dardenne et al, 2011)
siRNA mH2A1.1#2_R	UAGAAGUCAGUGUUUGUCG	
siRNA mH2A1.2_F	GGCUUUGAGGUGGAGGCCAUAAUCA	(Dell'Orso et al, 2016)
siRNA mH2A1.2_R	UGAUUAUGGCCUCCACCUCAAAGCC	

Journal of Cell Science • Supplementary information

Table S9. qPCR primers

qPCR Primers	sequence (5'-3')	qPCR Primers	sequence (5'-3')
mH2A1.1_F	GGCTTCACAGTCCTCTCCAC	Peak4_R	CACGGTAAATGCCCCAGAAG
mH2A1.1_R	GGTGAACGACAGCATCACTG	Peak5_F	AAATAATTTCGGCCGGGTCG
mH2A1.2_F	GGCTTCACAGTCCTCTCCAC	Peak5_R	TCGAATTCCTGGGCTCAAGT
mH2A1.2_R	GGATTGATTATGGCCTCCAC	Neg.pos_F	TCAGTTAATCCTCCCACCCC
RPLP0_F	TGGCAGCATCTACAACCCTGAA	Neg.pos_R	TGACAAACACACAGAACAGACA
RPLP0_R	CACTGGCAACATTGCGGACA	Hetero_F	GCAGCTGTTTGTGTTTGGTG
MZT1_F	GACAGGATTTCAGCCACCAC	Hetero_R	AGGGAACAGATGAAGGGGTG
MZT1_R	TTAGCTGCCCAACAAACTGT	RBL1 TSS_F	TGGGCGCCAAACATAATCTG
CXCL8_F	ACTTTCAGAGACAGCAGAGCA	RBL1 TSS_R	CACCAATCCTTCCCCTCTGT
CXCL8_R	CTTCACACAGAGCTGCAGAA	RBL1 gene body_F	GACGCAGAAGAGGAAATTGGA
MAPK6_F	CTCTTCCTCGCCCTCTCTC	RBL1 gene body_R	GCTGTTGAAGGTTATACTCCACA
MAPK6_R	CAGTGTCGGCTCAGGTCTC	GTF2H3 TSS_F	AAATAATTTCGGCCGGGTCG
MT1E_F	TGTGCCAAGTGTGCCCAG	GTF2H3 TSS_R	TCGAATTCCTGGGCTCAAGT
MT1E_R	AATCCAGGTTGTGCAGGTTG	GTF2H3 gene body_F	TCAGTTAATCCTCCCACCCC
HKDC1_F	AGCATGTCTGTACCATCGTCT	GTF2H3 gene body_R	TGACAAACACACAGAACAGACA
HKDC1_R	TGAGGGTGTATCTTGTAGAGGG	E2F3 TSS_F	GGGAGGAGAGAAGGAGGAGA
COLA1A_F	TGGTTTCGACTTCAGCTTCC	E2F3 TSS_R	GTGCCCTTTTGTCCATGGAG
COLA1A_R	ATGTTCTCGATCTGCTGGCT	E2F3 gene body_F	ACGTCTCTTGGTCTGCTCAC
JUND_F	GACGAGCTCACAGTTCCTCT	E2F3 gene body_R	TCTTCTTAATGAGGTGGATGCC
JUND_R	TCAGGTTCGCGTAGACAGG	ARRDC3 TSS_F	GCCGGCGTTTCTAGATTCAG
TARS_F	TTGATCATCGGCCAAGGTCC	ARRDC3 TSS_R	TGCTGGGAAAGGTGAAGAGT
TARS_R	GGTGAGTCCTGTGAGTGCTC	FAM3C TSS_F	TGACCCACCCATCCTAGAGA
RPL10A_F	CTTAAGTCCACTCCCCGCC	FAM3C TSS_R	CTGTTCTTCTTGGGTGGTGC
RPL10A_R	GCCCTCGATGTCCATGTG	HMGA2 TSS_F	ACTTGAATCTTGGGGCAGGA
Peak1_F	GGTGGCTGTAACTCTCTCGT	HMGA2 TSS_R	AGTCGGAAAGCAAAGGAGGA
Peak1_R	CCAGGCCCCAGATGATAGAG	MDM2 TSS_F	ACCAGCATCTTCGTTCTCCA
Peak2_F	AGGCTGGACTTATGGGTTCC	MDM2 TSS_R	CCGTGTCGCTGTTACCAAAA
Peak2_R	ACTTTACCCCTCTGTGCCT	CAPZA2 TSS_F	ATGGAGAGGGTCGTGATGTG
Peak3_F	AGCTTCCCAGACTCCCTTTC	CAPZA2 TSS_R	ATATGGGCTCAGTCTGCGAT
Peak3_R	CGTCGCCTAACAATTCCGAG	MED13_F	GCCACTAACATAGCGCCATC
Peak4_F	AGGTATTTCGTCTGTCCCCG	MED13_R	CGGCCTCGCGAAATAAATGA
