# Supplementary material for: The histone variant macroH2A1.1 regulates RNA polymerase II-paused genes within defined chromatin interaction landscapes
Source: J Cell Sci. 2022 Apr 11;135(7):jcs259456. doi: 10.1242/jcs.259456 (PMC9016624; doi:10.1242/jcs.259456)
Supplement: Reviewer comments [file joces-135-259456_review_history.pdf]

The histone variant macroH2A1.1 regulates RNA Polymerase II paused genes within defined chromatin interaction landscapes.

Ludmila Recoules, Alexandre Heurteau, Flavien Raynal, Nezih Karasu, Fatima Moutahir, Fabienne Bejjani, Isabell Jariel-Encontre, Olivier Cuvier, Tom Sexton, Anne-Claire Lavigne and Kerstin Bystricky
DOI: 10.1242/jcs.259456

Editor: Caroline Hill

Review timeline

Submission to Review Commons:	25 June 2021
Submission to Journal of Cell Science:	6 October 2021
Editorial decision:	11 October 2021
First revision received:	7 December 2021
Editorial decision:	24 January 2022
Second revision received:	18 February 2022
Accepted:	28 February 2022

Reviewer 1

Evidence, reproducibility and clarity

Summary:

Here the authors used a triple negative breast cancer (TNBC) cell line (MDA-MB231) expressing high levels of macroH2A1.1 to obtain a detailed map of genomic regions bound by this histone and to highlight the functional significance of the corresponding particular genomic localization.

Technically macroH2A1.1 as well as Pol II and a series of histone marks (H3K27ac, H3K4me1, H3K4me3, H3K36me3) were mapped following the authors' own ChIPs. They also included published ChIP-seq data (BRD4, RINGIB, PCFG2 and H2AK119ub) in their analyses to enlarge their view of macroH2A1.1 chromatin landscape in the studied cell line.

Their main conclusions are summarized below.
macroH2A1.1 is predominantly associated with "facultative-like heterochromatin".
macroH2A1.1 binds enhancer and promoter regions.
macroH2A1.1 enhances gene expression by releasing Pol II pause at a subset of genes, including a group of anti-migratory genes.
macroH2A1.1 binds enhancers and super-enhancers in association with BRD4 and RING1B.
macroH2A1.1 is not significantly involved in genomic interactions between promoters and other genomic regions at the mH2A1.1 regulated genes, and the role of mH2A1.1 in Pol II release does not involve a modulation of chromatin looping.

Major comments:

1 - This study comprehensively describes the genome-wide map of mH2A1.1 in a single human breast cancer cell line and considers some functional consequences of these

© 2022. Published by The Company of Biologists under the terms of the Creative Commons Attribution License (https://creativecommons.org/licenses/by/4.0/).

observations following mH2A1.1 knock-down. These approaches allowed the authors to draw the interesting conclusions summarized above.
The experimental approaches and data analyses appear sound and they convincingly back the conclusions.
However, it is not clear if the described properties and activities of mH2A1.1 are specific to this cell line, to triple negative breast cancer, or whether they could stand generally in all systems expressing significant amounts of mH2A1.1.
Although it could be too much to carry out this research in several cell systems in parallel, it would have been very useful, if the authors could at least use the available mH2A1.1 mapping data to check some of their conclusions on different cancer and non-cancer cell lines and in various physiological contexts.

2 - An intriguing observation is the accumulation of mH2A1.1 at the nucleosome free regions (i e Fig. 2E).
This is an unexpected observation. Could the authors better characterize this phenomenon? Do the authors know any histone variant that accumulate at the active gene NFRs?
Is the association of mH2A with NFR observed in any other system?

This observation should be highlighted and specifically discussed.

3 - The authors should consider that the knock-down of mH2A1.1 may generate local histone under-dosage. Indeed, if the lack of mH2A1.1 is not compensated by other H2As, a modified chromatin may appear at regions originally containing mH2A1.1. Since histones combines structural and regulatory roles, it is important that the authors highlight in their discussions that the phenotypes associated with KD/KO approaches could also reflect defective chromatin organizations.

Significance

Here the authors present data that could support important conclusions and that would significantly increase our understanding of mH2A1.1 functions.

However, unfortunately, the current experimental setting does not allow to know whether the reported observations correspond to general properties of mH2A1.1. or are associated with the specific "epigenetic" landscape of TNBC or of this particular cell line.

Any indication that would allow the authors to draw some more general conclusions would greatly enhance the significance of this work.

Reviewer 3

Evidence, reproducibility and clarity

Summary:

This manuscript by Bystricky and colleagues describes a thorough investigation of the chromatin associations and functional implications of the macroH2A1.1 histone variant. MacroH2A1.1 is one of two alternative splice variants of the macro-histone variant macroH2A1, the other being macroH2A1.2. This manuscript is to this reviewers knowledge the first to describe genome-wide mapping of macroH2A1.1 chromatin occupancy. To do so, the authors have generated and extensively validated a macroH2A1.1 splice variant-specific polyclonal antibody. Knowing the genomic regions that are affected by either macroH2A1 variant is anticipated to advance our understanding of their often distinct

© 2022. Published by The Company of Biologists under the terms of the Creative Commons Attribution License (https://creativecommons.org/licenses/by/4.0/).

biological roles. In this manuscript, the authors use their ChIP-Seq findings to provide additional insight into macroH21.1-mediated regulation of gene expression. MacroH2A1.1 loss has been shown to both activate and repress gene expression, and the authors now propose a link between macroH2A1.1 intensity and the status of RNA Polymerase II (P0111) pausing at transcription start sites as a predictor of expression outcome. Genetic evidence for macroH2A1.1dependent PolII pausing is compelling. A model involving looping between TSSs and macroH2A1.1-enriched enhancers is proposed. However, given that macroH2A1.1 does not alter loop formation, this adds more confusion than clarification. It is further unclear what accounts for the seemingly distinct modes of macroH2A1.1 accumulation at repressed and activated genes. The authors end with a demonstration of functional relevance for cell migration, which extends previous work by invoking a contribution of macroH2A1.1dependent PolII pausing (e.g. PMID: 22542848).

Major comments:

1) The link between macroH2A1.1 at promoters, increased transcription and Pol II pausing is intriguing. However, little mechanistic detail is provided to explain how macroH2A1.1 is enriched at active promoters, and how it modulates PolII pausing. This is a major missed opportunity, particularly given the link between Pol II pausing and PARP1, which also interacts with macroH2A1.1 (e.g. PMID: 31940791). It would be interesting to investigate a possible relationship between PARP activity and recruitment macroH2A1.1 at TSSs. It is further unclear if macroH2A1.1 accumulation at these regions involves nucleosome incorporation, particularly since its accumulation at genes with paused PolII overlaps with nucleosome free regions.

2) While the epigenetic characterization of the TNBC cell line is expansive, the absence of inclusion of a macroH2A1.2 ChIP-Seq experiment, or comparison to existing macroH2A1.2 ChIP-Seq data (if available in the same cell line), is a significant limitation, as this would help corroborate the proposed, variant-specific effects described here, particularly at promoter TSSs. Is accumulation at TSSs specific to macroH2A1.1?

3) It is counterintuitive that actively transcribed macroH2A1.1-enriched genes, which show mid to high gene expression levels (Fig 2C), have high levels of PolII pausing and little to no detectable gene body PolII (Fig 4A). This should be addressed in the discussion. Why would lower pausing in the presence of macroH2A1.1 cause lower expression?

4) Details antibody generation need to be added to the methods section (antigen, host, etc)

Minor comments:

1) How do the authors exclude that macroH2A1.1 enrichment at enhancers (or promoters) is not an artifact of pairing between promoters and enhancers, and hence reflecting enrichment at only one of the two paired sites? Additionally, the fraction of paired promoter / enhancers with and without macroH2A1.1 should be assessed. Are promoter /enhancer pairs enriched in macroH2A1.1?

2) What fraction of genes with high PolII pause index show macroH2A1.1 enrichment? Is there a correlation to PARP, if so, what is the overlap? It may be helpful to speculate why this is particularly relevant at genes involved in cell migration.

3) Based on previous work, the correlation of macroH2A1 / macroH2A1.1 with H3K27me3 is expected to be stronger. This should be discussed.

© 2022. Published by The Company of Biologists under the terms of the Creative Commons Attribution License (https://creativecommons.org/licenses/by/4.0/).

4) Page 5: "macroH2A1.1 correlated with the level of [...] PolII, H3.3 and Brd4" Coincides would be a better choice, as correlation (PCC) between these markers has not been assessed at TSSs.

5) Is macroH2A1.1 subject to "pruning as recently described by PMID 30291361?

Significance

Understanding the unique and overlapping chromatin enrichment profiles for the two macroH2A1 splice variants will significantly improve our understanding of how these variants exert their often distinct functions. Lack of a comparison with macroH2A1.2 ChIP-Seq in the same cell line makes this, however, difficult.

The different modes of macroH2A1.1 binding at activated and repressed promoters is intriguing and provides insight into distinct gene-regulatory modes for macroH2A1.1. These findings would further benefit from additional mechanistic insight.

Novelty of this work mainly lies within the finding that macroH2A1.1 activates genes by modulating PolII pausing. Functional consequences of macroH2A1.1. loss for cell migration largely validate previous work, as stated in the manuscript. The specificity for these pathways is nevertheless intriguing.

Author response to reviewers' comments

1. General Statements [optional]

Dear Editor,

we submit our manuscript 'The histone variant macroH2A1.1 regulates RNA Polymerase II paused genes within defined chromatin interaction landscapes.' by Recoules et al. for consideration for publication. The manuscript was reviewed through Review Commons.

Both reviewers praised the quality of our study and the new insights gained for the mechanism through which the histone variant macroH2A1.1 regulates transcription, in particular through moderating RNA polymerase pausing. We highlight that mH2A1.1 mediated regulation depends on specific genomic landscapes but does not involve ad hoc changes in chromatin fiber looping. These mechanisms are particularly relevant for the human triple negative breast cancer line studied in which mH2A1.1 is overrepresented compared to other cancer cell lines, yet may open new avenues for comprehending cell specific intrinsic transcriptional programs.

Following the reviews, we added further discussion and analysis of genome wide data in the manuscript or in comments included in this letter.

We thank the reviewers for their valuable contribution improving our study.

Please find the point-by-point replies to the reviewer's comments below.

With kind regards,

2. Description of the planned revisions

3. Description of the revisions that have already been incorporated in the transferred manuscript

© 2022. Published by The Company of Biologists under the terms of the Creative Commons Attribution License (https://creativecommons.org/licenses/by/4.0/).

Reviewer 1 _ Point 1:

Line 352

Currently, only one study has analyzed transcriptional activities of mH2A1.1 related to its genomic localization, and that is in murine muscle cell line C2C12 (Hurtado-Bages et al., 2020). As in our study, although mH2A1.1 is mainly implicated in repression of transcription, a significant proportion of genes requires mH2A1.1 for their level of transcription. Among the activated genes, mH2A1.1 is enriched on genes encoding proteins related to adhesion, migration and the organization of extracellular matrix. But at the difference of our study, this recruitment occurs upstream of the TSS of these genes, the level of mH2A1.1 occupancy on the bodies of genes reducing with increasing transcriptional activity. However, Lavigne et al, identified a restricted recruitment of the mH2A1.2 isoform to TSS of mH2A1-regulated genes in two cancer cell types, HeLa and Namalwa cells (Lavigne et al., 2015) even if a comparison of genomic sites bound by mH2A1.2 nucleosomes revealed only a small overlap between HeLa and Namalwa cells. Similar, restricted recruitments at TSS were also observed for mH2A1 and mH2A2 in human et mouse embryonic stem cells (Pliatska et al., 2018; Yildirim et al., 2014). Therefore, we believe that the mH2A variants are differentially recruited to regulatory sites depending on carcinogenic and differentiation state of the cells. In breast cancers, recruitment and thus the roles of mH2A1 variants must be subtype specific. The newly identified recruitment of mH2A1.1 would thus be TNBC specific, as not identified so far in luminal breast cancer cell lines (Gamble et al., 2010). It could then explain why we found a correlation between mH2A1.1 expression levels and survival rates only in TNBC patients (Lavigne et al., 2014).

Reviewer 2 _ Major comments_Point 4:

The method section refers to the host - rabbit - and gives the peptide sequence used. Figure Sup1 shows controls and complete characterization.

Line 801

Rabbit anti-mH2A1.1 antibody was generated according to immunization protocol from Agro-Bio - La Fierté Saint-Aubin – France.

Reviewer 2 _ Minor comments_Point 4:

Line 169

At promoters, mH2A1.1 distribution inversely coincided with heterochromatin marks (**Fig 2A & S5A, S5B Fig**).

Thank you. The sentence was changed but the PCC are presented in Figure 2A.

4. Description of analyses that authors prefer not to carry out

Reviewer 1 _ Point 2:

We and others (Lavigne et al., 2015; Yildirim et al., 2014) observed mH2As in a manner around the TSS to place the maximum binding to the nucleosome free region (NFR). Different hypotheses could explain those results. The first one could be that mH2A isoforms are not incorporated into the chromatin and bind the NFR as a transcription factor. Indeed, upon DSBs, Xu et al, 2012 showed that mH2A1 binds DSB chromatin without being incorporated into a nucleosome (Xu et al., 2012). However, Lavigne et al, detected TSS-bound mH2A1.2 by native ChIP, excluding in part this hypothesis (Lavigne et al., 2015). A second hypothesis could be that mH2As are incorporated into the two adjacent nucleosomes of the TSSs but their macro domain could be joined at the NFR, explaining the higher signal at the NFR obtained compared to the two adjacent nucleosomes. Yet another hypothesis could be that mH2A isoforms are incorporated into “labile nucleosomes” frequently found at the not really ‘NFR’ and only observed using low salt ChIP experiments (done for all the mentioned publications and ours here), as similarly to H3.3 and H2AZ (Jin et al., 2009). Future analysis will be necessary to discriminate between these analyses.

© 2022. Published by The Company of Biologists under the terms of the Creative Commons Attribution License (https://creativecommons.org/licenses/by/4.0/).

Reviewer 1 _ Point 3:

Indeed, we can not exclude that depletion of the histone variant mH2A1.1 or mH2A1.2 or any other histone creates labile nucleosomes, possibly tetrasomes (Bohm et al., 2011) which may change accessibility and affect transcriptional activity indirectly. However, siRNA treatment was performed over 72h including at least 2 or 3 replication cycles during which H2A core histone is expressed and incorporated, most likely, at sites which originally contained mH2A1. Moreover, since looping is not affected, it seems unlikely that chromatin structure massively changes at the studied sites.

Reviewer 2 _ Major comments_Point 1:

We thank the reviewer for proposing to test the relationship between PARP1 and mH2A1.1 to explain its recruitment and its effect on transcriptional activation potentially due to the well known interaction between the two proteins (Chen et al., 2014) and the relationship between PARP1 and Pol II pausing (Gibson et al., 2016).

For several reasons, however, we chose not to investigate this aspect further in the present study. The first reason is the genomic localization of PARP1 in MDA-MB 231 does not show a significant enrichment of this protein at mH2A1.1-target genes (**Figure 1**). The second reason is that Hurtado et al showed that the transcriptional activator effect of mH2A1.1 is independent of PARP1 in C2C12 muscle mouse cells (Hurtado-Bages et al., 2020).

In our study, we identify many potential partners of mH2A1.1 which could participate in its positive effect on Pol II pausing release such as BRD4, RING1B, PCGF2, and H3.3. Although these analyses make one want to better characterize the underlying molecular mechanisms of these factors, we believe that testing the interconnection between all these partners is beyond the scope of this article.

© 2022. Published by The Company of Biologists under the terms of the Creative Commons Attribution License (https://creativecommons.org/licenses/by/4.0/).

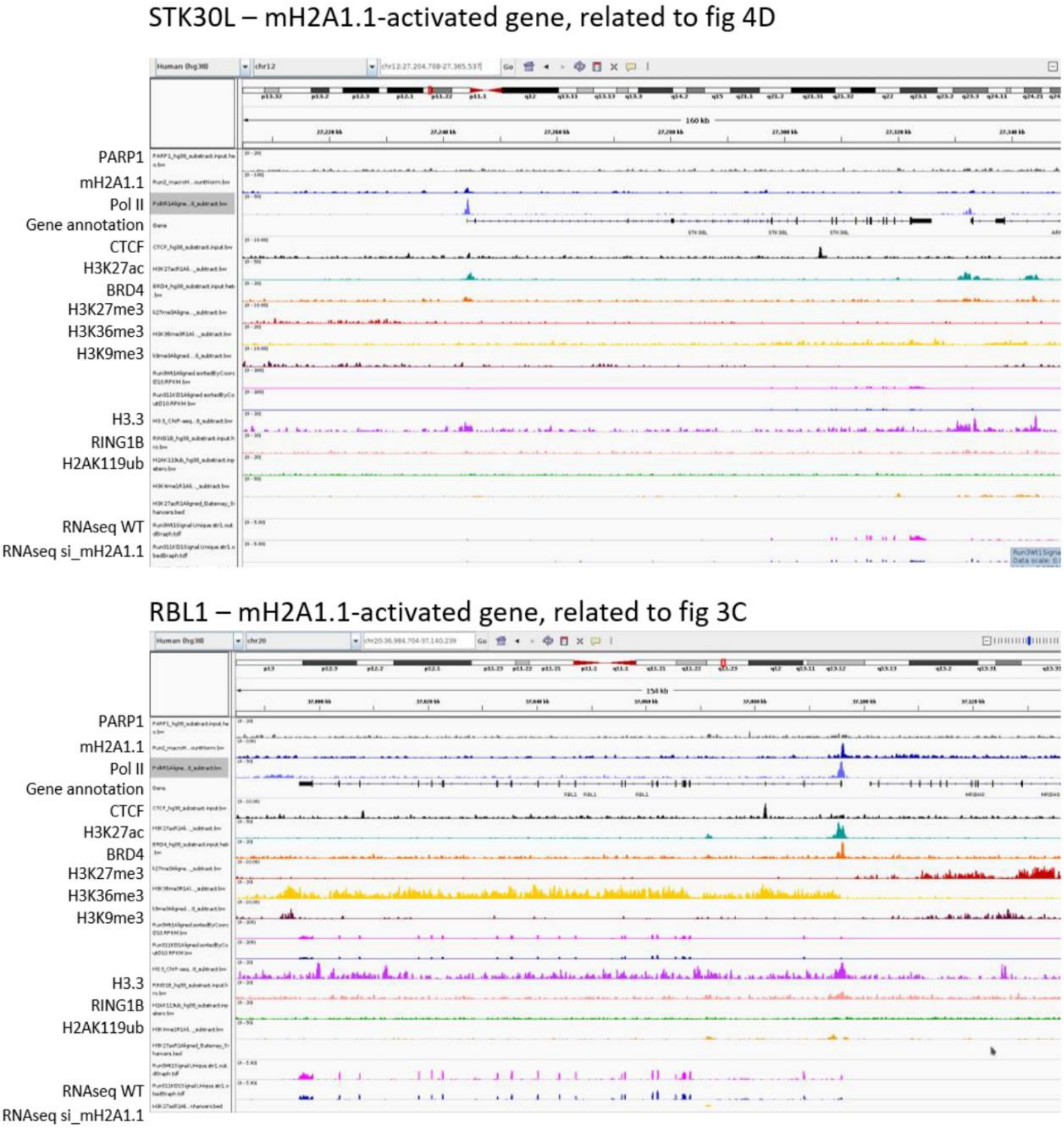

Figure 1: IGVs showing localization of PARP1 at two mH2A1.1-activated genes. IGVs show also others ChIP-seq data.

Reviewer 2 _ Major comment_Point 2:

Unfortunately, we were unable to test the competition hypothesis, since, in our hands, all tested antibodies failed to specifically detect mH2A1.2 in chromatin immunoprecipitation (Bystricky and Buschbeck labs). Only one research group did ChIP-seq of mH2A1.2 but they used their own mH2A1.2-specific antibody (Lavigne et al., 2015; Pliatska et al., 2018).

Reviewer 2 _ Major comment_Point 3:

Highly transcribed genes have been shown to be frequently pol II paused genes (Core and Adelman, 2019).

Fig. 2C shows that mH2A1.1 accumulates at actively transcribed genes. In this analysis, we did not discriminate paused and not paused genes. The role of mH2A1.1 at all of these genes can be inhibitory or stimulatory (Fig 3). Yet, only genes activated by mH2A1.1 (AG) have a significantly elevated pausing index (Fig 4D). Expression of these genes is lowered in the absence of mH2A1.1 likely because Pol II is no longer properly released (greater pausing). As a matter of fact, we demonstrate that the presence of mH2A1.1 seems to stimulate pause release to boost expression.

© 2022. Published by The Company of Biologists under the terms of the Creative Commons Attribution License (https://creativecommons.org/licenses/by/4.0/).

Hence, in its absence pausing is increased and leads to lower expression. Increased presence of Pol II over the gene body (Fig 4A) was used to define genes not in pause.

Reviewer 2 _ Minor comment_Point 1:

Indeed, so-called indirect peaks could be due to crosslinking artifacts. However, indirect peaks are generally of very low intensity, which is not at all what we observe. To be certain, we compared the signal strengths from mH2A1.1 to enhancers and promoters bound by mH2A1.1 (**Figure 2**). As a control, we did the same analysis but with Pol II ChIP-seq data. We found that Pol II signal is much higher at promoters compared to enhancers as expected. However, we found quasi similar intensity signals for mH2A1.1 at enhancers and promoters. This strongly suggests that peaks observed at either enhancers or promoters correspond to direct association of mH2A1.1 to those sites and not "crosslinking artifacts".

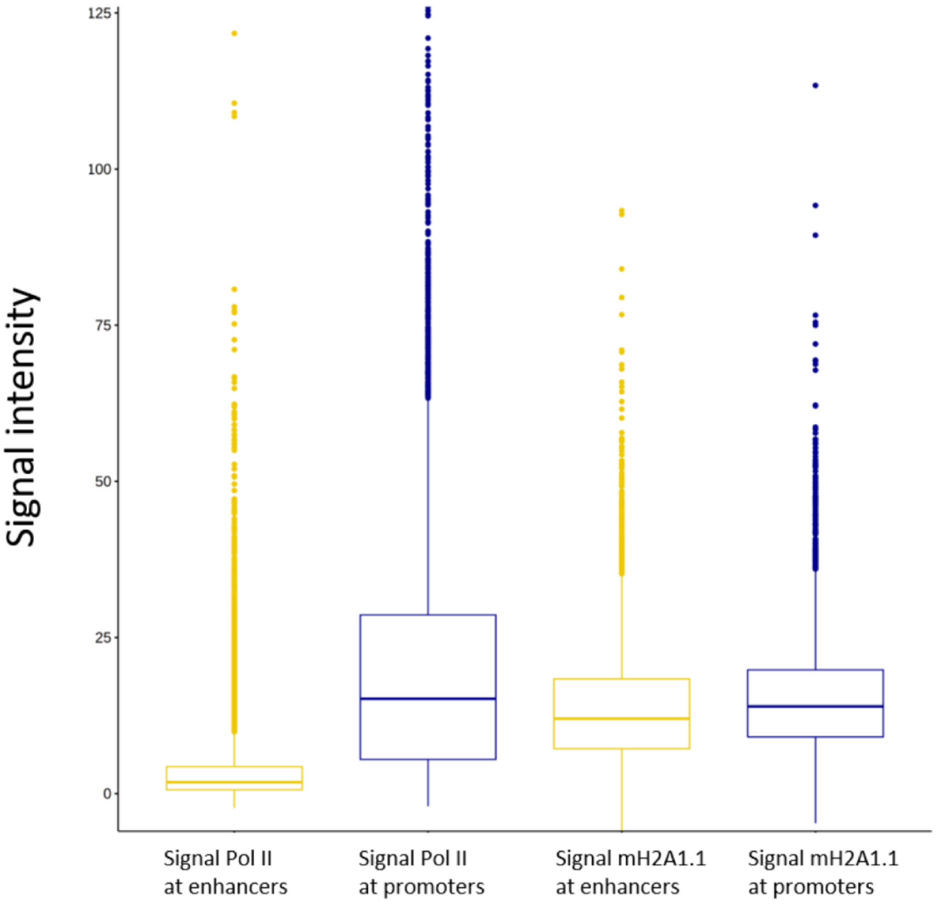

Figure 2: Boxplot showing the signal intensity of ChIP-seq data on enhancers and promoters. Only enhancers and promoters bind either by Pol II or mH2A1.1 were taken for the analysis.

We found that around 50 % of enhancer/promoter contact sites are bound by mH2A1.1 either on the enhancer or the promoter. More details are given in **Table n°1** and **Figure 3**. Of note, those fractions remain unchanged in the mH2A1.1 KD condition.

© 2022. Published by The Company of Biologists under the terms of the Creative Commons Attribution License (https://creativecommons.org/licenses/by/4.0/).

			Fraction of interaction (%)		Comments
**Number of interaction**	**WT**	**si_mH2A1.1**	**WT**	**si_mH2A1.1**	
All interaction	15610	16515	100	100	
Interaction enh/pro	3869	3980	24,785394	24,0993037	fraction of all interaction
Interaction enh/pro no mH2A1.1	1942	2002	50,1938485	50,3015075	fraction of interaction enh/pro
Interaction enh/pro mH2A1.1 only at pro	333	395	8,60687516	9,92462312	fraction of interaction enh/pro
Interaction enh/pro mH2A1.1 only at enh	762	734	19,6950116	18,4422111	fraction of interaction enh/pro
Interaction enh/pro mH2A1.1 at enh & pro	832	849	21,5042647	21,3316583	fraction of interaction enh/pro
Interaction enh/pro mH2A1.1 at enh or pro	1927	1978	49,8061515	49,6984925	fraction of interaction enh/pro

Table 1: Table showing the number of interactions and the corresponding relative fractions, as indicated in control (WT) and mH2A1.1 KD (si_mH2A1.1) conditions. enh = enhancers, pro = promoters

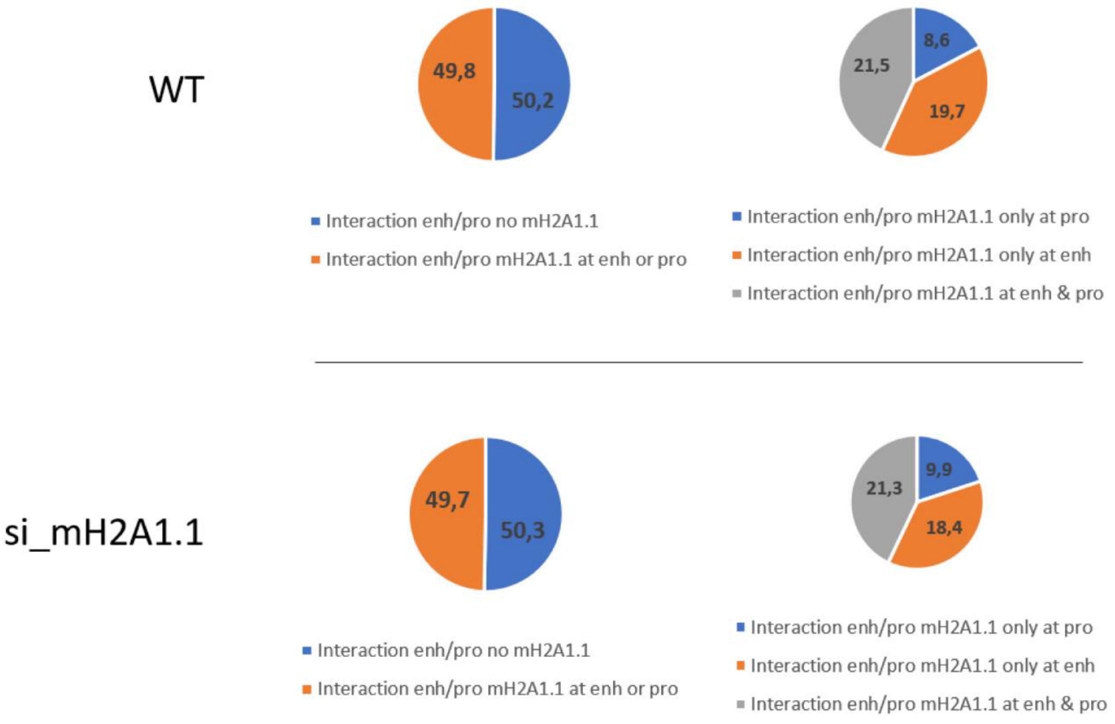

Figure 3: Pie charts showing percentages of interaction between enhancers and promoters bound or not by mH2A1.1. Percentages are also summarized in the Table 1.

© 2022. Published by The Company of Biologists under the terms of the Creative Commons Attribution License (https://creativecommons.org/licenses/by/4.0/).

Reviewer 2 _ Minor comment_Point 2:

This fraction is around 10 % of highly paused (HP) genes. The number of highly paused genes is n=2000 among 10546 genes for which we could determine a pausing index. It is logically a small fraction since mH2A1.1 does not define paused genes. Hence, the TSS of 226 genes highly in pause is bound by mH2A1.1 (**Figure** *4)*.

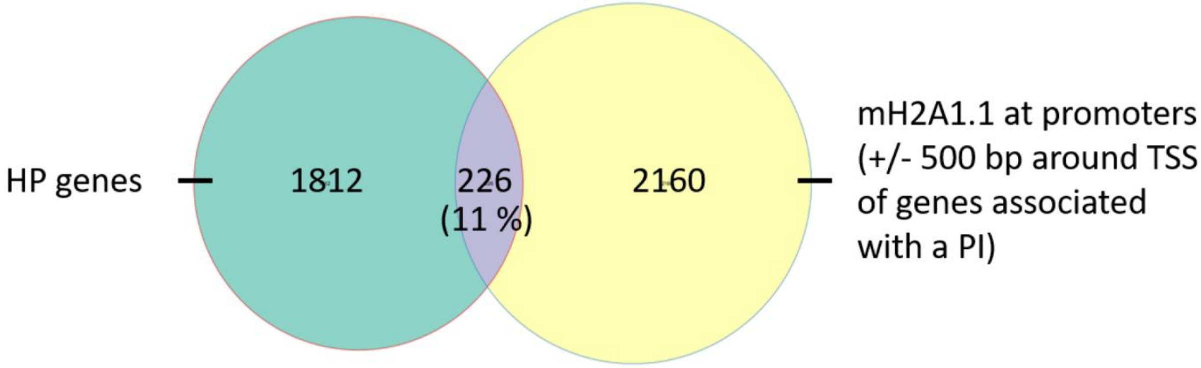

Figure 4: Venn diagram showing the overlap between highly paused (HP) genes and mH2A1.1-related peaks at promoters of PI-associated genes.

We did not say that mH2A1.1 is enriched at paused genes but we demonstrate that the size of the mH2A1.1 peaks at the TSS of mH2A1.1 regulated genes is inversely proportional to the pausing level of the gene (**Figure 5**).

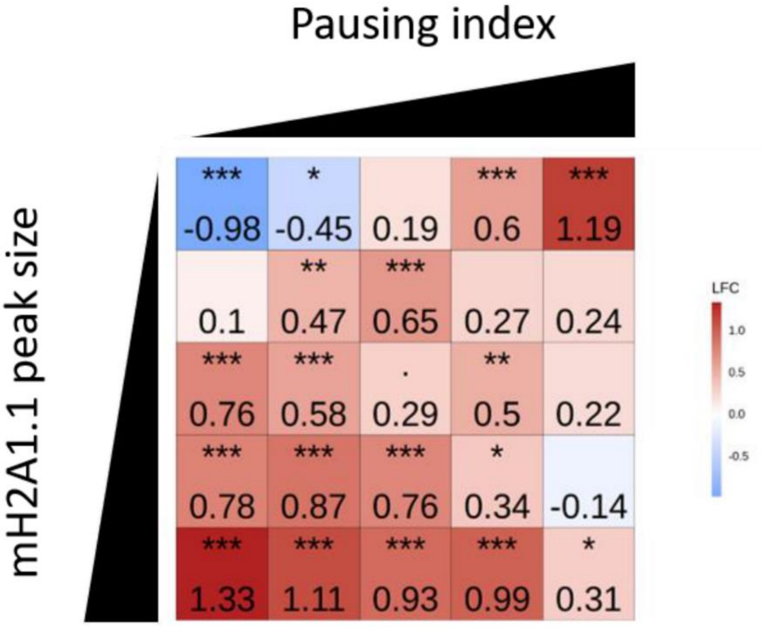

Figure 5: Correlation heatmap showing enrichment of mH2A1.1 peaks at TSS (+/- 500bp) based on mH2A1.1 peak size and polymerase II pausing index (PI). mH2A1.1 narrow peaks at TSS are enriched at genes with a high PI whereas large mH2A1.1 peaks at TSS are enriched at genes with low PI.

Of the 226 highly paused genes bound by mH2A1.1, 180 were bound by PARP1 (**Figure 6**). However, we found PARP1 (and also BRD4) associated with >80% of highly paused genes in general (**Figure 7**), so this is not a feature specific to mH2A1.1-bound genes.

© 2022. Published by The Company of Biologists under the terms of the Creative Commons Attribution License (https://creativecommons.org/licenses/by/4.0/).

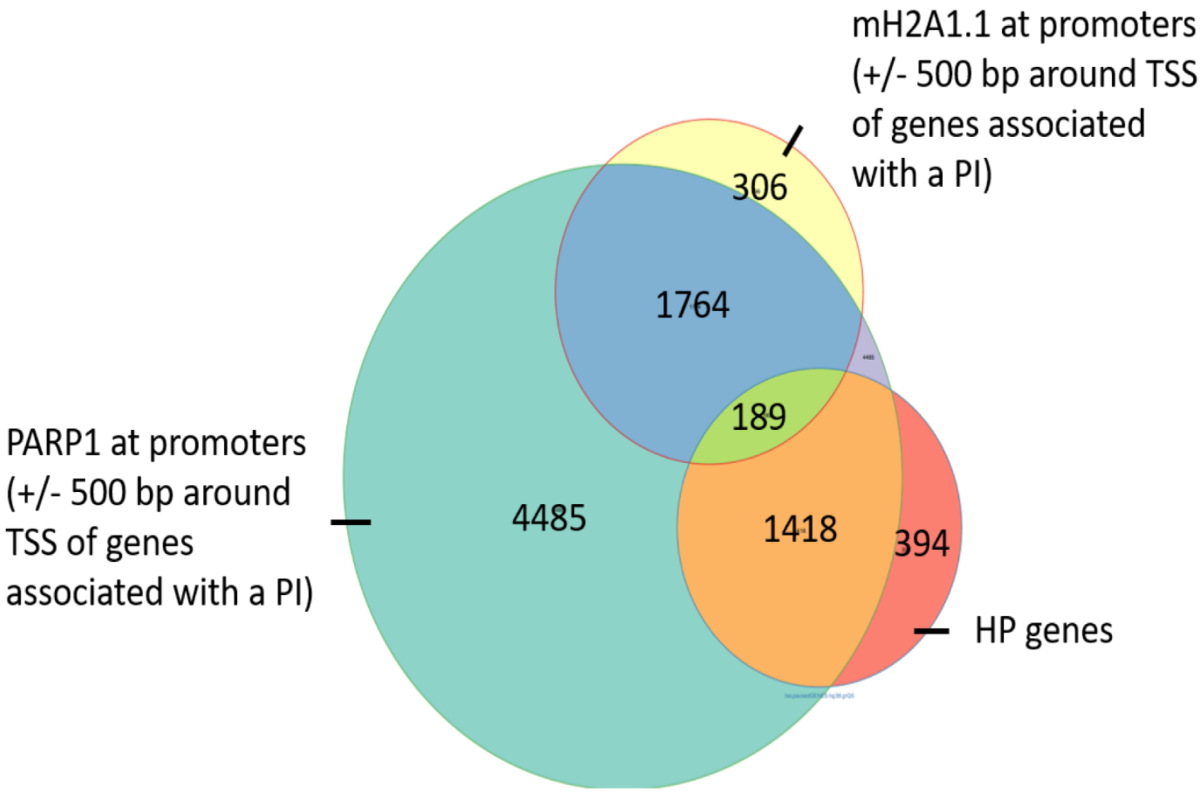

Figure 6: Venn diagram showing the overlap between highly paused (HP) genes, mH2A1.1-related peaks at promoters of PI-associated genes and PARP1.

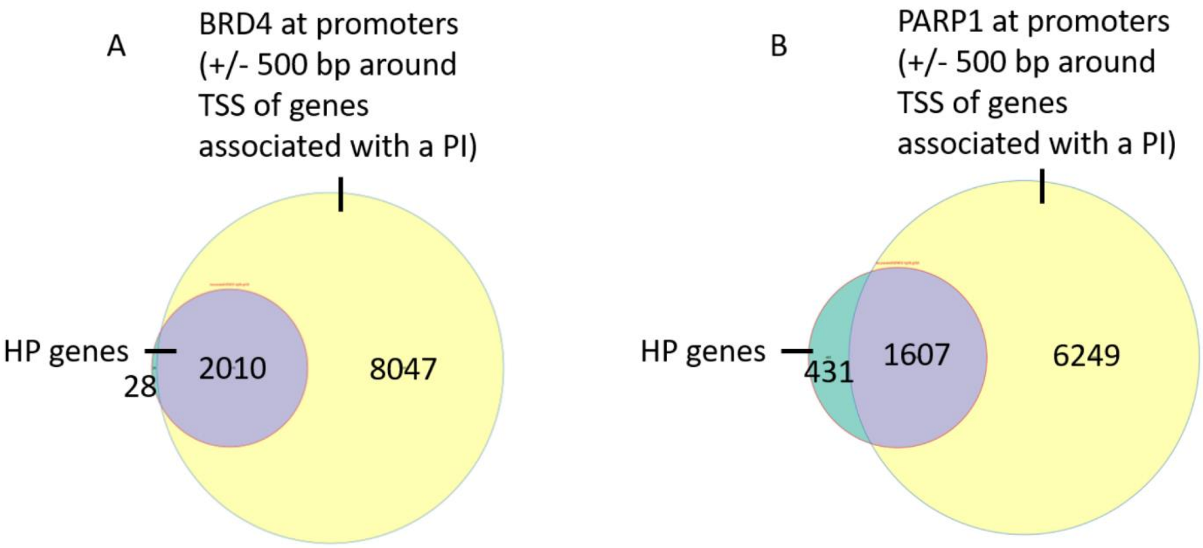

Figure 7: Venn diagram showing the overlap between highly paused (HP) genes with BRD4 (A) or PARP1 (B).

We speculate that the role of mH2A1.1 at cells involved in migration is particularly relevant because their expression needs to be quickly adapted to changing environments in response to stimuli. Rapid gene regulation can be efficiently achieved through pause release mechanisms.

Reviewer 2 _ Minor comment_Point 3:

Fig. 1D and Fig. sup 4A of the article illustrate that most of mH2A1.1 (>50% of sites bound) associates and correlates with H3K27me3. We feel that this is described and discussed.

© 2022. Published by The Company of Biologists under the terms of the Creative Commons Attribution License (https://creativecommons.org/licenses/by/4.0/).

Reviewer 2 _ Minor comment_Point 5:

We did not test it. Over large domains, this could be possible, in particular since pruning was shown for mH2A1 without distinguishing isoforms (Sun et al, 2018). At regions where mH2A1.1 binding was detected as narrow peaks, pruning appears unlikely and deposition may require a different mechanism such as chaperone-mediated deposition independently of the genome duplication.

References:

Bohm, V., Hieb, A.R., Andrews, A.J., Gansen, A., Rocker, A., Toth, K., Luger, K., and Langowski, J. (2011). Nucleosome accessibility governed by the dimer/tetramer interface. Nucleic acids research *39*, 3093-3102.

Chen, H., Ruiz, P.D., Novikov, L., Casill, A.D., Park, J.W., and Gamble, M.J. (2014). MacroH2A1.1 and PARP-1 cooperate to regulate transcription by promoting CBP-mediated H2B acetylation. Nature structural & molecular biology *21*, 981-989.

Core, L., and Adelman, K. (2019). Promoter-proximal pausing of RNA polymerase II: a nexus of gene regulation. Genes & development *33*, 960-982.

Gamble, M.J., Frizzell, K.M., Yang, C., Krishnakumar, R., and Kraus, W.L. (2010). The histone variant macroH2A1 marks repressed autosomal chromatin, but protects a subset of its target genes from silencing. Genes & development *24*, 21-32.

Gibson, B.A., Zhang, Y., Jiang, H., Hussey, K.M., Shrimp, J.H., Lin, H., Schwede, F., Yu, Y., and Kraus, W.L. (2016). Chemical genetic discovery of PARP targets reveals a role for PARP-1 in transcription elongation. Science *353*, 45-50.

Hurtado-Bages, S., Posavec Marjanovic, M., Valero, V., Malinverni, R., Corujo, D., Bouvet, P., Lavigne, A.C., Bystricky, K., and Buschbeck, M. (2020). The Histone Variant MacroH2A1 Regulates Key Genes for Myogenic Cell Fusion in a Splice-Isoform Dependent Manner. Cells *9*.

Jin, C., Zang, C., Wei, G., Cui, K., Peng, W., Zhao, K., and Felsenfeld, G. (2009). H3.3/H2A.Z double variant-containing nucleosomes mark 'nucleosome-free regions' of active promoters and other regulatory regions. Nature genetics *41*, 941-945.

Lavigne, A.C., Castells, M., Mermet, J., Kocanova, S., Dalvai, M., and Bystricky, K. (2014). Increased macroH2A1.1 expression correlates with poor survival of triple-negative breast cancer patients. PloS one *9*, e98930.

Lavigne, M.D., Vatsellas, G., Polyzos, A., Mantouvalou, E., Sianidis, G., Maraziotis, I., Agelopoulos, M., and Thanos, D. (2015). Composite macroH2A/NRF-1 Nucleosomes Suppress Noise and Generate Robustness in Gene Expression. Cell reports *11*, 1090-1101.

Pliatska, M., Kapasa, M., Kokkalis, A., Polyzos, A., and Thanos, D. (2018). The Histone Variant MacroH2A Blocks Cellular Reprogramming by Inhibiting Mesenchymal-to-Epithelial Transition. Molecular and cellular biology *38*.

Xu, C., Xu, Y., Gursoy-Yuzugullu, O., and Price, B.D. (2012). The histone variant macroH2A1.1 is recruited to DSBs through a mechanism involving PARP1. FEBS letters *586*, 3920-3925.

Yildirim, O., Hung, J.H., Cedeno, R.J., Weng, Z., Lengner, C.J., and Rando, O.J. (2014). A system for genome-wide histone variant dynamics in ES cells reveals dynamic MacroH2A2 replacement at promoters. PLoS genetics *10*, e1004515.

© 2022. Published by The Company of Biologists under the terms of the Creative Commons Attribution License (https://creativecommons.org/licenses/by/4.0/).

Original submission

First decision letter

MS ID#: JOCES/2021/259456

MS TITLE: The histone variant macroH2A1.1 regulates RNA Polymerase II paused genes within defined chromatin interaction landscapes.

AUTHORS: Ludmila Recoules, Alexandre Heurteau, Raynal Flavien, Nezih Karasu, Fatima Moutahir, Fabienne Bejjani, Isabell Jariel-Encontre, Olivier Cuvier, Tom Sexton, Anne-Claire Lavigne, and Kerstin Bystricky
ARTICLE TYPE: Research Article

We have now reached a decision on the above manuscript.

Your paper was reviewed by two reviewers for Review Commons. You have submitted a Revision Plan and a revised manuscript and appear to have answered some of the reviewer's concerns and make proposals of how others will be addressed. You also explain why you cannot do some of the other suggested experiments proposed by the Reviewers.

I would like you now to revise your manuscript thoroughly again, addressing all the reviewers' comments.Please provide a point-by-point response detailing how you have dealt with the points raised by the reviewers in the 'Response to Reviewers' box. Please attend to all of the reviewers' comments. If you do not agree with any of their criticisms or suggestions please explain clearly why this is so. Please address all the points for each reviewer in the order that the points arise.

In the revised manuscript, please highlight all the changes that have been made since the original version that was seen by the reviewers.
Please avoid using 'Tracked changes' in Word files as these are lost in PDF conversion.

First revision

Author response to reviewers' comments

Reviewer #1 (Evidence, reproducibility and clarity (Required)):

Summary:
Here the authors used a triple negative breast cancer (TNBC) cell line (MDA-MB231) expressing high levels of macroH2A1.1 to obtain a detailed map of genomic regions bound by this histone and to highlight the functional significance of the corresponding particular genomic localization.
Technically macroH2A1.1 as well as Pol II and a series of histone marks (H3K27ac, H3K4me1, H3K4me3, H3K36me3) were mapped following the authors' own ChIPs. They also included published ChIP-seq data (BRD4, RING1B, PCFG2 and H2AK119ub) in their analyses to enlarge their view of macroH2A1.1 chromatin landscape in the studied cell line.

Their main conclusions are summarized below.
macroH2A1.1 is predominantly associated with "facultative-like heterochromatin". macroH2A1.1 binds enhancer and promoter regions.
macroH2A1.1 enhances gene expression by releasing Pol II pause at a subset of genes, including a group of anti-migratory genes.
macroH2A1.1 binds enhancers and super-enhancers in association with BRD4 and RING1B.
macroH2A1.1 is not significantly involved in genomic interactions between promoters and other genomic regions at the mH2A1.1 regulated genes, and the role of mH2A1.1 in Pol II release does not involve a modulation of chromatin looping.

© 2022. Published by The Company of Biologists under the terms of the Creative Commons Attribution License (https://creativecommons.org/licenses/by/4.0/).

Major comments:

1 - This study comprehensively describes the genome-wide map of mH2A1.1 in a single human breast cancer cell line and considers some functional consequences of these observations following mH2A1.1 knock-down. These approaches allowed the authors to draw the interesting conclusions summarized above.
The experimental approaches and data analyses appear sound and they convincingly back the conclusions.
However, it is not clear if the described properties and activities of mH2A1.1 are specific to this cell line, to triple negative breast cancer, or whether they could stand generally in all systems expressing significant amounts of mH2A1.1.
Although it could be too much to carry out this research in several cell systems in parallel, it would have been very useful, if the authors could at least use the available mH2A1.1 mapping data to check some of their conclusions on different cancer and non-cancer cell lines and in various physiological contexts.

- *We have included a new paragraph in the manuscript to discuss this aspect of our work - Line 369*
  "Currently, only one study has analyzed transcriptional activities of mH2A1.1 related to its genomic localization, and that is in murine muscle cell line C2C12 (Hurtado-Bages et al., 2020). As in our study, although mH2A1.1 is mainly implicated in repression of transcription, a significant proportion of genes requires mH2A1.1 for their level of transcription. Among the activated genes, mH2A1.1 is enriched on genes encoding proteins related to adhesion, migration and the organization of extracellular matrix. But at the difference of our study, this recruitment occurs upstream of the TSS of these genes, the level of mH2A1.1 occupancy on the bodies of genes reducing with increasing transcriptional activity. However, Lavigne et al, identified a restricted recruitment of the mH2A1.2 isoform to TSS of mH2A1-regulated genes in two cancer cell types, HeLa and Namalwa cells (Lavigne et al., 2015) even if a comparison of genomic sites bound by mH2A1.2 nucleosomes revealed only a small overlap between HeLa and Namalwa cells. Similar, restricted recruitments at TSS were also observed for mH2A1 and mH2A2 in human et mouse embryonic stem cells (Pliatska et al., 2018; Yildirim et al., 2014). Therefore, we believe that the mH2A variants are differentially recruited to regulatory sites depending on carcinogenic and differentiation state of the cells. In breast cancers, recruitment and thus the roles of mH2A1 variants must be subtype specific. The newly identified recruitment of mH2A1.1 would thus be TNBC specific, as not identified so far in luminal breast cancer cell lines (Gamble et al., 2010). It could then explain why we found a correlation between mH2A1.1 expression levels and survival rates only in TNBC patients (Lavigne et al., 2014)."

2- An intriguing observation is the accumulation of mH2A1.1 at the nucleosome free regions (i e Fig. 2E). This is an unexpected observation. Could the authors better characterize this phenomenon?
Do the authors know any histone variant that accumulate at the active gene NFRs? Is the association of mH2A with NFR observed in any other system? This observation should be highlighted and specifically discussed.

- *We and others (Lavigne et al., 2015; Yildirim et al., 2014) observed mH2As in a manner around the TSS to place the maximum binding to the nucleosome free region (NFR). Different hypotheses could explain those results. The first one could be that mH2A isoforms are not incorporated into the chromatin and bind the NFR as a transcription factor. Indeed, upon DSBs, Xu et al, 2012 showed that mH2A1 binds DSB chromatin without being incorporated into a nucleosome (Xu et al., 2012). However, Lavigne et al, detected TSS-bound mH2A1.2 by native ChIP, excluding in part this hypothesis (Lavigne et al., 2015). A second hypothesis could be that mH2As are incorporated into the two adjacent nucleosomes of the TSSs but their macro domain could be joined at the NFR, explaining the higher signal at the NFR obtained compared to the two adjacent nucleosomes. Yet another hypothesis could be that mH2A isoforms are incorporated into "labile nucleosomes" frequently found at the not really 'NFR' and only observed using low salt ChIP experiments (done for all the mentioned publications and ours here), as similarly to H3.3 and H2AZ (Jin et al., 2009). Future*

© 2022. Published by The Company of Biologists under the terms of the Creative Commons Attribution License (https://creativecommons.org/licenses/by/4.0/).

analysis will be necessary to discriminate between these hypothesis.

3 - The authors should consider that the knock-down of mH2A1.1 may generate local histone under-dosage. Indeed, if the lack of mH2A1.1 is not compensated by other H2As, a modified chromatin may appear at regions originally containing mH2A1.1. Since histones combines structural and regulatory roles, it is important that the authors highlight in their discussions that the phenotypes associated with KD/KO approaches could also reflect defective chromatin organizations.

- *Indeed, we can not exclude that depletion of the histone variant mH2A1.1 or mH2A1.2 or any other histone creates labile nucleosomes, possibly tetrasomes (Bohm et al., 2011) which may change accessibility and affect transcriptional activity indirectly. However, siRNA treatment was performed over 72h including at least 2 or 3 replication cycles during which H2A core histone is expressed and incorporated, most likely, at sites which originally contained mH2A1. Moreover, since looping is not affected, it seems unlikely that chromatin structure massively changes at the studied sites.*

Reviewer #3 (Evidence, reproducibility and clarity (Required)):

Summary:

This manuscript by Bystricky and colleagues describes a thorough investigation of the chromatin associations and functional implications of the macroH2A1.1 histone variant. MacroH2A1.1 is one of two alternative splice variants of the macro-histone variant macroH2A1, the other being macroH2A1.2. This manuscript is to this reviewers knowledge the first to describe genome-wide mapping of macroH2A1.1 chromatin occupancy. To do so, the authors have generated and extensively validated a macroH2A1.1 splice variant-specific polyclonal antibody. Knowing the genomic regions that are affected by either macroH2A1 variant is anticipated to advance our understanding of their often distinct biological roles. In this manuscript, the authors use their ChIP-Seq findings to provide additional insight into macroH21.1-mediated regulation of gene expression. MacroH2A1.1 loss has been shown to both activate and repress gene expression, and the authors now propose a link between macroH2A1.1 intensity and the status of RNA Polymerase II (PolII) pausing at transcription start sites as a predictor of expression outcome. Genetic evidence for macroH2A1.1- dependent PolII pausing is compelling. A model involving looping between TSSs and macroH2A1.1-enriched enhancers is proposed. However, given that macroH2A1.1 does not alter loop formation, this adds more confusion than clarification. It is further unclear what accounts for the seemingly distinct modes of macroH2A1.1 accumulation at repressed and activated genes. The authors end with a demonstration of functional relevance for cell migration, which extends previous work by invoking a contribution of macroH2A1.1-dependent PolII pausing (e.g. PMID: 22542848).

Major comments:

1- The link between macroH2A1.1 at promoters, increased transcription and Pol II pausing is intriguing. However, little mechanistic detail is provided to explain how macroH2A1.1 is enriched at active promoters, and how it modulates PolII pausing. This is a major missed opportunity, particularly given the link between Pol II pausing and PARP1, which also interacts with macroH2A1.1 (e.g. PMID: 31940791). It would be interesting to investigate a possible relationship between PARP activity and recruitment macroH2A1.1 at TSSs. It is further unclear if macroH2A1.1 accumulation at these regions involves nucleosome incorporation, particularly since its accumulation at genes with paused PolII overlaps with nucleosome free regions.

- *We thank the reviewer for proposing to test the relationship between PARP1 and mH2A1.1 to explain its recruitment and its effect on transcriptional activation potentially due to the well known interaction between the two proteins (Chen et al., 2014) and the relationship between PARP1 and Pol II pausing (Gibson et al., 2016).*

For several reasons, however, we chose not to investigate this aspect further in the present study. The first reason is the genomic localization of PARP1 in MDA-MB 231 does not show a

© 2022. Published by The Company of Biologists under the terms of the Creative Commons Attribution License (https://creativecommons.org/licenses/by/4.0/).

*significant enrichment of this protein at mH2A1.1-target genes (**Figure 1**). The second reason is that Hurtado et al showed that the transcriptional activator effect of mH2A1.1 is independent of PARP1 in C2C12 muscle mouse cells (Hurtado- Bages et al., 2020).*

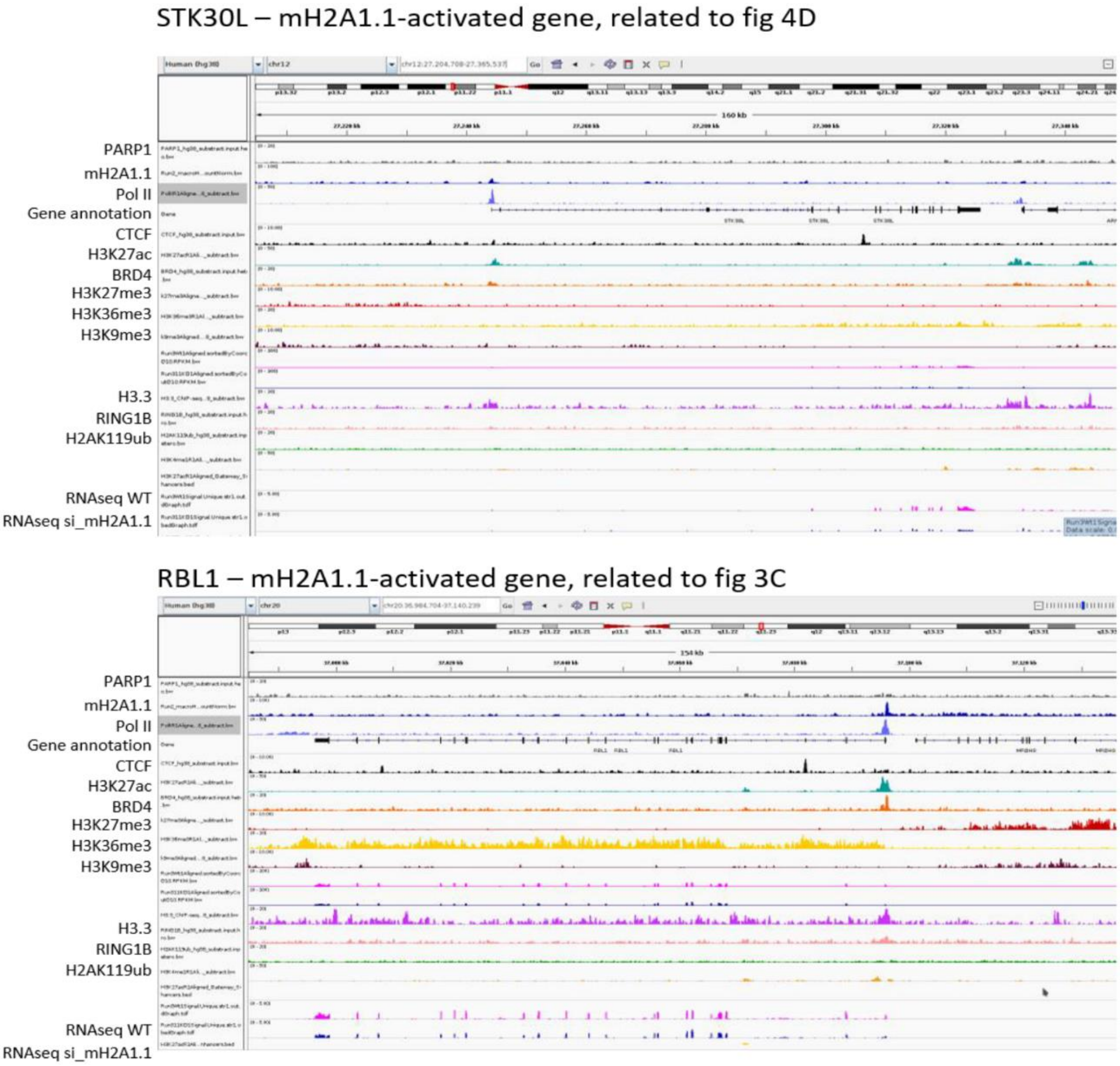

***Figure 1:** IGVs showing localization of PARP1 at two mH2A1.1-activated genes. IGVs show also others ChIP-seq data and RNAseq data.*

In our study, we identify many potential partners of mH2A1.1 which could participate in its positive effect on Pol II pausing release such as BRD4, RING1B, PCGF2, and H3.3. Although these analyses make one want to better characterize the underlying molecular mechanisms of these factors, we believe that testing the interconnection between all these partners is beyond the scope of this article.

2- While the epigenetic characterization of the TNBC cell line is expansive, the absence of inclusion of a macroH2A1.2 ChIP-Seq experiment, or comparison to existing macroH2A1.2 ChIP-Seq data (if available in the same cell line), is a significant limitation, as this would help corroborate the proposed, variant-specific effects described here, particularly at promoter TSSs. Is accumulation at TSSs specific to macroH2A1.1?

- *Unfortunately, we were unable to test the competition hypothesis, since, in our hands, all tested antibodies failed to specifically detect mH2A1.2 in chromatin immunoprecipitation (Bystricky and Buschbeck labs)(Hurtado-Bages et al., 2020). Only one research group did ChIP-seq of mH2A1.2 but they used their own mH2A1.2-specific antibody (Lavigne et al., 2015; Pliatska et al., 2018).*

© 2022. Published by The Company of Biologists under the terms of the Creative Commons Attribution License (https://creativecommons.org/licenses/by/4.0/).

3- It is counterintuitive that actively transcribed macroH2A1.1-enriched genes, which show mid to high gene expression levels (Fig 2C), have high levels of PolII pausing and little to no detectable gene body PolII (Fig 4A). This should be addressed in the discussion. Why would lower pausing in the presence of macroH2A1.1 cause lower expression?

- *Highly transcribed genes have been shown to be frequently Pol II paused genes (Core and Adelman, 2019). Fig. 2C shows that mH2A1.1 accumulates at actively transcribed genes. In this analysis, we did not discriminate paused and not paused genes. The role of mH2A1.1 at all of these genes can be inhibitory or stimulatory (Fig. 3). Yet, only genes activated by mH2A1.1 (AG) have a significantly elevated pausing index (Fig. 4B, C). Expression of these genes is lowered in the absence of mH2A1.1 likely because Pol II is no longer properly released (greater pausing). As a matter of fact, we demonstrate that the presence of mH2A1.1 seems to stimulate pause release to boost expression. Hence, in its absence, pausing is increased and leads to lower expression.*

4- Details antibody generation need to be added to the methods section (antigen, host, etc)

- *The method section refers to the host - rabbit - and gives the peptide sequence used. Figure S2 shows controls and complete characterization. We have added the contact information of the company that generated the antibody to the methods section – lane 756. "Rabbit anti-mH2A1.1 antibody was generated according to immunization protocol from Agro-Bio - La Fierté Saint-Aubin – France."*

Minor comments:

1- How do the authors exclude that macroH2A1.1 enrichment at enhancers (or promoters) is not an artifact of pairing between promoters and enhancers, and hence reflecting enrichment at only one of the two paired sites? Additionally, the fraction of paired promoter / enhancers with and without macroH2A1.1 should be assessed. Are promoter /enhancer pairs enriched in macroH2A1.1?

- *Indeed, so-called indirect peaks could be due to crosslinking artifacts. However, indirect peaks are generally of very low intensity, which is not at all what we observe. To be certain, we compared the signal strengths from mH2A1.1 to enhancers and promoters bound by mH2A1.1 (**Figure 2**). As a control, we did the same analysis but with Pol II ChIP-seq data. We found that Pol II signal is much higher at promoters compared to enhancers as expected. However, we found quasi similar intensity signals for mH2A1.1 at enhancers and promoters. This strongly suggests that peaks observed at either enhancers or promoters correspond to direct association of mH2A1.1 to those sites and not "crosslinking artifacts".*

© 2022. Published by The Company of Biologists under the terms of the Creative Commons Attribution License (https://creativecommons.org/licenses/by/4.0/).

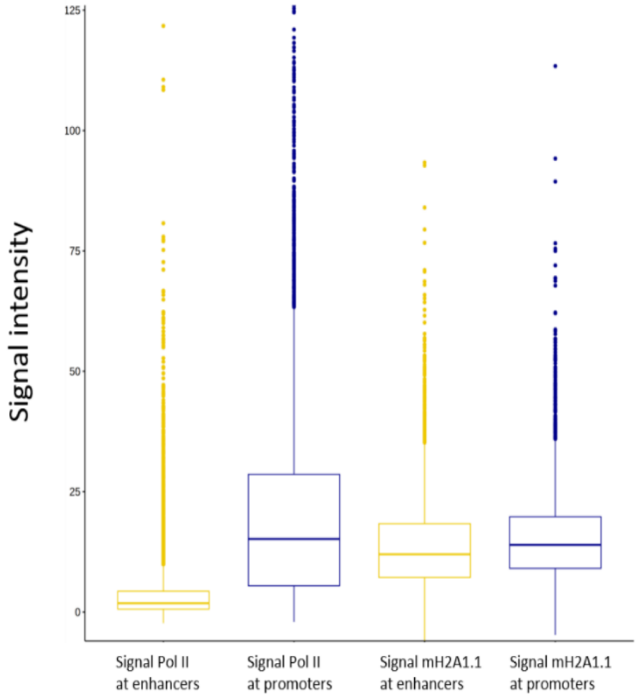

Figure 2: *Boxplot showing the signal intensity of ChIP- seq data on enhancers and promoters. Only enhancers and promoters bind either by Pol II or mH2A1.1 were taken for the analysis.*

*We found that around 50 % of enhancer/promoter contact sites are bound by mH2A1.1 either on the enhancer or the promoter. More details are given in **Table n° 1** and **Figure 3**. Of note, those fractions remain unchanged in the mH2A1.1 KD condition.*

			Fraction of interaction (%)		Comments
Number of interaction	WT	si_mH2A1.1	WT	si_mH2A1.1	
All interaction	15610	16515	100	100	
Interaction enh/pro	3869	3980	24,785394	24,0993037	fraction of all interaction
Interaction enh/pro no mH2A1.1	1942	2002	50,1938485	50,3015075	fraction of interaction enh/pro
Interaction enh/pro mH2A1.1 only at pro	333	395	8,60687516	9,92462312	fraction of interaction enh/pro
Interaction enh/pro mH2A1.1 only at enh	762	734	19,6950116	18,4422111	fraction of interaction enh/pro
Interaction enh/pro mH2A1.1 at enh & pro	832	849	21,5042647	21,3316583	fraction of interaction enh/pro
Interaction enh/pro mH2A1.1 at enh or pro	1927	1978	49,8061515	49,6984925	fraction of interaction enh/pro

Table 1: *Table showing the number of interactions and the corresponding relative fractions, as indicated in control (WT) and mH2A1.1 KD (si_mH2A1.1) conditions. enh = enhancers, pro = promoters.*

© 2022. Published by The Company of Biologists under the terms of the Creative Commons Attribution License (https://creativecommons.org/licenses/by/4.0/).

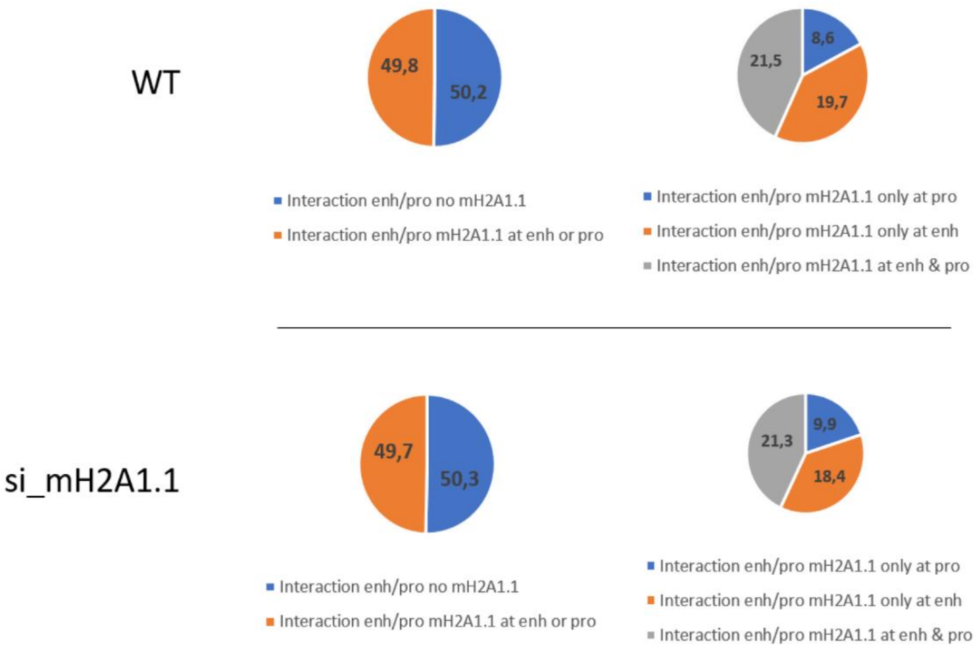

Figure 3: *Pie charts showing percentages of interaction between enhancers and promoters bound or not by mH2A1.1. Percentages are also summarized in the Table*

2- What fraction of genes with high PolII pause index show macroH2A1.1 enrichment? Is there a correlation to PARP, if so, what is the overlap? It may be helpful to speculate why this is particularly relevant at genes involved in cell migration.

- *This fraction is around 10 % of highly paused (HP) genes. The number of highly paused genes is n=2000 among 10546 genes for which we could determine a pausing index. It is logically a small fraction since mH2A1.1 does not define paused genes. Hence, the TSS of 226 genes highly in pause is bound by mH2A1.1 (**Figure 4**).*

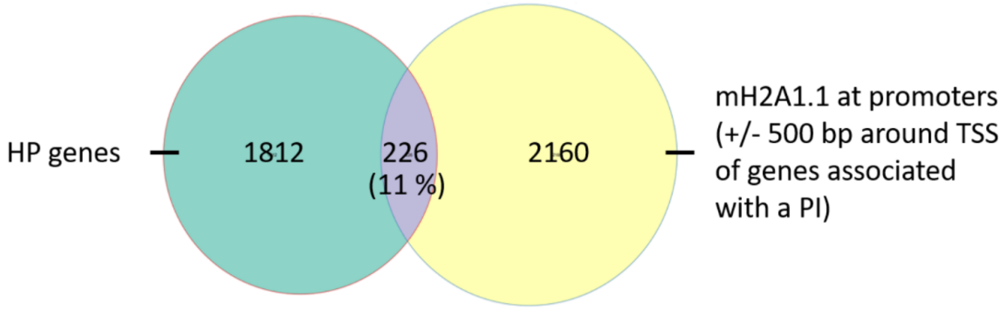

Figure 4: *Venn diagram showing the overlap between highly paused (HP) genes and mH2A1.1-related peaks at promoters of PI-associated genes.*

*We did not say that mH2A1.1 is enriched at paused genes but we demonstrate that the size of the mH2A1.1 peaks at the TSS of mH2A1.1 regulated genes is inversely proportional to the pausing level of the gene (**Figure 5**).*

© 2022. Published by The Company of Biologists under the terms of the Creative Commons Attribution License (https://creativecommons.org/licenses/by/4.0/).

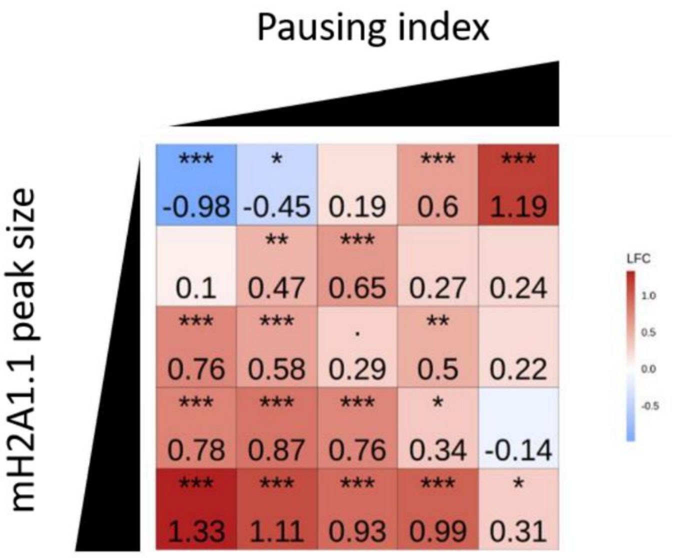

Figure 5: *Correlation heatmap showing enrichment of mH2A1.1 peaks at TSS (+/- 500bp) based on mH2A1.1 peak size and polymerase II pausing index (PI). mH2A1.1 narrow peaks at TSS are enriched at genes with a high PI whereas large mH2A1.1 peaks at TSS are enriched at genes with a low PI.*

*Of the 226 highly paused genes bound by mH2A1.1, 189 were bound by PARP1 (**Figure 6**). However, we found PARP1 (and also BRD4) associated with >80% of highly paused genes in general (**Figure 7**), so this is not a feature specific to mH2A1.1-bound genes.*

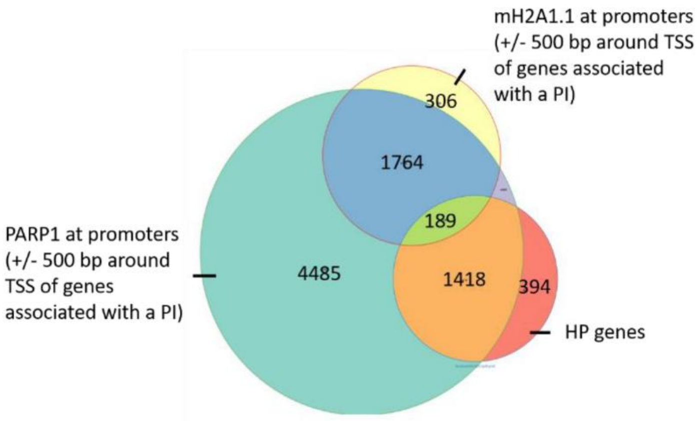

Figure 6: *Venn diagram showing the overlap between highly paused (HP) genes, mH2A1.1-related peaks at promoters of PI-associated genes and PARP1.*

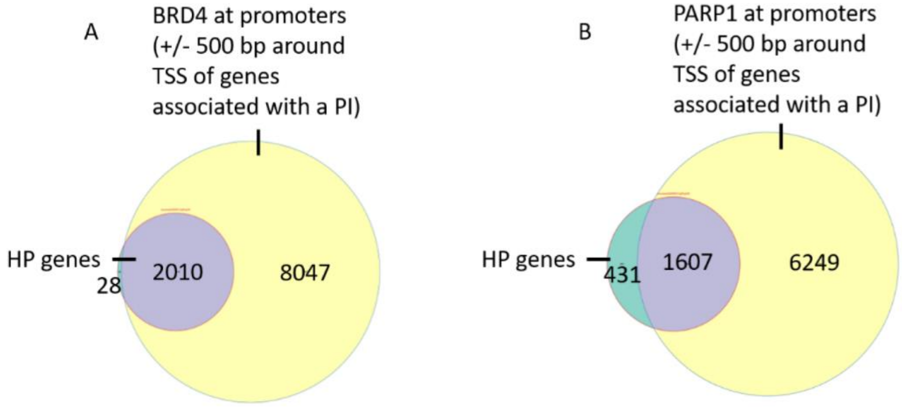

© 2022. Published by The Company of Biologists under the terms of the Creative Commons Attribution License (https://creativecommons.org/licenses/by/4.0/).

***Figure 7**: Venn diagram showing the overlap between highly paused (HP) genes with BRD4 (A) or PARP1 (B).*

We speculate that the role of mH2A1.1 at cells involved in migration is particularly relevant because their expression needs to be quickly adapted to changing environments in response to stimuli. Rapid gene regulation can be efficiently achieved through pause release mechanisms.

3- Based on previous work, the correlation of macroH2A1 / macroH2A1.1 with H3K27me3 is expected to be stronger. This should be discussed.

- *Fig. 1D and Fig. S3A of the article illustrate that most of mH2A1.1 (>50% of sites bound) associates and correlates with H3K27me3. We feel that this is described and discussed.*

4- Page 5: "macroH2A1.1 correlated with the level of [...] PolII, H3.3 and Brd4" Coincides would be a better choice, as correlation (PCC) between these markers has not been assessed at TSSs.

- *Thank you. The sentence was changed but the PCC are presented in Fig. 2A - Line 184. "At promoters, mH2A1.1 distribution inversely coincided with heterochromatin marks (**Fig. 2A**, B)."*

5- Is macroH2A1.1 subject to "pruning as recently described by PMID 30291361?

- *We did not test it. Over large domains, this could be possible, in particular since pruning was shown for mH2A1 without distinguishing isoforms (Sun et al, 2018). At regions where mH2A1.1 binding was detected as narrow peaks, pruning appears unlikely and deposition may require a different mechanism such as chaperone-mediated deposition independently of the genome duplication.*

References:

Bohm, V., Hieb, A.R., Andrews, A.J., Gansen, A., Rocker, A., Toth, K., Luger, K., and Langowski, J. (2011). Nucleosome accessibility governed by the dimer/tetramer interface. Nucleic acids research *39*, 3093-3102.

Chen, H., Ruiz, P.D., Novikov, L., Casill, A.D., Park, J.W., and Gamble, M.J. (2014). MacroH2A1.1 and PARP-1 cooperate to regulate transcription by promoting CBP-mediated H2B acetylation. Nature structural & molecular biology *21*, 981-989.

Core, L., and Adelman, K. (2019). Promoter-proximal pausing of RNA polymerase II: a nexus of gene regulation. Genes & development *33*, 960-982.

Gamble, M.J., Frizzell, K.M., Yang, C., Krishnakumar, R., and Kraus, W.L. (2010). The histone variant macroH2A1 marks repressed autosomal chromatin, but protects a subset of its target genes from silencing. Genes & development *24*, 21-32.

Gibson, B.A., Zhang, Y., Jiang, H., Hussey, K.M., Shrimp, J.H., Lin, H., Schwede, F., Yu, Y., and Kraus, W.L. (2016). Chemical genetic discovery of PARP targets reveals a role for PARP-1 in transcription elongation. Science *353*, 45-50.

Hurtado-Bages, S., Posavec Marjanovic, M., Valero, V., Malinverni, R., Corujo, D., Bouvet, P., Lavigne, A.C., Bystricky, K., and Buschbeck, M. (2020). The Histone Variant MacroH2A1 Regulates Key Genes for Myogenic Cell Fusion in a Splice-Isoform Dependent Manner. Cells *9*.

Jin, C., Zang, C., Wei, G., Cui, K., Peng, W., Zhao, K., and Felsenfeld, G. (2009). H3.3/H2A.Z double variant-containing nucleosomes mark 'nucleosome-free regions' of active promoters and other regulatory regions. Nature genetics *41*, 941-945.

Lavigne, A.C., Castells, M., Mermet, J., Kocanova, S., Dalvai, M., and Bystricky, K. (2014). Increased macroH2A1.1 expression correlates with poor survival of triple-negative breast cancer patients. PloS one *9*, e98930.

Lavigne, M.D., Vatsellas, G., Polyzos, A., Mantouvalou, E., Sianidis, G., Maraziotis, I., Agelopoulos, M., and Thanos, D. (2015). Composite macroH2A/NRF-1 Nucleosomes Suppress Noise and Generate Robustness in Gene Expression. Cell reports *11*, 1090-1101.

© 2022. Published by The Company of Biologists under the terms of the Creative Commons Attribution License (https://creativecommons.org/licenses/by/4.0/).

Pliatska, M., Kapasa, M., Kokkalis, A., Polyzos, A., and Thanos, D. (2018). The Histone Variant MacroH2A Blocks Cellular Reprogramming by Inhibiting Mesenchymal-to-Epithelial Transition. Molecular and cellular biology *38*.
Xu, C., Xu, Y., Gursoy-Yuzugullu, O., and Price, B.D. (2012). The histone variant macroH2A1.1 is recruited to DSBs through a mechanism involving PARP1. FEBS letters *586*, 3920-3925.
Yildirim, O., Hung, J.H., Cedeno, R.J., Weng, Z., Lengner, C.J., and Rando, O.J. (2014). A system for genome-wide histone variant dynamics in ES cells reveals dynamic MacroH2A2 replacement at promoters. PLoS genetics *10*, e1004515.

Second decision letter

MS ID#: JOCES/2021/259456

MS TITLE: The histone variant macroH2A1.1 regulates RNA Polymerase II paused genes within defined chromatin interaction landscapes.

AUTHORS: Ludmila Recoules, Alexandre Heurteau, Raynal Flavien, Nezih Karasu, Fatima Moutahir, Fabienne Bejjani, Isabell Jariel-Encontre, Olivier Cuvier, Tom Sexton, Anne-Claire Lavigne, and Kerstin Bystricky
ARTICLE TYPE: Research Article

We have now reached a decision on the above manuscript.

To see the reviewers' reports and a copy of this decision letter, please go to: https://submit-jcs.biologists.org and click on the 'Manuscripts with Decisions' queue in the Author Area. (Corresponding author only has access to reviews.)

As you will see, the reviewers gave favourable reports but Reviewer 1 raised some critical points that will require textual amendments to your manuscript. I hope that you will be able to carry these out because I would like to be able to accept your paper.

We are aware that you may be experiencing disruption to the normal running of your lab that makes experimental revisions challenging. If it would be helpful, we encourage you to contact us to discuss your revision in greater detail. Please send us a point-by-point response indicating where you are able to address concerns raised (either experimentally or by changes to the text) and where you will not be able to do so within the normal timeframe of a revision. We will then provide further guidance. Please also note that we are happy to extend revision timeframes as necessary.

Please ensure that you clearly highlight all changes made in the revised manuscript. Please avoid using 'Tracked changes' in Word files as these are lost in PDF conversion.

I should be grateful if you would also provide a point-by-point response detailing how you have dealt with the points raised by the reviewers in the 'Response to Reviewers' box. Please attend to all of the reviewers' comments. If you do not agree with any of their criticisms or suggestions please explain clearly why this is so.

Reviewer 1

Advance summary and potential significance to field

The authors have provided thorough and reasonable responses to most of the concerns raised in the previous review. While several of the study's limitations remain, the findings are important and appropriate for Journal of Cell Science after some adjustments (see below).

© 2022. Published by The Company of Biologists under the terms of the Creative Commons Attribution License (https://creativecommons.org/licenses/by/4.0/).

Comments for the author

1)Given that PARP1 is associated with ~80% of highly paused genes and that its enrichment pattern at highly paused versus unpaused genes is similar to that of macroH2A1.1 (Fig 4A), it is tempting to speculate that PARP1 is at least one factor that may help macroH2A1.1 to accumulate at HP genes. PARP1 impact on macroH2A1.1 ChIP has not been investigated by Hurtado-Bages et al. and one of 5 genes tested was responsive to PARP inactivation in this study. PARP dependence should be discussed if not tested as a possible explanation for macroH2A1.1 at nucleosome-free TSS regions.
2) In addition to the papers cited in the rebuttal, macroH2A1.2 ChIP has been reported in K562 cells using a commercially available antibody – specificity in this cell line was demonstrated via knockdown (PMID 29249653). While perhaps beyond the scope of this manuscript, a side-by-side comparison in the same cell line would add substance to the discussion of potential splice variant-specific functions.

Reviewer 2

Advance summary and potential significance to field

already reviewed

Comments for the author

Most of the concerns raised previously have been taken into account and the manuscript has now been much improved. I can therefore recommend this manuscript for publication.

Second revision

Author response to reviewers' comments

First round of reviews

Reviewer #1 (Evidence, reproducibility and clarity (Required)):

Summary:

Here the authors used a triple negative breast cancer (TNBC) cell line (MDA-MB231) expressing high levels of macroH2A1.1 to obtain a detailed map of genomic regions bound by this histone and to highlight the functional significance of the corresponding particular genomic localization.
Technically macroH2A1.1 as well as Pol II and a series of histone marks (H3K27ac, H3K4me1, H3K4me3, H3K36me3) were mapped following the authors' own ChIPs. They also included published ChIP-seq data (BRD4, RING1B, PCFG2 and H2AK119ub) in their analyses to enlarge their view of macroH2A1.1 chromatin landscape in the studied cell line.

Their main conclusions are summarized below.
macroH2A1.1 is predominantly associated with "facultative-like heterochromatin". macroH2A1.1 binds enhancer and promoter regions.
macroH2A1.1 enhances gene expression by releasing Pol II pause at a subset of genes, including a group of anti-migratory genes.
macroH2A1.1 binds enhancers and super-enhancers in association with BRD4 and RING1B.
macroH2A1.1 is not significantly involved in genomic interactions between promoters and other genomic regions at the mH2A1.1 regulated genes, and the role of mH2A1.1 in Pol II release does not involve a modulation of chromatin looping.

Major comments:
1 - This study comprehensively describes the genome-wide map of mH2A1.1 in a single human breast cancer cell line and considers some functional consequences of these observations following

© 2022. Published by The Company of Biologists under the terms of the Creative Commons Attribution License (https://creativecommons.org/licenses/by/4.0/).

mH2A1.1 knock-down. These approaches allowed the authors to draw the interesting conclusions summarized above.
The experimental approaches and data analyses appear sound and they convincingly back the conclusions.
However, it is not clear if the described properties and activities of mH2A1.1 are specific to this cell line, to triple negative breast cancer, or whether they could stand generally in all systems expressing significant amounts of mH2A1.1.
Although it could be too much to carry out this research in several cell systems in parallel, it would have been very useful, if the authors could at least use the available mH2A1.1 mapping data to check some of their conclusions on different cancer and non-cancer cell lines and in various physiological contexts.

- *We have included a new paragraph in the manuscript to discuss this aspect of our work - Line 365*
  "The fact that mH2A1.1 binds specifically to the TSS of genes it activates in MDA- MB231 cells is particularly intriguing here. The only other study which included an analysis of the genomic localization of this histone variant was performed in the murine muscle cell line C2C12 (Hurtado-Bagès et al., 2020). In this cell line, mH2A1.1 recruitment occurred upstream of the TSS of these genes but also, in contrast to AG in MDA-MB231 cells, over the gene body. Analysis of the genomic distribution of other macro histone variants shows cell type specific patterns: Lavigne et al, reported binding of the mH2A1.2 isoform specifically to the TSS of mH2A1-regulated genes in two cancer cell types, HeLa and Namalwa cells (M. D. Lavigne et al., 2015). Comparison of genomic sites bound by mH2A1.2 nucleosomes revealed only a small overlap between HeLa and Namalwa cells. Similarly, mH2A1 and mH2A2 association with the TSS was also observed in human et mouse embryonic stem cells (Pliatska et al., 2018; Yildirim et al., 2014). Therefore, we propose that mH2A variants are differentially recruited to regulatory sites depending on carcinogenic and differentiation state of the cells. In breast cancers, recruitment and thus the roles of mH2A1 variants must be subtype specific. The newly identified binding pattern of mH2A1.1 we report here would thus be TNBC specific, because it was not identified in luminal breast cancer cell lines (Gamble et al., 2010). It could then explain why we found a correlation between mH2A1.1 expression levels and survival rates only in TNBC patients (A.-C. Lavigne et al., 2014)."

2- An intriguing observation is the accumulation of mH2A1.1 at the nucleosome free regions (i e Fig. 2E). This is an unexpected observation. Could the authors better characterize this phenomenon?
Do the authors know any histone variant that accumulate at the active gene NFRs? Is the association of mH2A with NFR observed in any other system? This observation should be highlighted and specifically discussed.

- *We and others (Lavigne et al., 2015; Yildirim et al., 2014) observed mH2As in a manner around the TSS to place the maximum binding to the nucleosome free region (NFR). Different hypotheses could explain those results. The first one could be that mH2A isoforms are not incorporated into the chromatin and bind the NFR as a transcription factor. Indeed, upon DSBs, Xu et al, 2012 showed that mH2A1 binds DSB chromatin without being incorporated into a nucleosome (Xu et al., 2012). However, Lavigne et al, detected TSS-bound mH2A1.2 by native ChIP, excluding in part this hypothesis (Lavigne et al., 2015). A second hypothesis could be that mH2As are incorporated into the two adjacent nucleosomes of the TSSs but their macro domain could be joined at the NFR, explaining the higher signal at the NFR obtained compared to the two adjacent nucleosomes. Yet another hypothesis could be that mH2A isoforms are incorporated into "labile nucleosomes" frequently found at the not really 'NFR' and only observed using low salt ChIP experiments (done for all the mentioned publications and ours here), as similarly to H3.3 and H2AZ (Jin et al., 2009). Future analysis will be necessary to discriminate between these hypothesis.*

3 - The authors should consider that the knock-down of mH2A1.1 may generate local histone under-dosage. Indeed, if the lack of mH2A1.1 is not compensated by other H2As, a modified chromatin may appear at regions originally containing mH2A1.1. Since histones combines structural and regulatory roles, it is important that the authors highlight in their discussions that the

© 2022. Published by The Company of Biologists under the terms of the Creative Commons Attribution License (https://creativecommons.org/licenses/by/4.0/).

phenotypes associated with KD/KO approaches could also reflect defective chromatin organizations.

- *Indeed, we can not exclude that depletion of the histone variant mH2A1.1 or mH2A1.2 or any other histone creates labile nucleosomes, possibly tetrasomes (Bohm et al., 2011) which may change accessibility and affect transcriptional activity indirectly. However, siRNA treatment was performed over 72h including at least 2 or 3 replication cycles during which H2A core histone is expressed and incorporated, most likely, at sites which originally contained mH2A1. Moreover, since looping is not affected, it seems unlikely that chromatin structure massively changes at the studied sites.*

Reviewer #3 (Evidence, reproducibility and clarity (Required)):

Summary:

This manuscript by Bystricky and colleagues describes a thorough investigation of the chromatin associations and functional implications of the macroH2A1.1 histone variant. MacroH2A1.1 is one of two alternative splice variants of the macro-histone variant macroH2A1, the other being macroH2A1.2. This manuscript is to this reviewers knowledge the first to describe genome-wide mapping of macroH2A1.1 chromatin occupancy. To do so, the authors have generated and extensively validated a macroH2A1.1 splice variant-specific polyclonal antibody. Knowing the genomic regions that are affected by either macroH2A1 variant is anticipated to advance our understanding of their often distinct biological roles. In this manuscript, the authors use their ChIP-Seq findings to provide additional insight into macroH21.1-mediated regulation of gene expression. MacroH2A1.1 loss has been shown to both activate and repress gene expression, and the authors now propose a link between macroH2A1.1 intensity and the status of RNA Polymerase II (PolII) pausing at transcription start sites as a predictor of expression outcome. Genetic evidence for macroH2A1.1- dependent PolII pausing is compelling. A model involving looping between TSSs and macroH2A1.1-enriched enhancers is proposed. However, given that macroH2A1.1 does not alter loop formation, this adds more confusion than clarification. It is further unclear what accounts for the seemingly distinct modes of macroH2A1.1 accumulation at repressed and activated genes. The authors end with a demonstration of functional relevance for cell migration, which extends previous work by invoking a contribution of macroH2A1.1-dependent PolII pausing (e.g. PMID: 22542848).

Major comments:

1- The link between macroH2A1.1 at promoters, increased transcription and Pol II pausing is intriguing. However, little mechanistic detail is provided to explain how macroH2A1.1 is enriched at active promoters, and how it modulates PolII pausing. This is a major missed opportunity, particularly given the link between Pol II pausing and PARP1, which also interacts with macroH2A1.1 (e.g. PMID: 31940791). It would be interesting to investigate a possible relationship between PARP activity and recruitment macroH2A1.1 at TSSs. It is further unclear if macroH2A1.1 accumulation at these regions involves nucleosome incorporation, particularly since its accumulation at genes with paused PolII overlaps with nucleosome free regions.

- *We thank the reviewer for proposing to test the relationship between PARP1 and mH2A1.1 to explain its recruitment and its effect on transcriptional activation potentially due to the well known interaction between the two proteins (Chen et al., 2014) and the relationship between PARP1 and Pol II pausing (Gibson et al., 2016).*

*For several reasons, however, we chose not to investigate this aspect further in the present study. The first reason is that the genomic localization of PARP1 in MDA-MB 231 does not show a significant enrichment of this protein at mH2A1.1-target genes (**Figure 1**). The second reason is that Hurtado et al showed that the transcriptional activator effect of mH2A1.1 is independent of PARP1 in C2C12 muscle mouse cells (Hurtado-Bages et al., 2020).*

© 2022. Published by The Company of Biologists under the terms of the Creative Commons Attribution License (https://creativecommons.org/licenses/by/4.0/).

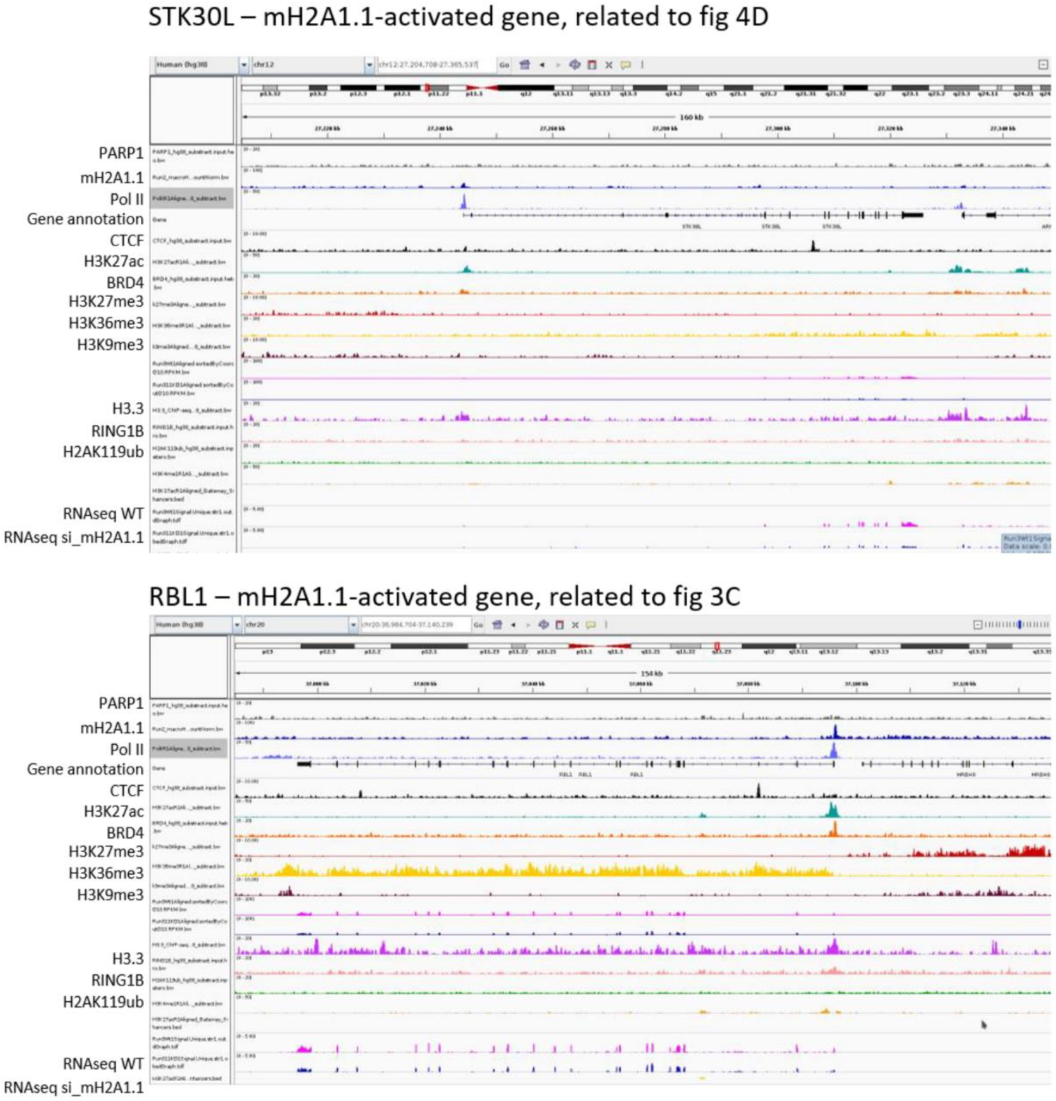

Figure 1*: IGVs showing localization of PARP1 at two mH2A1.1-activated genes. IGVs show also others ChIP-seq data and RNAseq data.*

In our study, we identify many potential partners of mH2A1.1 which could participate in its positive effect on Pol II pausing release such as BRD4, RING1B, PCGF2, and H3.3. Although these analyses make one want to better characterize the underlying molecular mechanisms of these factors, we believe that testing the interconnection between all these partners is beyond the scope of this article.

2- While the epigenetic characterization of the TNBC cell line is expansive, the absence of inclusion of a macroH2A1.2 ChIP-Seq experiment, or comparison to existing macroH2A1.2 ChIP-Seq data (if available in the same cell line), is a significant limitation, as this would help corroborate the proposed, variant-specific effects described here, particularly at promoter TSSs. Is accumulation at TSSs specific to macroH2A1.1?

- *Unfortunately, we were unable to test the competition hypothesis, since, in our hands, all tested antibodies failed to specifically detect mH2A1.2 in chromatin immunoprecipitation (Bystricky and Buschbeck labs)(Hurtado-Bages et al., 2020). Only one research group did ChIP-seq of mH2A1.2 but they used their own mH2A1.2-specific antibody (Lavigne et al., 2015; Pliatska et al., 2018).*

3- It is counterintuitive that actively transcribed macroH2A1.1-enriched genes, which show mid to high gene expression levels (Fig 2C), have high levels of PolII pausing and little to no

© 2022. Published by The Company of Biologists under the terms of the Creative Commons Attribution License (https://creativecommons.org/licenses/by/4.0/).

detectable gene body PolII (Fig 4A). This should be addressed in the discussion. Why would lower pausing in the presence of macroH2A1.1 cause lower expression?

- *Highly transcribed genes have been shown to be frequently Pol II paused genes (Core and Adelman, 2019). Fig. 2C shows that mH2A1.1 accumulates at actively transcribed genes. In this analysis, we did not discriminate paused and not paused genes. The role of mH2A1.1 at all of these genes can be inhibitory or stimulatory (Fig. 3). Yet, only genes activated by mH2A1.1 (AG) have a significantly elevated pausing index (Fig. 4B, C). Expression of these genes is lowered in the absence of mH2A1.1 likely because Pol II is no longer properly released (greater pausing). As a matter of fact, we demonstrate that the presence of mH2A1.1 seems to stimulate pause release to boost expression. Hence, in its absence, pausing is increased and leads to lower expression.*

4- Details antibody generation need to be added to the methods section (antigen, host, etc)

- *The method section refers to the host - rabbit - and gives the peptide sequence used. Figure S2 shows controls and complete characterization. We have added the contact information of the company that generated the antibody to the methods section – lane 756. "Rabbit anti-mH2A1.1 antibody was generated according to immunization protocol from Agro-Bio - La Fierté Saint-Aubin – France."*

Minor comments:
1- How do the authors exclude that macroH2A1.1 enrichment at enhancers (or promoters) is not an artifact of pairing between promoters and enhancers, and hence reflecting enrichment at only one of the two paired sites? Additionally, the fraction of paired promoter / enhancers with and without macroH2A1.1 should be assessed. Are promoter /enhancer pairs enriched in macroH2A1.1?

- *Indeed, so-called indirect peaks could be due to crosslinking artifacts. However, indirect peaks are generally of very low intensity, which is not at all what we observe. To be certain, we compared the signal strengths from mH2A1.1 to enhancers and promoters bound by mH2A1.1 (**Figure 2**). As a control, we did the same analysis but with Pol II ChIP-seq data. We found that Pol II signal is much higher at promoters compared to enhancers as expected. However, we found quasi similar intensity signals for mH2A1.1 at enhancers and promoters. This strongly suggests that peaks observed at either enhancers or promoters correspond to direct association of mH2A1.1 to those sites and not "crosslinking artifacts"*

© 2022. Published by The Company of Biologists under the terms of the Creative Commons Attribution License (https://creativecommons.org/licenses/by/4.0/).

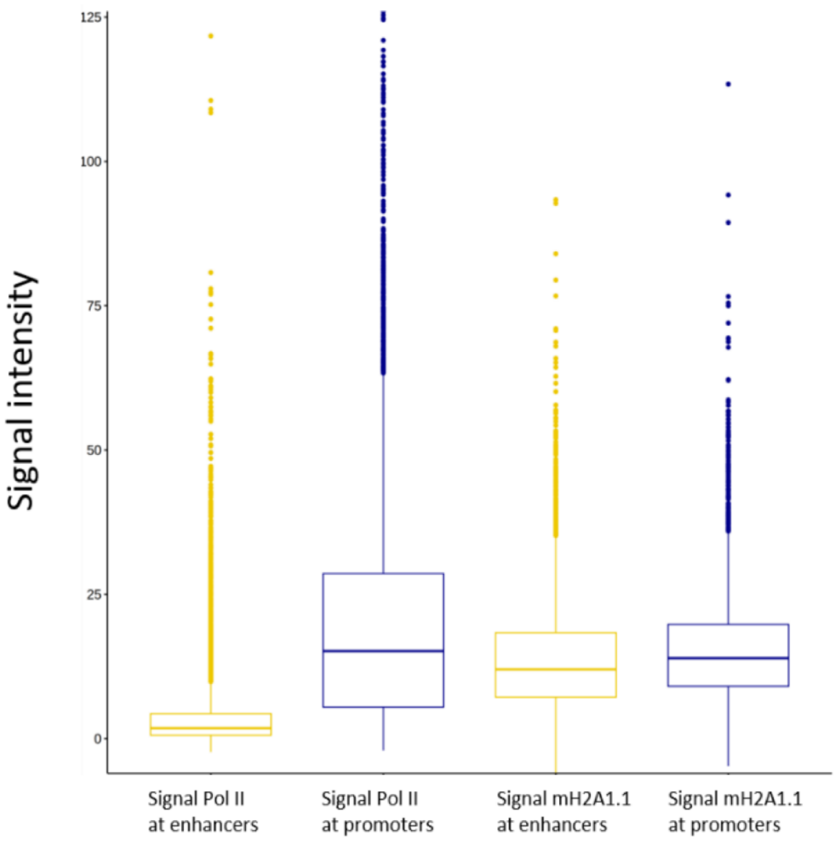

***Figure 2:** Boxplot showing the signal intensity of ChIP-seq data on enhancers and promoters. Only enhancers and promoters bind either by Pol II or mH2A1.1 were taken for the analysis.*

*We found that around 50 % of enhancer/promoter contact sites are bound by mH2A1.1 either on the enhancer or the promoter. More details are given in **Table n° 1** and **Figure 3**. Of note, those fractions remain unchanged in the mH2A1.1 KD condition.*

			Fraction of interaction (%)		Comments
Number of interaction	WT	si_mH2A1.1	WT	si_mH2A1.1	
All interaction	15610	16515	100	100	
Interaction enh/pro	3869	3980	24,785394	24,0993037	fraction of all interaction
Interaction enh/pro no mH2A1.1	1942	2002	50,1938485	50,3015075	fraction of interaction enh/pro
Interaction enh/pro mH2A1.1 only at pro	333	395	8,60687516	9,92462312	fraction of interaction enh/pro
Interaction enh/pro mH2A1.1 only at enh	762	734	19,6950116	18,4422111	fraction of interaction enh/pro
Interaction enh/pro mH2A1.1 at enh & pro	832	849	21,5042647	21,3316583	fraction of interaction enh/pro
Interaction enh/pro mH2A1.1 at enh or pro	1927	1978	49,8061515	49,6984925	fraction of interaction enh/pro

© 2022. Published by The Company of Biologists under the terms of the Creative Commons Attribution License (https://creativecommons.org/licenses/by/4.0/).

***Table 1**: Table showing the number of interactions and the corresponding relative fractions, as indicated in control (WT) and mH2A1.1 KD (si_mH2A1.1) conditions. enh = enhancers, pro = promoters.*

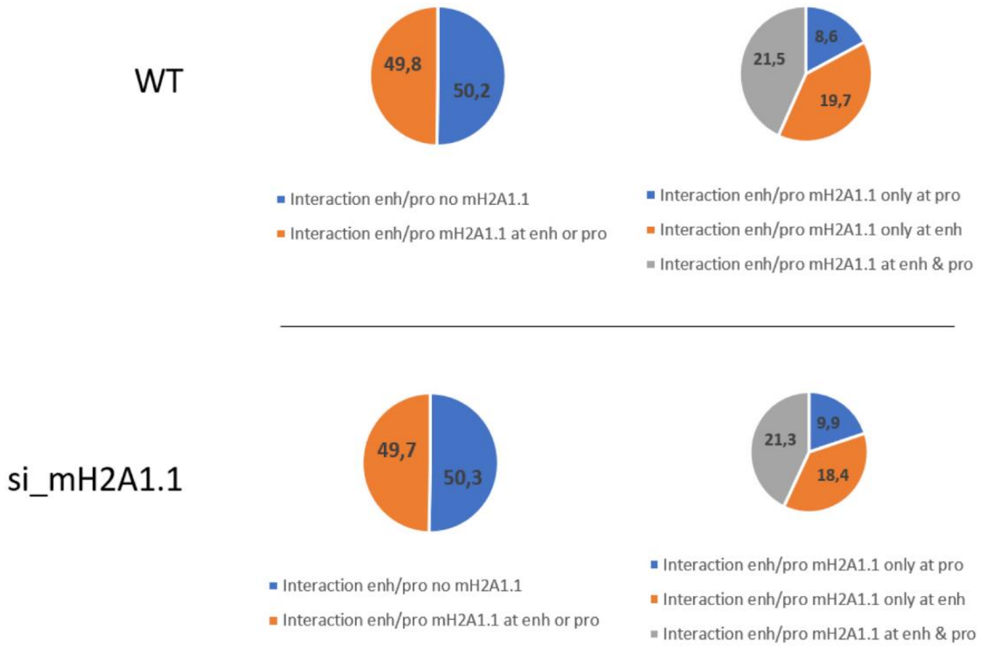

***Figure 3**: Pie charts showing percentages of interaction between enhancers and promoters bound or not by mH2A1.1. Percentages are also summarized in the Table*

2- What fraction of genes with high PolII pause index show macroH2A1.1 enrichment? Is there a correlation to PARP, if so, what is the overlap? It may be helpful to speculate why this is particularly relevant at genes involved in cell migration.

- *This fraction is around 10 % of highly paused (HP) genes. The number of highly paused genes is n=2000 among 10546 genes for which we could determine a pausing index. It is logically a small fraction since mH2A1.1 does not define paused genes. Hence, the TSS of 226 genes highly in pause is bound by mH2A1.1 (**Figure 4**).*

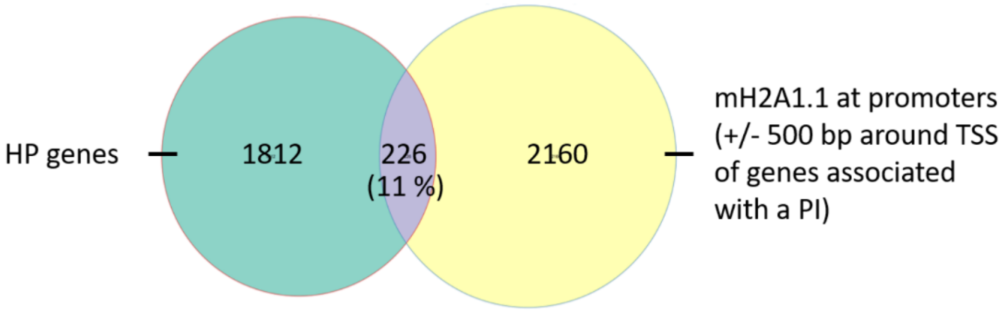

***Figure 4**: Venn diagram showing the overlap between highly paused (HP) genes and mH2A1.1-related peaks at promoters of PI-associated genes.*

*We did not say that mH2A1.1 is enriched at paused genes but we demonstrate that the size of the mH2A1.1 peaks at the TSS of mH2A1.1 regulated genes is inversely proportional to the pausing level of the gene (**Figure 5**).*

© 2022. Published by The Company of Biologists under the terms of the Creative Commons Attribution License (https://creativecommons.org/licenses/by/4.0/).

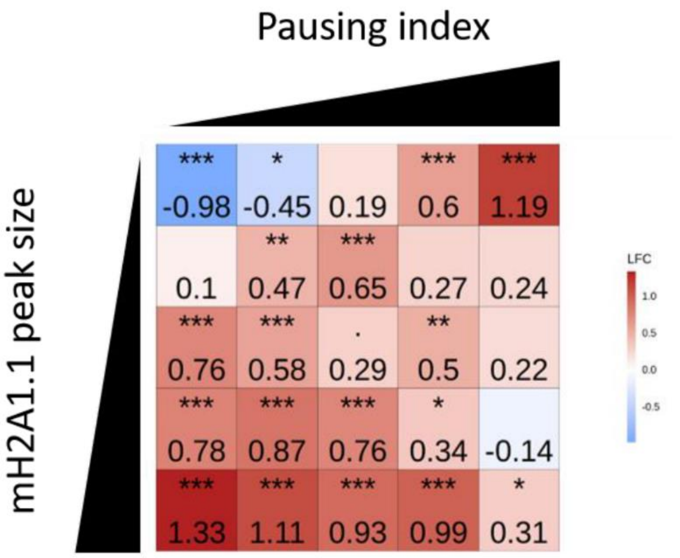

Figure 5: *Correlation heatmap showing enrichment of mH2A1.1 peaks at TSS (+/- 500bp) based on mH2A1.1 peak size and polymerase II pausing index (PI). mH2A1.1 narrow peaks at TSS are enriched at genes with a high PI whereas large mH2A1.1 peaks at TSS are enriched at genes with a low PI.*

*Of the 226 highly paused genes bound by mH2A1.1, 189 were bound by PARP1 (**Figure 6**). However, we found PARP1 (and also BRD4) associated with >80% of highly paused genes in general (**Figure 7**), so this is not a feature specific to mH2A1.1-bound genes.*

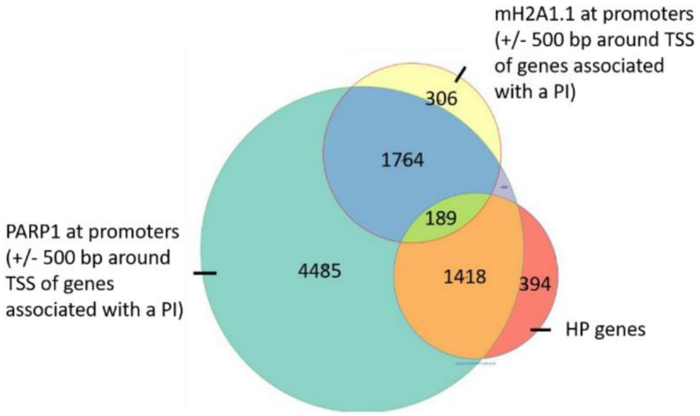

Figure 6: *Venn diagram showing the overlap between highly paused (HP) genes, mH2A1.1-related peaks at promoters of PI-associated genes and PARP1.*

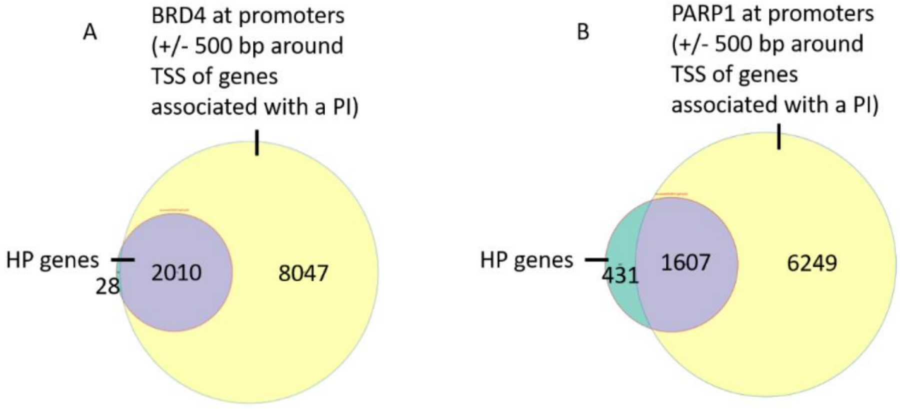

Figure 7: *Venn diagram showing the overlap between highly paused (HP) genes with BRD4 (A) or PARP1 (B).*

© 2022. Published by The Company of Biologists under the terms of the Creative Commons Attribution License (https://creativecommons.org/licenses/by/4.0/).

We speculate that the role of mH2A1.1 at cells involved in migration is particularly relevant because their expression needs to be quickly adapted to changing environments in response to stimuli. Rapid gene regulation can be efficiently achieved through pause release mechanisms.

3- Based on previous work, the correlation of macroH2A1 / macroH2A1.1 with H3K27me3 is expected to be stronger. This should be discussed.

- *Fig. 1D and Fig. S3A of the article illustrate that most of mH2A1.1 (>50% of sites bound) associates and correlates with H3K27me3. We feel that this is described and discussed.*

4- Page 5: "macroH2A1.1 correlated with the level of [...] PolII, H3.3 and Brd4" Coincides would be a better choice, as correlation (PCC) between these markers has not been assessed at TSSs.

- *Thank you. The sentence was changed but the PCC are presented in Fig. 2A - Line 180. "At promoters, mH2A1.1 distribution inversely coincided with heterochromatin marks (**Fig. 2A, B**)."*

5- Is macroH2A1.1 subject to "pruning as recently described by PMID 30291361?

- *We did not test it. Over large domains, this could be possible, in particular since pruning was shown for mH2A1 without distinguishing isoforms (Sun et al, 2018). At regions where mH2A1.1 binding was detected as narrow peaks, pruning appears unlikely and deposition may require a different mechanism such as chaperone-mediated deposition independently of the genome duplication.*

<u>Second round of reviews</u>

Reviewer #1
Advance Summary and Potential Significance to Field.
The authors have provided thorough and reasonable responses to most of the concerns raised in the previous review. While several of the study's limitations remain, the findings are important and appropriate for Journal of Cell Science after some adjustments (see below).

Comments for the Author
1 - Given that PARP1 is associated with ~80% of highly paused genes and that its enrichment pattern at highly paused versus unpaused genes is similar to that of macroH2A1.1 (Fig 4A), it is tempting to speculate that PARP1 is at least one factor that may help macroH2A1.1 to accumulate at HP genes. PARP1 impact on macroH2A1.1 ChIP has not been investigated by Hurtado-Bages et al. and one of 5 genes tested was responsive to PARP inactivation in this study. PARP dependence should be discussed if not tested as a possible explanation for macroH2A1.1 at nucleosome-free TSS regions.

- *Despite the fact that we find an association between PARP1 and TSS of paused genes (**Fig. 7B** and article Fig. 3C), we do not find a strong association of PARP1 on mH2A1.1- regulated genes, in particular at mH2A1.1-activated genes that have a high pausing index (**Fig. 1**).*

© 2022. Published by The Company of Biologists under the terms of the Creative Commons Attribution License (https://creativecommons.org/licenses/by/4.0/).

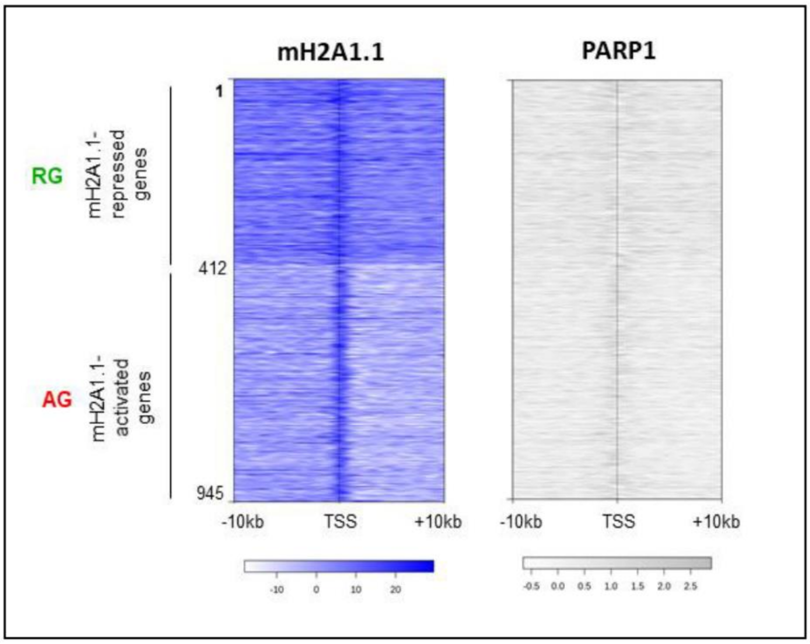

Figure 1*: Heatmap profiles showing relative enrichment of mH2A1.1 and PARP1 around the TSS (+/- 10 kb) of mH2A1.1-regulated genes. On the top, mH2A1.1- repressed genes (1 to 412, n=412), on the bottom, mH2A1.1-activated genes (412 to 945, n=533). Color intensity reflects level of ChIP-seq enrichment. Heatmaps are oriented.*

*Therefore, we think that it is difficult to speculate that PARP1 participates to the activator function of mH2A1.1, even though we cannot exclude that PARP1 participates in some functions of mH2A1.1 in MDA-MB 231 cells, for instance in heterochromatin domains in which PARP1 and mH2A1.1 are associated (**Fig. S3A**).*
Moreover, Hurtado et al., tested the effect of PARP1 on the expression levels of 5 mH2A1.1-activated genes using PARP1-siRNA. However, they showed that only one gene (Fn1) was significantly regulated by PARP1, and unlike mH2A1.1, PARP1 repressed its expression in myogenic C2C12 cells (**Article Hurtado et al., 2020, Fig. 4D**). Thus, this article proposes that the positive effect of mH2A1.1 in this cell line is mainly independent of PARP1, as could be also the case in MDA-MB 231 cells. This result could be a cell type specificity, as Gamble et al., showed that PARP1 positively regulates the expression of mH2A1.1-regulated genes in IMR90 cells (Gamble et al.,2010).

2 - In addition to the papers cited in the rebuttal, macroH2A1.2 ChIP has been reported in K562 cells using a commercially available antibody – specificity in this cell line was demonstrated via knockdown (PMID 29249653).
While perhaps beyond the scope of this manuscript, a side-by-side comparison in the same cell line would add substance to the discussion of potential splice variant-specific functions.

- *We thank the reviewer for providing this reference. We agree that it would be interesting to evaluate the respective recruitment of mH2A1.1 and mH2A1.2 in MDA-MB231 cells as a follow up. Thus, once verified that there is no cross reaction of this antibody with macroH2A1.1, it would be interesting to use it in MDA-MB231 cells. We have added a sentence stating this in the result section (lane 296). But as the reviewer indicates, this is beyond the scope of this manuscript, which focuses on the localization and identification of transcriptional functions of mH2A1.1.*

Reviewer #2
Advance Summary and Potential Significance to Field.
already reviewed

Comments for the Author
Most of the concerns raised previously have been taken into account and the manuscript has now been much improved. I can therefore recommend this manuscript for publication.

© 2022. Published by The Company of Biologists under the terms of the Creative Commons Attribution License (https://creativecommons.org/licenses/by/4.0/).

References:

Bohm, V., Hieb, A.R., Andrews, A.J., Gansen, A., Rocker, A., Toth, K., Luger, K., and Langowski, J. (2011). Nucleosome accessibility governed by the dimer/tetramer interface. Nucleic acids research *39*, 3093-3102.

Chen, H., Ruiz, P.D., Novikov, L., Casill, A.D., Park, J.W., and Gamble, M.J. (2014). MacroH2A1.1 and PARP-1 cooperate to regulate transcription by promoting CBP-mediated H2B acetylation. Nature structural & molecular biology *21*, 981-989.

Core, L., and Adelman, K. (2019). Promoter-proximal pausing of RNA polymerase II: a nexus of gene regulation. Genes & development *33*, 960-982.

Gamble, M.J., Frizzell, K.M., Yang, C., Krishnakumar, R., and Kraus, W.L. (2010). The histone variant macroH2A1 marks repressed autosomal chromatin, but protects a subset of its target genes from silencing. Genes & development *24*, 21-32.

Gibson, B.A., Zhang, Y., Jiang, H., Hussey, K.M., Shrimp, J.H., Lin, H., Schwede, F., Yu, Y., and Kraus, W.L. (2016). Chemical genetic discovery of PARP targets reveals a role for PARP-1 in transcription elongation. Science *353*, 45-50.

Hurtado-Bages, S., Posavec Marjanovic, M., Valero, V., Malinverni, R., Corujo, D., Bouvet, P., Lavigne, A.C., Bystricky, K., and Buschbeck, M. (2020). The Histone Variant MacroH2A1 Regulates Key Genes for Myogenic Cell Fusion in a Splice-Isoform Dependent Manner. Cells *9*.

Jin, C., Zang, C., Wei, G., Cui, K., Peng, W., Zhao, K., and Felsenfeld, G. (2009). H3.3/H2A.Z double variant-containing nucleosomes mark 'nucleosome-free regions' of active promoters and other regulatory regions. Nature genetics *41*, 941-945.

Lavigne, A.C., Castells, M., Mermet, J., Kocanova, S., Dalvai, M., and Bystricky, K. (2014). Increased macroH2A1.1 expression correlates with poor survival of triple-negative breast cancer patients. PloS one *9*, e98930.

Lavigne, M.D., Vatsellas, G., Polyzos, A., Mantouvalou, E., Sianidis, G., Maraziotis, I., Agelopoulos, M., and Thanos, D. (2015). Composite macroH2A/NRF-1 Nucleosomes Suppress Noise and Generate Robustness in Gene Expression. Cell reports *11*, 1090-1101.

Pliatska, M., Kapasa, M., Kokkalis, A., Polyzos, A., and Thanos, D. (2018). The Histone Variant MacroH2A Blocks Cellular Reprogramming by Inhibiting Mesenchymal-to-Epithelial Transition. Molecular and cellular biology *38*.

Xu, C., Xu, Y., Gursoy-Yuzugullu, O., and Price, B.D. (2012). The histone variant macroH2A1.1 is recruited to DSBs through a mechanism involving PARP1. FEBS letters *586*, 3920-3925.

Yildirim, O., Hung, J.H., Cedeno, R.J., Weng, Z., Lengner, C.J., and Rando, O.J. (2014). A system for genome-wide histone variant dynamics in ES cells reveals dynamic MacroH2A2 replacement at promoters. PLoS genetics *10*, e1004515.

Third decision letter

MS ID#: JOCES/2021/259456

MS TITLE: The histone variant macroH2A1.1 regulates RNA Polymerase II paused genes within defined chromatin interaction landscapes.

AUTHORS: Ludmila Recoules, Alexandre Heurteau, Flavien Raynal, Nezih Karasu, Fatima Moutahir, Fabienne Bejjani, Isabell Jariel-Encontre, Olivier Cuvier, Tom Sexton, Anne-Claire Lavigne, and Kerstin Bystricky
ARTICLE TYPE: Research Article

Thank you for sending your manuscript to Journal of Cell Science through Review Commons.

I am happy to tell you that your manuscript has been accepted for publication in Journal of Cell Science, pending standard ethics checks.

© 2022. Published by The Company of Biologists under the terms of the Creative Commons Attribution License (https://creativecommons.org/licenses/by/4.0/).
